# Supported σ‐Complexes of Li−C Bonds from Coordination of Monomeric Molecules of LiCH_3_, LiCH_2_CH_3_ and LiC_6_H_5_ to Mo≣Mo Bonds

**DOI:** 10.1002/anie.202116009

**Published:** 2022-01-11

**Authors:** Marina Pérez‐Jiménez, Jesús Campos, Jesús Jover, Santiago Álvarez, Ernesto Carmona

**Affiliations:** ^1^ Instituto de Investigaciones Químicas (IIQ) Departamento de Química Inorgánica and Centro de Innovación en Química Avanzada (ORFEO-CINQA) Consejo Superior de Investigaciones Científicas (CSIC) University of Sevilla Avda. Américo Vespucio, 49 41092 Sevilla Spain; ^2^ Department de Química Inorgànica I Orgànica Secció de Química Inorgànica Institut de Química Teòrica i Computacional Universitat de Barcelona Martí i Franquès 1–11 08028 Barcelona Spain

**Keywords:** Dimolybdenum Complexes, Methyllithium, Multiple Metal–Metal Bonds, Organolithium Reagents, σ-Bonding

## Abstract

LiCH_3_ and LiCH_2_CH_3_ react with the complex [Mo_2_(H)_2_(μ‐Ad^Dipp2^)_2_(thf)_2_] (**1⋅thf**) with coordination of two molecules of LiCH_2_R (R=H, CH_3_) and formation of complexes [Mo_2_{μ‐HLi(thf)CH_2_R}_2_(Ad^Dipp2^)_2_], **5⋅LiCH_3_** and **5⋅LiCH_2_CH_3_**, respectively (Ad^Dipp2^=HC(NDipp)_2_; Dipp=2,6‐^*i*^Pr_2_C_6_H_3_; thf=C_4_H_8_O). Due to steric hindrance, only one molecule of LiC_6_H_5_ adds to **1⋅thf** generating the complex [Mo_2_(H){μ‐HLi(thf)C_6_H_5_}(μ‐Ad^Dipp2^)_2_], (**4⋅LiC_6_H_5_**). Computational studies disclose the existence of five‐center six‐electron bonding within the H−Mo≣Mo−C−Li metallacycles, with a mostly covalent H−Mo≣Mo−C group and predominantly ionic Li−C and Li−H interactions. However, the latter bonds exhibit non‐negligible covalency, as indicated by X‐ray, computational data and the large one‐bond ^6,7^Li,^1^H and ^6,7^Li,^13^C NMR coupling constants found for the three‐atom H−Li−C chains. By contrast, the phenyl group in **4⋅LiC_6_H_5_** coordinates in an η^2^ fashion to the lithium atom through the *ipso* and one of the *ortho* carbon atoms.

## Graphical abstract

The coordination of monomeric LiCH_2_R (R=H, CH_3_) and LiC_6_H_5_ molecules to a Mo_2_ core is reported. The molecular structures of the new complexes were analyzed both in solution by NMR spectroscopic techniques and in the solid state and gas phase by X‐ray and computational studies.

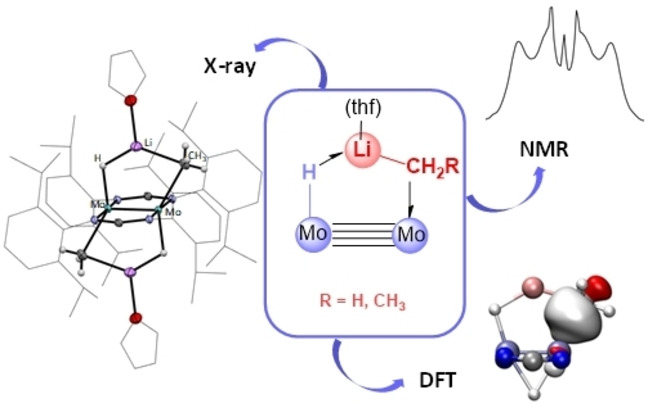

## Introduction

In the late 1960s, evidence derived from the systematic investigation of the “nickel effect” by Wilke and co‐workers provided the first hints on the coordination to transition metals of electropositive main‐group metal–hydrogen and ‐carbon bonds, E−X (X=H, C; E=Li, Mg, Al), with formation of multicenter bonds.^[1]^ At the outset, it was repeatedly observed that aluminum alkyls and hydrides stabilized solutions of extremely reactive Ni^0^ olefin complexes alike Ni(C_2_H_4_)_3_, although well‐defined products could not be isolated. Subsequent research from the group, including valuable contributions from Jonas and Pörshcke, allowed for the characterization by X‐ray diffraction of Ni^0^ olefin complexes seemingly incorporating coordinated Li−C, Mg−C and Al−H bonds.[^2^, ^3^, ^4^, ^5^] At that time, the nature of the Ni−X−E bridging bond was not ascertained, but nowadays it is widely accepted that the Ni(μ‐H)Al complex reported by Pörschke et al. in 1990,^[5]^ evidenced for the first time unsupported alane coordination.^[6]^

The study of intermolecular complexes of transition metals and main‐group metals like Mg, Al, Ga or Zn E−H bonds, has recently emerged as a principal focus of research, because these compounds constitute key intermediates in bond activation reactions.[^6^, ^7^] Information on analogous complexes of E−C bonds is, nevertheless, sparse, although a few compounds presumably containing coordinated E−C bonds have been reported.[^2^, ^3^, ^4^, ^8^, ^9^, ^10^, ^11^, ^12^, ^13^, ^14^, ^15^, ^16^, ^17^] It is well known that the κ^2^‐E,H coordination of H−H,^[18]^ B−H, C−H or Si−H bonds is a three‐center two‐electron (3c–2e) interaction that can be described with the aid of the Dewar–Chatt–Duncanson model.[^19^, ^20^, ^21^, ^22^] Using the half‐arrow symbology proposed by Green, Green and Parkin,^[22]^ the coordination of Li−C bonds to molybdenum discussed in this contribution will from now on be portrayed as Li−C⇀Mo. For the related E−H and E−C complexes of main‐group metals, increased ionic character for the M−X−E bridge bonding can be anticipated, given the increased difference in the Pauling electronegativity of the elements. For example, Δχ_p_=1.5 for a Li−C bond vs. 0.3 for C−H and Si−H bonds.^[23]^ As a consequence, wider bonding perspectives are foreseeable,[^6^, ^7^, ^24^] as already disclosed by Crimmin and co‐workers in an insightful analysis of the electronic interactions between Mg−H bonds and Group 10 metal complexes.^[24]^ Moreover, the coordination and electronic unsaturation of the electrophilic coordinated E atom will probably make mandatory that robust E−X bond coordination of simple hydrides,^[25]^ or alkyls like LiCH_3_, Mg(CH_3_)_2_ or Al(CH_3_)_3_, be supported by intramolecular electronic interactions with close donor atoms.[^6^, ^21^]

**Figure 1:**
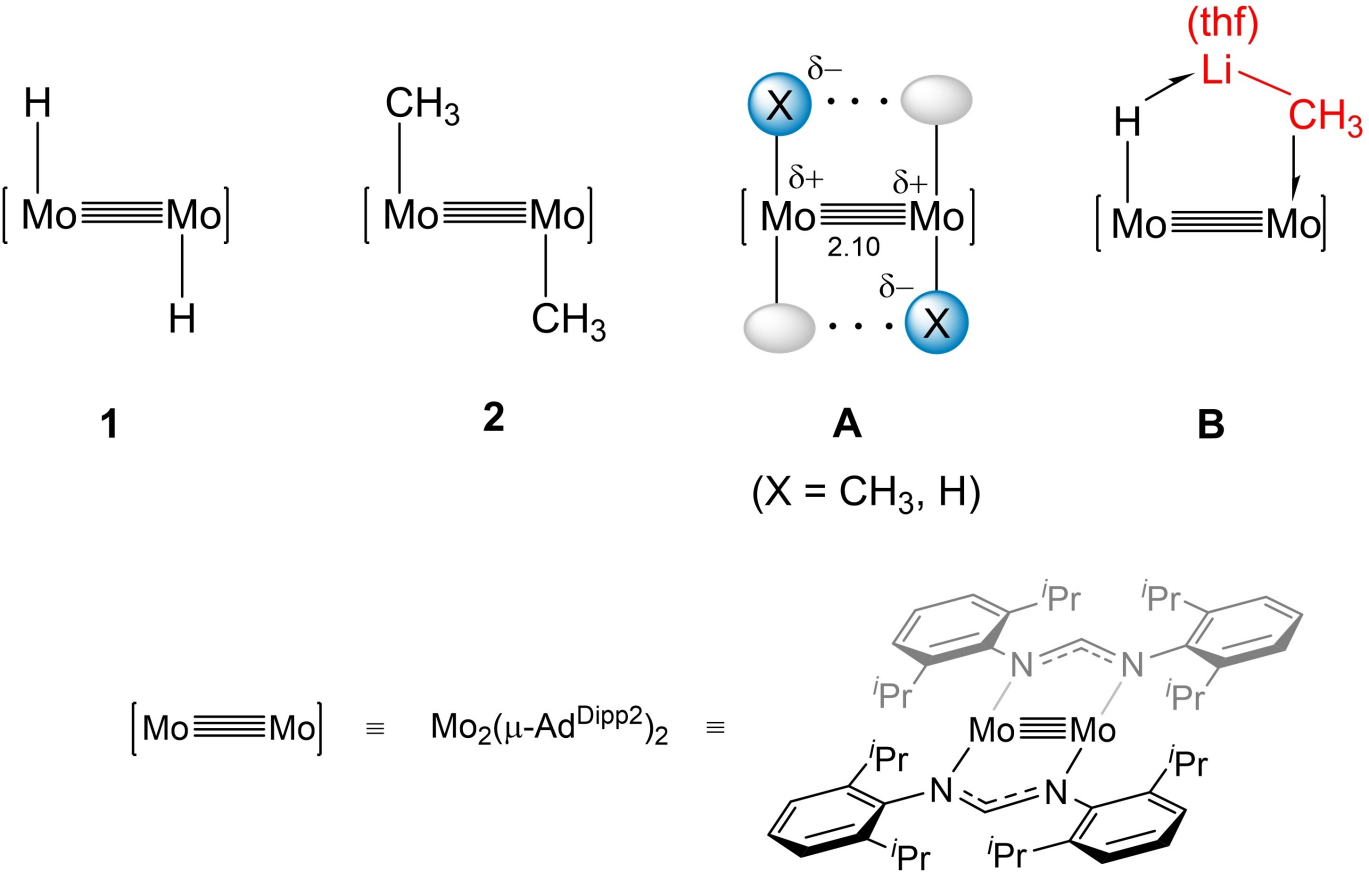
Simplified drawings of the structure of complexes [Mo_2_(X)_2_(μ‐Ad^Dipp2^)_2_] (X=H, **1**; CH_3_, **2**). Representation **A** highlights the bifunctional Lewis base/Lewis acid behavior, and potential cooperative effects between the Mo−X moieties (ellipsoids symbolize vacant coordination sites). **B** illustrates the multicenter structure resulting from the coordination of (thf)LiCH_3_ to a (H)Mo≣Mo unit. In this and following figures and schemes, [Mo≣Mo] represents the fragment [Mo_2_(μ‐Ad^Dipp2^)_2_].

On these grounds, we envisaged that the *trans*‐(X)Mo≣Mo(X) cores of the [Mo_2_(X)_2_(μ‐Ad^Dipp2^)_2_] complexes shown in Figure 1 (X=H, **1**; CH_3_, **2**; Ad^Dipp2^=HC(NDipp)_2_; Dipp=2,6‐^*i*^Pr_2_C_6_H_3_),[^26^, ^27^] could act as templates for stabilization of σ‐E−C and σ‐E−H interactions. Besides warranting mutual cooperative effects, the Mo(X) moieties of **1** and **2**, at a short separation of ca. 2.10 (structure **A** in Figure 1), are expected to exhibit bifunctional Lewis acid/Lewis base behavior, thanks to the strong nucleophilicity of the polar Mo^δ+^−X^δ−^ bond and the heightened electrophilic properties resulting from the *trans* empty coordination site and d valence orbitals. The rare simultaneity of these circumstances prompted us to study the reactivity of complexes **1** and **2** against diverse E−C and E−H bonds.

Here, we demonstrate that the Li−C bonds of simple, widely employed organolithium reagents such as LiCH_3_, LiCH_2_CH_3_ and LiC_6_H_5_, can bind to Mo≣Mo bonds forming stable molecular organometallic structures. Our results also prove beyond doubt that by forming strong Mo−H−Li−C−Mo multicenter bonds, the [Mo_2_(H)_2_(μ‐Ad^Dipp2^)_2_] scaffold provides protection to the above organolithium molecules from their natural tendency to aggregate through the formation of Li−C−Li bridges.[^28^, ^29^, ^30^, ^31^] Although LiC_6_H_5_ exists as a monomer in [Li(C_6_H_5_)(pmdta)]^[32]^ (pmdta=pentamethyldiethylenetriamine, NMe(CH_2_CH_2_NMe_2_)_2_), we are not aware of the existence of related monomeric complexes of LiCH_3_ and LiCH_2_CH_3_.^[33]^ We have also studied the reaction of the dimethyl complex **2⋅thf** ^[26]^ with LiAlH_4_ as a source of LiH. Contradicting our expectations, instead of forming the anticipated Mo−C−Li−H−Mo metallacyclic rings, LiH promoted Mo−CH_3_ to Mo−H bond metathesis^[34]^ and elimination of LiCH_3_, ultimately generating a hydride‐rich Mo_6_Li_9_H_18_ cluster, recently prepared by our group by a different procedure.^[35]^

## Results and Discussion

**Scheme 1:**
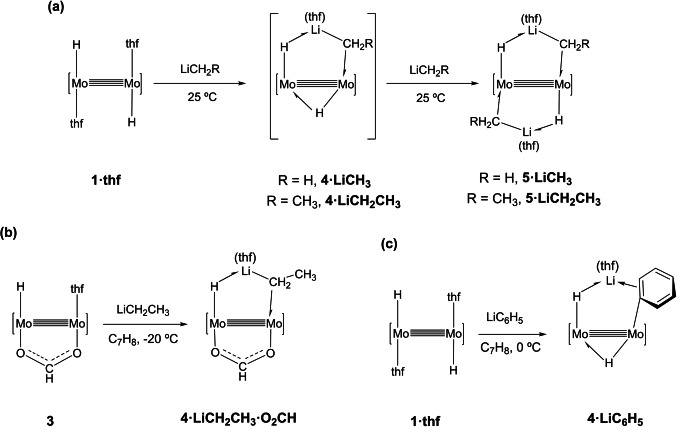
Reactions of complex **1⋅thf** with LiCH_3_ and LiCH_2_CH_3_ to generate intermediates **4⋅LiCH_3_** and **4⋅LiCH_2_CH_3_** and end‐products **5⋅LiCH_3_** and **5⋅LiCH_2_CH_3_** (a); b) and c) show the syntheses of complexes **4⋅LiCH_2_CH_3_⋅O_2_CH** and **4⋅LiC_6_H_5_**, respectively.

An instant color change from yellow‐orange to dark red was observed when solutions of LiCH_3_ and complex **1⋅thf** were mixed at room temperature in thf in a ca. 1 : 2 molar ratio. Following regular work‐up, a yellow crystalline solid was isolated and identified as complex **5⋅LiCH_3_**, incorporating two molecules of LiCH_3_ to the coordination sphere of the Mo≣Mo bond (Scheme 1a). Partial decomposition occurred^[33]^ as denoted by the formation of small quantities of LiAd^Dipp2^. ^1^H and ^7^Li NMR monitoring of the transformation utilizing 1 : 1 and 1 : 2 molar mixtures of reagents, revealed initial formation of a reactive intermediate **4⋅LiCH_3_**, that could not be isolated as a pure solid but was fully characterized by solution multinuclear NMR experiments and computationally confirmed as a minimum energy structure. In the presence of additional amounts of LiCH_3_, fast conversion to the bis(methyllithium) complex **5⋅LiCH_3_** took place.

LiCH_2_CH_3_ behaved similarly and formed an analogous complex **5⋅LiCH_2_CH_3_** through intermediate **4⋅LiCH_2_CH_3_** (Scheme 1a), that could not be isolated either as a pure crystalline solid. By contrast, the two bis(alkyllithium) complexes **5⋅LiCH_3_** and **5⋅LiCH_2_CH_3_**, were completely characterized by microanalytical, NMR and X‐ray techniques. As represented in Scheme 1a, the new complexes contain unprecedented H−Mo≣Mo−CH_2_(R)−Li metallacyclic moieties that may be viewed as σ‐Li−CH_2_R (R=H, CH_3_) complexes supported by an intramolecular electronic interaction with a vicinal, strongly nucleophilic Mo−H terminus. The formally monoanionic H−Li−CH_2_R bridging entities behave as three‐electron donor ligands so that each Mo center reaches a sixteen‐electron count.

The decreased stability of the mono(alkyllithium) adducts **4** relative to their bis(alkyllithium) counterparts **5**, can be associated with the insufficient steric protection provided by the bridging Mo−H−Mo hydride ligand. In accordance with this hypothesis, the monohydride complex [Mo(H)(μ‐Ad^Dipp2^)_2_(μ‐O_2_CH)(thf)] (**3**), that contains a coordinated bridging formate group opposite to the Mo−H site, permitted isolation of a stable mono(ethyllithium)‐formate adduct, **4⋅LiCH_2_CH_3_⋅O_2_CH** with the molecular complexity shown in Scheme 1b. At variance with this result, the analogous reaction of complex **3** and LiCH_3_ yielded an intractable mixture of products. Also in accordance with the above premise, the sterically more demanding phenyllithium provided a stable mono(organolithium) adduct, **4⋅LiC_6_H_5_**, with the structure depicted in Scheme 1c. Coordination of a second molecule of LiC_6_H_5_ did not prove feasible. In all likelihood this is due to steric hindrance, as demostrated recently during studies of the reactivity of complex **1⋅thf** toward classical donor ligands such as pyridines and tertiary phosphines.^[27]^

**Scheme 2:**
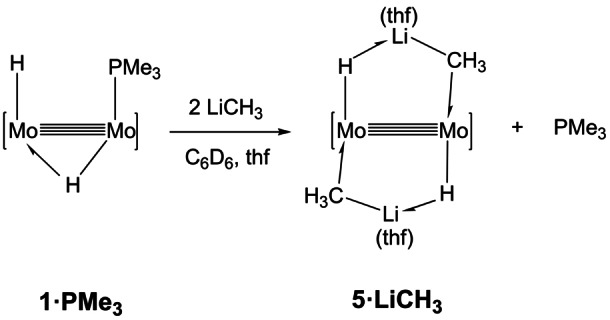
Displacement of coordinated PMe_3_ in **1⋅PMe_3_** by LiCH_3_ and formation of complex **5⋅LiCH_3_**. As explained in the text, LiCH_2_CH_3_ and LiC_6_H_5_ reacted similarly yielding corresponding complexes and free PMe_3_.

The Lewis base role of the Li−C bond toward complex **1⋅thf** represented in Scheme 1 encounters additional support in the experimental findings summarized in Scheme 2. With the objective of forming a purported complex {Mo_2_(H)[μ‐HLi(thf)CH_3_](μ‐Ad^Dipp2^)_2_(PMe_3_)}, i. e. the PMe_3_ adduct of **4⋅LiCH_3_**, by addition of a molecule of LiCH_3_ to the Mo(μ‐H)Mo moiety of compound **1⋅PMe_3_**, equimolar mixtures of the latter and methyllithium were dissolved in C_6_D_6_, in the presence of a few drops of added tetrahydrofuran. The target product was not detected by ^1^H, ^7^Li and ^31^P NMR studies, which on the contrary revealed the formation of **5⋅LiCH_3_**, accompanied by free PMe_3_ and unreacted **1⋅PMe_3_**. The use of 2 equivalents of LiCH_3_ yielded **5⋅LiCH_3_** quantitatively by NMR. Moreover, **1⋅PMe_3_** reacted similarly with LiCH_2_CH_3_ and LiC_6_H_5_, generating cleanly the corresponding complexes **5⋅LiCH_2_CH_3_** and **4⋅LiC_6_H_5_**.

**Scheme 3:**
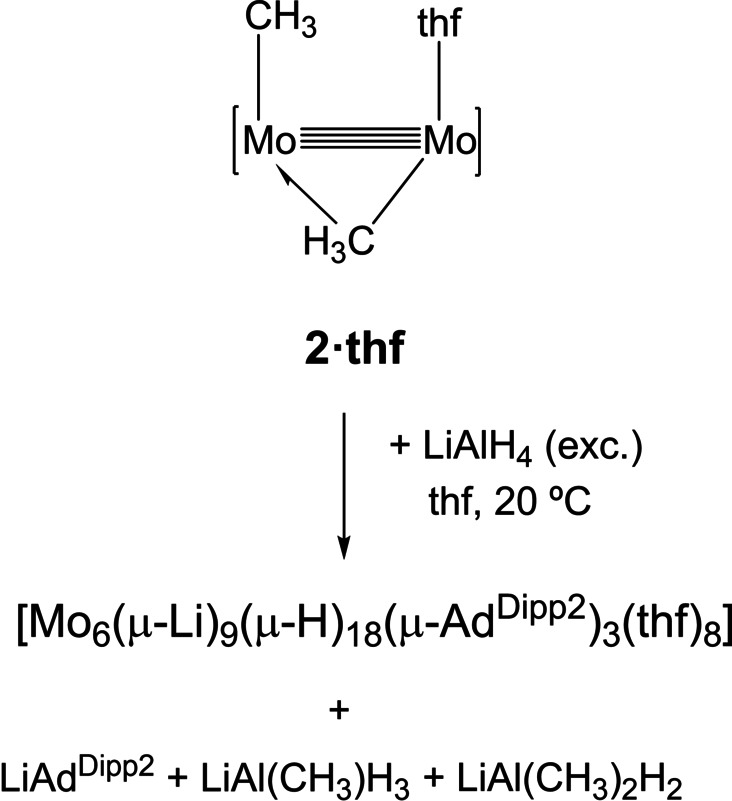
The reaction of the dimethyl complex **2⋅thf** with LiAlH_4_ as a source of LiH.

In marked contrast with the formation of the above organolithium adducts by the procedure disclosed in Scheme 1, the reaction of dimethyl complex **2⋅thf** with LiAlH_4_ as a source of LiH led, as shown in Scheme 3, to a recently characterized hydride‐rich Mo_6_Li_9_H_18_ cluster.^[35]^ Formation of LiAd^Dipp2^, LiAl(CH_3_)H_3_ and LiAl(CH_3_)_2_H_2_ ^[36]^ byproducts was inferred by ^1^H and ^7^Li NMR spectroscopy. The latter compounds are possibly Lewis adducts arising from the AlH_3_/LiCH_3_ system. They were generated independently from an equimolar mixture of LiAlH_4_ and LiCH_3_ for NMR identification purposes. It appears that in comparison to the Mo−H bonds of **1⋅thf**, the Mo−CH_3_ bonds of **2⋅thf** are not Lewis basic enough to sustain Li−H coordination at the proximal unsaturated molybdenum atom. Instead, LiH promotes Mo−CH_3_ to Mo−H bond metathesis with elimination of LiCH_3_.^[34]^ In agreement with this assumption, the low‐temperature (−20 °C) reaction of **2⋅thf** with 2 equivalents of LiAlH_4_ in tetrahydrofuran, gave initially the Mo_2_Li_2_H_4_ cluster {Mo_2_[μ‐HLi(thf)H]_2_(μ‐Ad^Dipp2^)_2_}, which is known to react with extra quantities of LiAlH_4_ to ultimately afford the cited Mo_6_Li_9_H_18_ cluster.^[35]^

All new complexes in Scheme 1 are very reactive toward traces of H_2_O and O_2_ and their manipulation requires use of Schlenk or glove‐box techniques. Under an atmosphere of Ar or N_2_, isolated complexes **4** and **5** exhibit fair solution stability at room temperature. This observation is truly remarkable, particularly in what concerns the methyllithium complex **5**. Although this is the least stable of the complexes isolated in this work, it features a half‐life of ca. 24 hours at 20 °C. For comparison, the recently characterized monomer [Li(CH_3_)(*κ* ^3^‐*N*,*N′*,*N*’’‐DETAN)] decomposes in ether and aromatic hydrocarbon solvents at −20 °C, thwarting structural characterization by NMR.^[33]^ The stability of the LiCH_2_CH_3_ complexes **4⋅LiCH_2_CH_3_⋅O_2_CH** and **5⋅LiCH_2_CH_3_** is additionally surprising considering the well‐known tendency of M−CH_2_CH_3_ complexes to undergo β‐H elimination.[^37^, ^38^, ^39^] Multiply bonded dimolybdenum complexes are no exception, as exemplified by Chisholm's triply bonded [Mo_2_(CH_2_CH_3_)_2_(NMe_2_)_4_],^[40]^ and also by the closely related Mo≣Mo compound [Mo_2_(CH_2_CH_3_)_2_(μ‐Ad^Dipp2^)_2_], that at room temperature undergoes β‐H elimination within minutes.^[41]^ We suggest that the reluctance of the above complexes to experience β‐H elimination is due to the difficulty encountered by the β hydrogen atoms to approach the Mo atom, as a result of the rigidity of the five‐membered H−Mo−Li−CH_2_(CH_3_)−Mo metallacycle. Though as solids the new compounds can be manipulated at room temperature, storage at −20 °C is advisable.

Reactivity studies, including the exchange of the coordinated LiR in compounds **4** and **5** by a different LiR’ reagent were carried out. As shown in Scheme S1, the transformations were, in general, complex and yielded a mixture of products comprising LiAd^Dipp2^. Similar complexity has been encountered for the recently reported LiCH_3_‐DETAN complex and other monomeric lithium organyls.^[33]^

IR spectroscopy was of little use for the identification of the bridging hydrides present in the Mo(μ‐H)Mo and H−Mo−Mo−C−Li moieties of the complexes (see the SI). By contrast, ^1^H, ^7^Li and ^13^C, 1D and 2D NMR experiments provided fundamental information for the unequivocal characterization of the solution structure of complexes of types **4** and **5**. X‐ray studies on single crystals of complexes **4⋅LiCH_2_CH_3_⋅O_2_CH**, **4⋅LiC_6_H_5_**, **5⋅LiCH_3_** and **5⋅LiCH_2_CH_3_** demonstrated that the NMR‐determined solution structures are maintained in the solid‐state. Besides, the structures of the above complexes as well as those of intermediates **4⋅LiCH_2_R** (R=H, CH_3_) have been computationally optimized in the gas phase and found to be energy minima in the potential energy surface.

**Figure 2:**
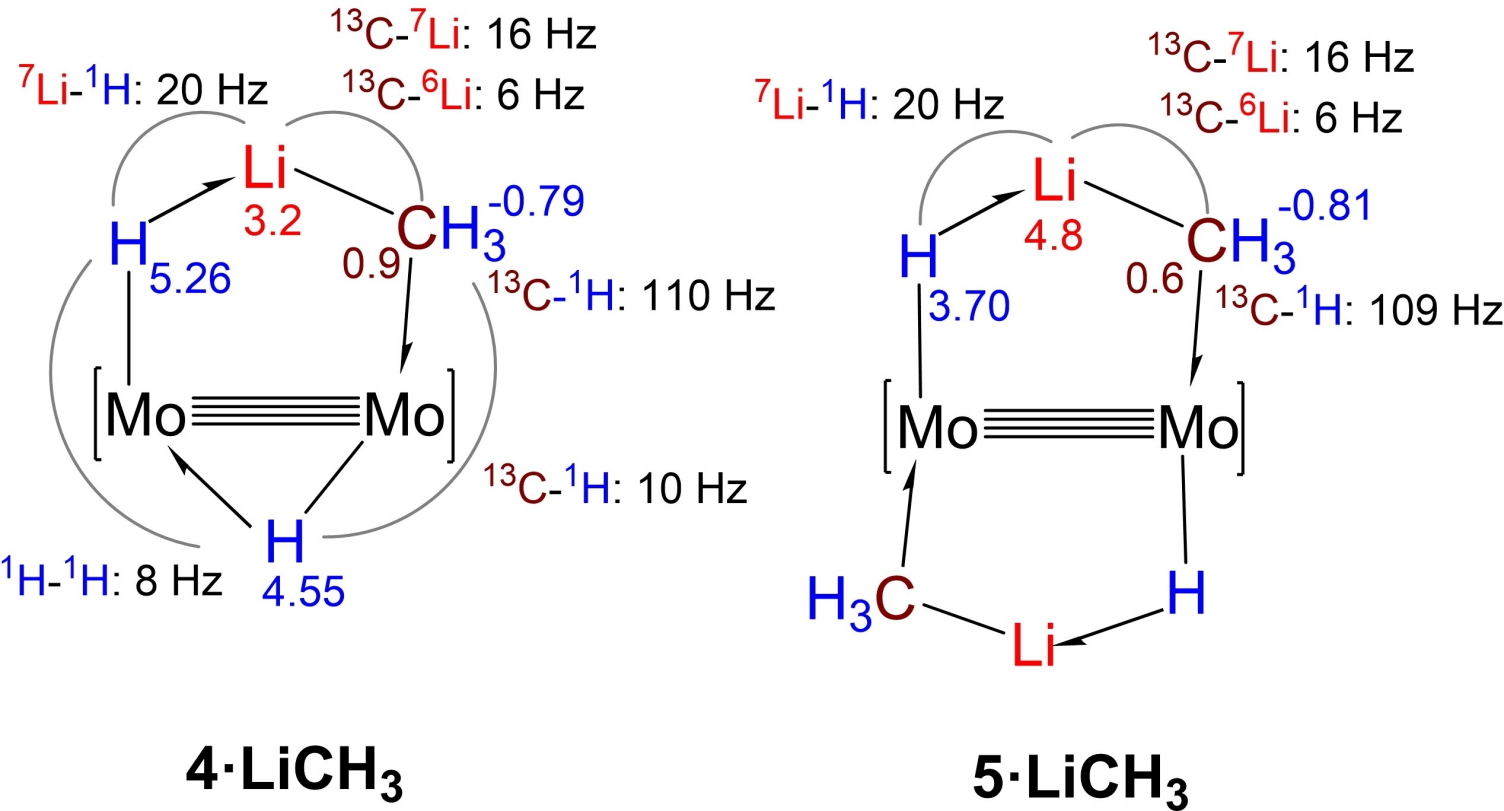
Relevant NMR chemical shifts (in ppm) and coupling constants (Hz) for the Mo−Mo bridging hydride and H−Li−CH_3_ ligands in complexes **4⋅LiCH_3_** (left) and **5⋅LiCH_3_** (right).

**Figure 3:**
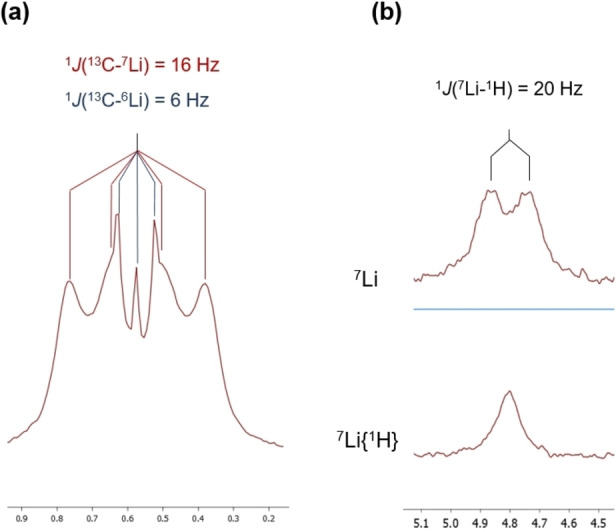
a) ^13^C{^1^H} DEPT‐135 NMR experiment for the bridging Mo−^13^CH_3_−Li methyl group in the ^13^C‐enriched complex **5⋅Li^13^CH_3_**; b) ^7^Li and ^7^Li{^1^H} NMR spectra of complex **5⋅LiCH_3_**.

The most meaningful NMR parameters obtained for the methyllithium complexes **4⋅LiCH_3_** and **5⋅LiCH_3_** are presented in Figure 2, while those pertaining the ethyl‐ and phenyllithium complexes are included in Figures S1 and S2 of the accompanying SI. To demonstrate unequivocally that scalar coupling between the ^6,7^Li and ^13^C nuclei is maintained within the H−Mo−Mo−C−Li metallacycles of complexes **4** and **5**, their ^13^C isotopologues were also investigated (99 % ^13^C enrichment). Leaving aside NMR signals due to the ancillary Ad^Dipp2^ ligands, for complex **4⋅LiCH_3_**, three ^1^H resonances are recorded with δ −0.79 (relative intensity 3 H), 4.55 (1 H) and 5.26 (1 H). The most shielded signal arises from the Li−CH_3_−Mo fragment whereas the other two are assigned to the Mo−H−Mo and Mo−H−Li bridges. For **5⋅LiCH_3_**, the Mo−CH_3_−Li signal does not change significantly (Figure 2) but the Mo−H−Li one moves to lower frequency and appears at 3.70 ppm. Somewhat reduced one‐bond ^13^C_sp3_‐^1^H coupling constants close to 110 Hz and relatively large ^13^C‐^6^Li (6 Hz) and ^13^C‐^7^Li (16 Hz) couplings can be measured in the spectra of the ^13^C enriched samples. In particular, the DEPT‐135 NMR experiment represented in Figure 3a for the ^13^CH_3_ signal of complex **5⋅Li^13^CH_3_** allows direct measurement of the above cited ^13^C–^6,7^Li coupling constants which, as expected, reflect very precisely the 2.64 quotient of the ^7^Li and ^6^Li gyromagnetic ratios.^[42]^ Besides, comparison of the ^7^Li and ^7^Li{^1^H} NMR spectra of the complexes (Figure 3b) leads to a one‐bond ^7^Li–^1^H coupling constant of approximately 20 Hz.

The metallacyclic and Mo−H−Mo moieties of the remaining complexes of type **4** and **5** have similar NMR properties (Figures S3–S5 in the Supporting Information). A detailed analysis of the NMR parameters leads to the following general considerations: i) Mo_2_Li(C)(H) metallacycles in complexes **4** and **5** exhibit unusually large ^1^ *J*(^7^Li,^1^H) coupling constants of ca. 20 Hz. Few one‐bond ^7^Li–^1^H couplings can be found in the literature and those reported group in the 6–15 Hz interval.[^35^, ^43^, ^44^, ^45^] Accordingly, the ca. 20 Hz values found in this work are the largest thus far measured and can be taken as indicative of a significant degree of covalency in the Mo−H−Li bridging bonds. ii) Comparatively large one‐bond ^13^C–^7^Li coupling constants of ca. 16 and 13 Hz have been respectively disclosed for the LiCH_3_ and LiCH_2_CH_3_ derivatives, whereas for **4⋅LiC_6_H_5_** the smaller 5 Hz value observed might be attributed to η^2^‐C_6_H_5_ coordination to lithium, as found in the solid‐state and computed structures (see below). For the LiCH_3_ and LiCH_2_CH_3_ complexes, the above coupling constants are close to those found for the corresponding [LiR]_4_ tetramers. Taken together, the large magnitudes of ^1^ *J*(^7^Li,^1^H) and ^1^ *J*(^13^C,^7^Li) reveal a substantial covalent contribution to the multicenter bond holding together the atoms of the five‐membered H−Mo−Mo−C−Li rings. Following Elschenbroich,^[46]^ the large and positive ^7^Li chemical shifts recorded for the new LiR complexes, particularly the 4.8 and 4.3 ppm values measured for **5⋅LiCH_3_** and **5⋅LiCH_2_CH_3_**, respectively, support also the proposed appreciable covalent character of the Li−H and Li−C bonds present in the new molecules reported.

**Figure 4:**
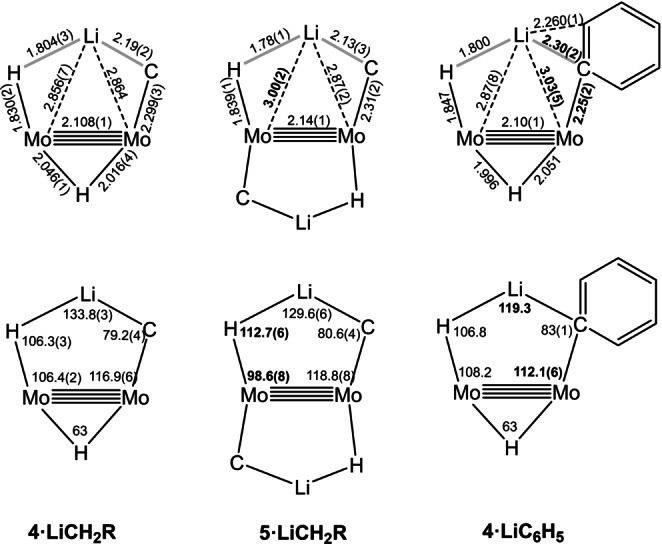
Average bond lengths and angles (in Å and °) for the Mo_2_LiHC metallacycles of studied complexes. The thick gray lines indicate distances significantly longer than the covalent radii sums. Numbers in boldface highlight the most relevant differences with the corresponding bonding parameters in **4⋅LiCH_2_R**. The values shown are averages of experimental and calculated values, except for those parameters involving the hydride atom close to the Li atom, for which only calculated data were used.

**Figure 5:**
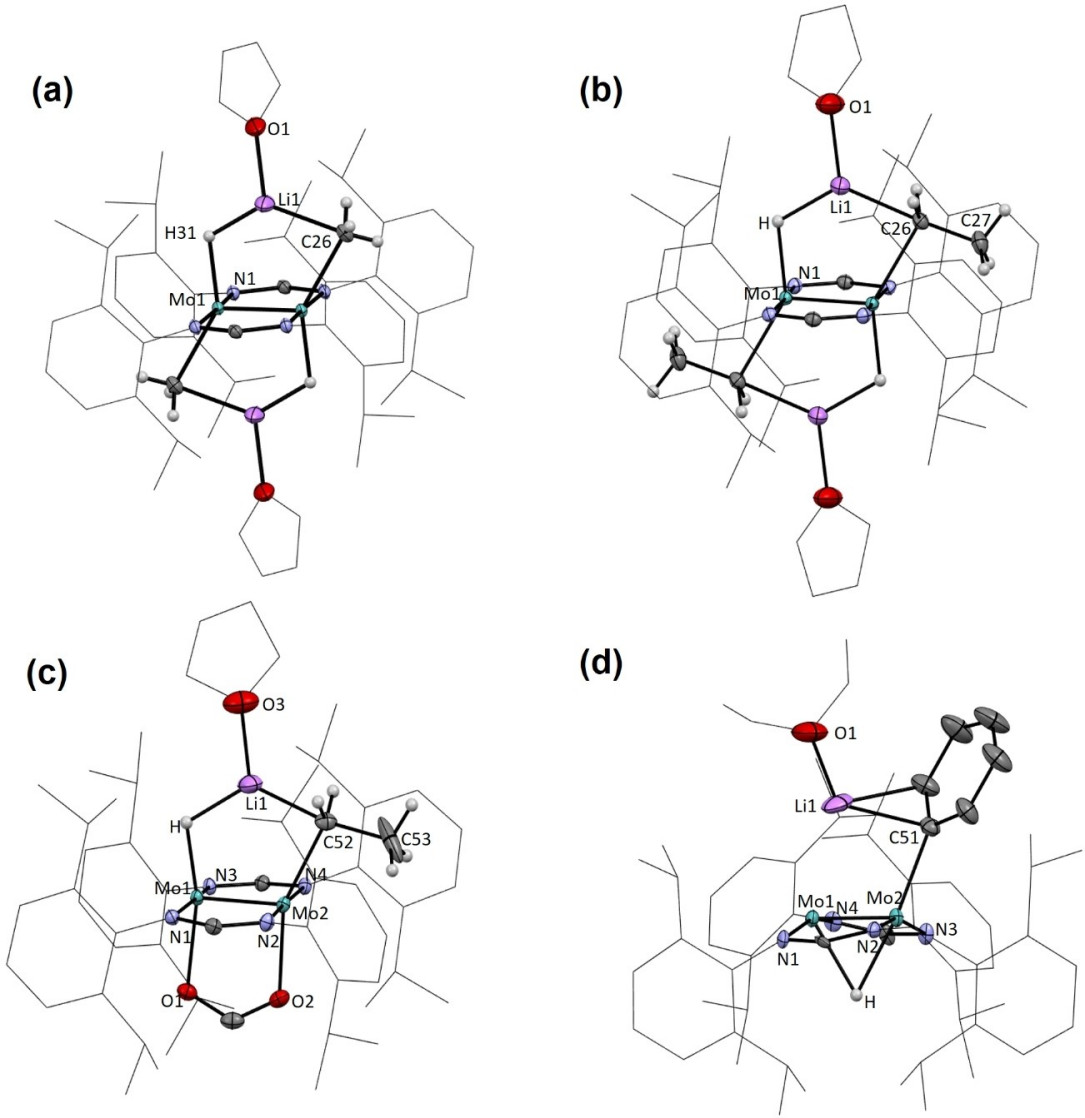
Solid‐state molecular structures of complexes **5⋅LiCH_3_** (a), **5⋅LiCH_2_CH_3_** (b), **4⋅LiCH_2_CH_3_⋅O_2_CH** (c) and **4⋅LiC_6_H_5_** (d) as determined by single‐crystal X‐ray crystallography.

In general, calculated bonding parameters are in good agreement with the values obtained from X‐ray data (±0.04 Å in bond lengths and ±3.0° in bond angles). Average bond lengths and angles for the Mo_2_Li(C)(H) metallacyclic units and Mo(μ‐H)Mo bridge bonds in the new complexes are summarized in Figure 4. There are, however, some exceptions that pertain mainly to H‐containing bonds and angles. Probably, they are due to well‐known difficulties in refining the position of hydrogen atoms in close vicinity to a heavy atom like molybdenum. Figure 5 contains representations of the molecular structures of the four isolated lithium hydrocarbyl complexes, as determined by X‐ray crystallography.

**Figure 6:**
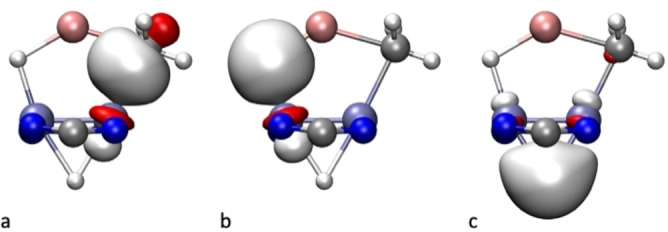
Occupied Natural Localized Molecular Orbitals of **4⋅LiCH_3_** that incorporate a) C−Mo and C−Li, b) H−Mo and H−Li, and c) Mo−(μ‐H)−Mo bonding character.

For the four alkyllithium complexes **4⋅LiCH_2_R** and **5⋅LiCH_2_R** (R=H, CH_3_), the results of an NBO analysis of the bonding within the Mo_2_Li(C)(H) metallacyclic units are similar. For reasons of brevity, we center the discussion on the mono methyllithium complex **4⋅LiCH_3_**. The four Mo−Mo bonding Natural Localized Molecular Orbitals (NLMO) that neatly represent the σ, π and δ components of that bond in **4⋅LiCH_3_** are similar to those found earlier for a related complex possessing a μ‐HLiH three‐atom chain, extending over a Mo≣Mo bond,^[35]^ and are, therefore, consistent with a quadruple bond. We have also been able to identify 2‐electron occupied NLMOs that display simultaneous C−Mo and C−Li (Figure 6a), or simultaneous H−Li and H−Mo bonding character (Figure 6b). Both these NLMOs are principally localized on the Mo−C and Mo−H bonds, respectively. As expected for significantly polarized Li−C and Li−H bonds, the contribution of Li to the bonding, through its partially occupied 2 s orbital, is quite low: 0.5 % and 2.4 %, respectively. On the other hand, the NLMO that hosts the two electrons responsible for the 3c–2e bond of the Mo−(μ‐H)−Mo moiety (Figure 6c) is nicely delocalized, although significantly polarized towards the H atom, in agreement with its hydridic character.

**Figure 7:**
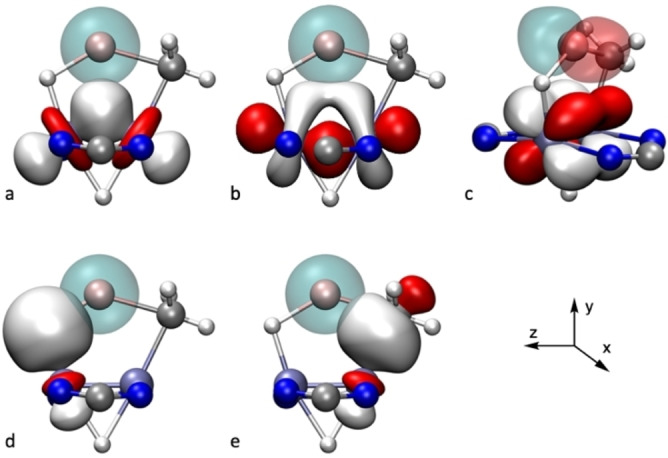
Donor (solid colors)–acceptor (transparent colors) interactions between natural orbitals of **4⋅LiCH_3_**: a) σ(Mo−Mo)→s(Li), b) π(Mo−Mo)→s(Li), c) δ(Mo−Mo)→p_z_(Li), d) σ(Mo−H)→s(Li), e) σ(Mo−C)→s(Li).

Since the contribution of Li to the bonding of the metallacyle is rather small, further stabilization of the structure can be envisaged on the basis of orbital donor‐acceptor interactions between a [H−Mo≣Mo−CH_3_]^−^ fragment (including the two bridging amidinate ligands) and a Li(thf)^+^ cation. These can be classified as (Mo−H)→Li, (Mo−C)→Li and (Mo≣Mo)→Li interactions, which may be obtained from the NBO analysis, and represent energetic stabilization contributions of 12.1, 5.5 and 4.3 kcal mol^−1^, respectively. Specifically, electron density donation from the Mo≣Mo bond to Li includes σ‐, π‐ and, to a lesser extent, δ‐bonding electron pairs (Figure 7a–c), while the principal acceptor orbital at Li has s character; donation to Li p‐orbitals can also be observed but with much lower stabilization energies. The donation from the Mo−C and Mo−H bonds mainly targets the s orbital of lithium as acceptor (Figure 7d, e); however, some weak delocalization can be found also onto the empty p‐orbitals of Li, not shown in Figure 7.

**Figure 8:**
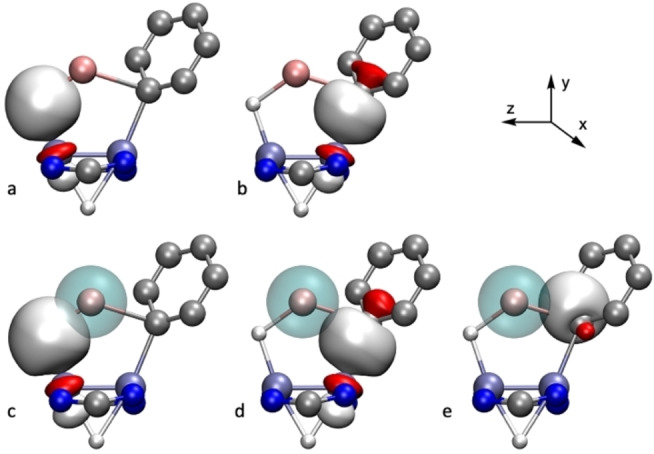
Natural Localized Molecular Orbitals with a) Mo−H−Li, b) Mo−C−Li and their corresponding Natural Orbital donor/acceptor interactions (c, d, e) found in complex **4⋅LiC_6_H_5_**.

The bonding in the phenyllithium complex **4⋅LiC_6_H_5_** is different because of the different electronic structure of the phenyl ring with respect to the methyl and ethyl groups. The NLMO analysis of this compound reveals that the bonding of the metallacycle has many similarities with that shown by the previous complexes, implying a quadruple Mo≣Mo bond, and 2‐electron natural orbitals with mainly Mo−H and Mo−C nature. Again, the contribution of Li to these orbitals is very low, with amounts of 1.9 % and 0.5 % to the Mo−H−Li and Mo−C−Li bonds, respectively (Figure 8a, b). As before, the donor/acceptor interaction regarding the Li atom has a significant contribution to the bonding. First, Mo−H⇀Li and Mo−C⇀Li donor‐acceptor interactions like those found for **4⋅LiCH_3_** can be found (Figure 8c, d). The Mo−Mo bonds behave in the same way as for **4⋅LiCH_3_**. Interestingly, this compound displays a novel donor/acceptor stabilizing interaction between a C_ipso_−C_ortho_ σ bond and the 2 s orbital of Li (σ(C−C)→Li, Figure 8e), this η^2^‐coordination may be at the basis of a 10° tilt of the phenyl ring coordinated to the Mo atom. The stabilization energies associated to donor‐acceptor interactions from the Mo−H, Mo−C, Mo−Mo and C−C bonds to Li are of 10.9, 4.9, 2.9 and 1.8 kcal mol^−1^, respectively, and mainly target the 2 s orbital of Li.

These dissimilar electronic properties are nicely reflected in the experimental and computed bonding parameters. Thus, the data collected in Figure 4 reveals that while the Mo−H−Li portion of **4⋅LiC_6_H_5_** matches closely the analogous fragments in the **4⋅LiCH_2_R** counterparts, important modifications are apparent among the Li−C−Mo sections of these molecules.

The calculated and experimental Mo−Mo bond lengths in the presently studied complexes are in the range 2.087–2.151 Å, with an average of 2.12(2) Å. These values are similar to those reported in the literature for related complexes containing eclipsed Mo_2_L_8_ frameworks made up of square planar MoL_4_ units, and are consistent with fourfold bonding between the Mo atoms.^[47]^ Given the existence of a multiple Mo−Mo bond in close proximity to a Li atom, to appraise homo‐ and hetero‐metallic bonding, Cotton's notion of formal shortness ratio (FSR) has been used.^[47]^ Other researchers have recently used this concept.[^7^, ^48^, ^49^] In our case (see Section 4 of the Supporting Information for details), the covalent radii of the atoms^[50]^ have been employed because Pauling's radii for d block elements often lead to single‐bond lengths smaller than experimentally determined bond lengths.[^50^, ^51^] An average FSR value of ca. 0.69 has been obtained for the Mo−Mo distances in the new complexes, while Mo^C−Li^ and Mo^H−Li^ distances have FSRs of 1.02 and 1.05, respectively. Comparing Mo−Mo bond lengths in the mono‐ and the bis‐alkyllithium complexes, **4⋅LiR** (R=CH_3_, CH_2_CH_3_ and C_6_H_5_) and **5⋅LiCH_2_R** (R=H, CH_3_), their averages increase upon incorporation of a second molecule of lithium alkyl (Scheme 1a) from 2.108(1) to 2.148(1) in our calculations, and from 2.087(1) in **4⋅LiC_6_H_5_** to 2.127(4) Å in **5⋅LiCH_2_R** in the X‐ray structures. In the formate complex **4⋅LiCH_2_CH_3_⋅O_2_CH**, that contains one molecule of LiCH_2_CH_3_, the Mo−Mo bond length is 2.1066(4) Å. In other words, the second lithium alkyl group induces a ca. 0.04 Å lengthening of the Mo−Mo bond (or an increase of 0.01 in the FSR). This bond‐lengthening effect of the second alkylation step should be attributed to the donation from the Mo−Mo bond to the additional Li atom, identical to the one discussed above. The Mo−H−Li−C−Mo rings can be seen as heterometallic Mo_2_Li triangles whose two Mo−Li edges are bridged by a hydrogen atom and the alkyl group. The Li−Mo distances are on average 2.92(7) Å, somewhat above the 2.82 Å sum of the covalent radii of the atoms,^[50]^ but yet indicating the existence of heterometallic bonding interactions, as discussed earlier.

To close the discussion on the structural properties of the new complexes, some comments on their Mo−H, Li−H, Mo−C and Li−C bonds seem appropriate. The distances between molybdenum and the hydride bridging a Mo−Li bond are in the range 1.71–1.85 Å, longer than the 1.60 Å value reported for the molecule of LiH in the gas phase,^[52]^ but appreciably shorter than the 2.04 Å separation in the solid‐state structure of LiH.^[25]^

Regarding the Mo−C bonds, the average of experimental Mo−alkyl bond lengths of 2.29(2) Å is comparable to those found for other Mo−CH_3_−Li units (2.25–2.3 Å).[^53^, ^54^] The bridging function of the alkyl groups becomes apparent from the comparison of the cited Mo−C distances of ca. 2.29 Å in **5⋅LiCH_3_** and **5⋅LiCH_2_CH_3_** with that of 2.154(2) Å ascertained for the terminal Mo−CH_2_CH_3_ bonds in [Mo_2_(CH_2_CH_3_)_2_(μ‐Ad^Dipp2^)_2_].^[41]^ With reference to the Mo_2_Li skeleton of the Mo_2_Li(C)(H) rings, the orientation of the alkyl groups in complexes **4⋅LiCH_2_R** and **5⋅LiCH_2_R** (R=H, CH_3_) relative to the pertinent Mo−Li edge of the triangle is such that the sp^3^ hybrid orbital available for bonding to the Mo−Li pair (represented by arrows in Figure S6a in the SI) is directed to that bond but much closer to the molybdenum than to the lithium atom. This fact can be attributed to the higher covalent character of the Mo−C bond compared to the Li−C bond. A comparison of the differences of the bond lengths with the corresponding covalent radii sums^[50]^ seems to confirm this interpretation (Figure S6b): while the average of experimental and calculated Mo−C distances is practically identical to the atomic radii sum, the C−Li average is slightly 0.12(9) Å longer than expected. The trend is still more marked for the bridging hydride, thus supporting a description of the ring as formed by a covalently linked H−Mo−Mo−C group that is σ‐bond coordinated to the lithium atom through the Mo−H and Mo−C bonds. Calculated Mayer bond orders of about 0.60, 0.10 and 0.13 for the Mo−C, Li−C and Li−H bonds, respectively (see Table S1), also attest to the significantly larger covalent nature of the Mo−C bond, as could be expected from electronegativity differences. But it is also evident that, similarly to the recently described Mo_2_Li(H)_2_ rings,^[35]^ there is a non‐negligible covalent contribution to Li−C and Li−H bonding. This is in accordance with the large ^6,7^Li−^1^H and ^6,7^Li−^13^C solution NMR coupling constants found for these complexes, as discussed in the preceding section. In particular, the ^1^ *J*(^7^Li,^13^C) NMR coupling constants determined for the LiCH_3_ complexes (Figure 2) are not dissimilar from the value reported for free methyllithium. The average Li−C distance of 2.15(3) Å in the methyllithium and ethyllithium complexes, while understandably longer than in the free molecules of LiCH_3_ (1.959 Å),[^55^, ^56^] are discernibly shorter than the 2.24(1) Å average value found for [Li(CH_3_)(thf)]_4_ ^[57]^ and in [LiCH_2_CH_3_]_4_, where the average Li−C separation is 2.27 Å,^[58]^ in agreement with the presence in the structurally characterized complexes **5⋅LiCH_3_** and **5⋅LiCH_2_CH_3_** of monomeric molecules of the lithium alkyls (Figures 4 and 5). With reference to the former, it is worth remarking that in the solid state the Li−C bonds have a length of about 2.16 Å, only marginally longer than the ca. 2.10 Å found for the recently reported monomeric methyllithium complex [Li(CH_3_)(DETAN)].^[33]^ It is also of note that in both the experimental (2.04(2) Å) and computed (2.17 Å) structures for **5⋅LiCH_3_** there is one agostic closed‐shell Li⋅⋅⋅H contact.[^59^, ^60^] Two such interactions have been unveiled computationally and experimentally for complex **5⋅LiCH_2_CH_3_**.

In the phenyllithium complex **4⋅LiC_6_H_5_**, the Li−C_*ipso*_ distance (experimental and calculated values of 2.28 and 2.33 Å, respectively) is much longer than in the already discussed alkyl analogs and ca. 0.11 Å longer than the corresponding distance in the monomeric phenyllithium complex [Li(C_6_H_5_)(pmdta)],^[32]^ though still shorter than the average Li−C contact cf. 2.33 Å reported for [Li(C_6_H_5_)(OEt)_2_]_4_.^[61]^ All these data are consistent with the already advanced conclusion that the phenyl ring is bonded to Li via its π electrons, which implies perforce a longer distance to the carbon atom than when σ‐bonded.^[62]^ The dissimilar topology of the phenyllithium bonding also accounts for the important differences observed in the H−Li−C and Li−C−Mo bond angles (Figure 4). In summary, the most remarkable differences with the lithium alkyl complexes are consistent with a change from a three‐center two‐electron Mo−C−Li bond in the alkyl complexes to an aryl σ‐coordinated to a Mo atom and η^2^(π)‐coordinated to the Li atom in **4⋅LiC_6_H_5_**.

## Conclusion

This work demonstrates that monomeric molecules of LiCH_3_, LiCH_2_CH_3_ and LiC_6_H_5_, can coordinate to Mo≣Mo bonds forming stable molecular H−Mo−Mo−C−Li frameworks, in which the high Lewis acidity of the lithium atom is compensated by a strong electronic interaction with an adjacent, polar Mo^δ+^−H ^δ−^ bond. The bonding within the five‐member H−Mo−Mo−C−Li rings in the LiCH_3_ and LiCH_2_CH_3_ complexes is best described as a covalently linked H−Mo≣Mo−C group σ‐bonded to the lithium atom through its Mo−H and Mo−C termini, supplemented by direct electron donation from the Mo≣Mo bond to lithium, mostly through its π and σ components, with a smaller contribution of the δ‐bonding electron pair.[^35^, ^63^] In the lithium aryl complex **4⋅LiC_6_H_5_**, the bonding mechanism is neatly different. The phenyl group is also bonded to a molybdenum atom, but a C_*ipso*_−C bond is η^2^‐coordinated to lithium, rather than the characteristic Mo−C σ‐bond coordination found in the alkyllithium complexes. The obtained NMR, X‐ray and computational data support the notion that, although mainly ionic, the new three‐atom hydrido‐alkyl (or ‐aryl) lithiate ligands, μ‐H−Li−C, possess a non‐negligible covalent character as a result of substantial orbital overlap and electron density sharing. When part of the robust Mo_2_Li(C)(H) cluster units, the monomeric molecules of the organolithium reagents show no tendency to aggregate through the formation of Li−C−Li bridges.

## Supporting Information

Experimental details for the preparation of the new complexes, NMR spectra, X‐ray crystallography^[64]^ and computational details, and atomic coordinates for the optimized geometries of the compounds.

## Conflict of interest

The authors declare no conflict of interest.

1

## Supporting information

As a service to our authors and readers, this journal provides supporting information supplied by the authors. Such materials are peer reviewed and may be re‐organized for online delivery, but are not copy‐edited or typeset. Technical support issues arising from supporting information (other than missing files) should be addressed to the authors.

Supporting Information

## Data Availability

The data that support the findings of this study are available in the supplementary material of this article.
