# Supplementary material for: Supported σ‐Complexes of Li−C Bonds from Coordination of Monomeric Molecules of LiCH3, LiCH2CH3 and LiC6H5 to Mo≣Mo Bonds
Source: Angew Chem Int Ed Engl. 2022 Jan 11;61(8):e202116009. doi: 10.1002/anie.202116009 (PMC9303556; doi:10.1002/anie.202116009)
Supplement: Supplementary file 1 — Supporting Information [file ANIE-61-0-s001.pdf]

Angewandte Chemie
Eine Zeitschrift der Gesellschaft Deutscher Chemiker

Supporting Information

Supported σ-Complexes of Li–C Bonds from Coordination of Monomeric Molecules of $LiCH_3$, $LiCH_2CH_3$ and LiC_6H_5 to Mo≣Mo Bonds

M. Pérez-Jiménez, J. Campos, J. Jover, S. Álvarez, E. Carmona**

SUPPORTING INFORMATION

pages

1. Figures S1-S6 1-4
2. Scheme S1 5
3. Table S1 - Calculated geometric parameters of the Mo_2C-Li-H ring, NBO orbital interaction energies, and Mayer bond orders 6
4. Assessing intermetallic bonding interactions in supported Li–C complexes of Mo≣Mo bonds and related complexes. Figures S7-S11 7-12
5. X-ray structural characterization of new compounds 13-15
6. Synthesis and characterization of new complexes 16-27
7. NMR spectra of new complexes 28-45
8. IR spectra of complexes **5·$LiCH_3$**, **5·$LiCH_2CH_3$** and **4·LiC_6H_5** 46-47
9. Computational details. Cartesian coordinates of computed complexes in xyz format 48-55
10. References 56-58

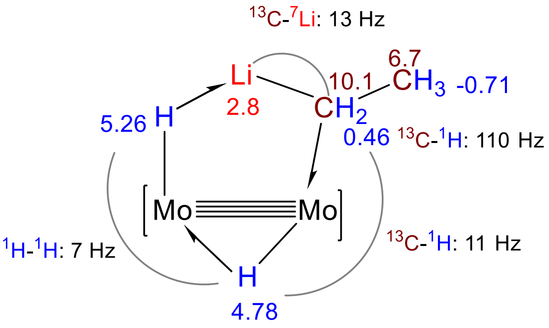

4·LiCH$_2$CH$_3$

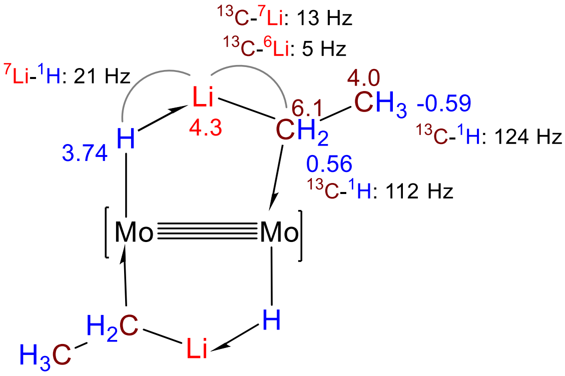

5·LiCH$_2$CH$_3$

Figure S1. Relevant NMR chemical shifts (in ppm) and coupling constants (Hz) for the Mo–Mo bridging hydride and H–Li–C ligands in complexes **4·LiCH$_2$CH$_3$** (left) and **5·LiCH$_2$CH$_3$** (right).

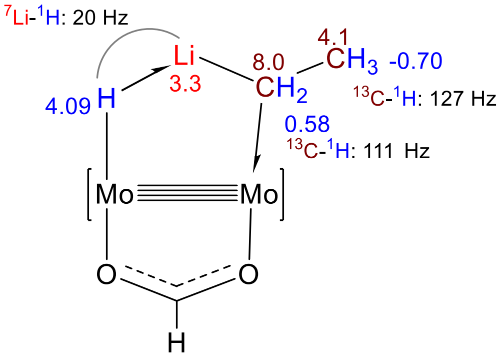

4·LiCH$_2$CH$_3$·O$_2$CH

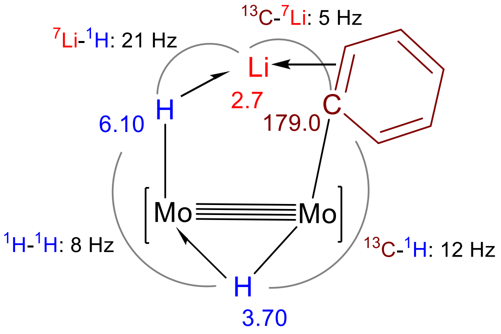

4·LiC$_6$H$_5$

Figure S2. Relevant NMR chemical shifts (in ppm) and coupling constants (Hz) for the Mo–Mo bridging hydride and H–Li–C ligands in complexes **4·LiCH$_2$CH$_3$·O$_2$CH** (left) and **4·LiC$_6$H$_5$** (right).

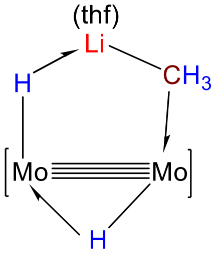

4·LiCH$_3$

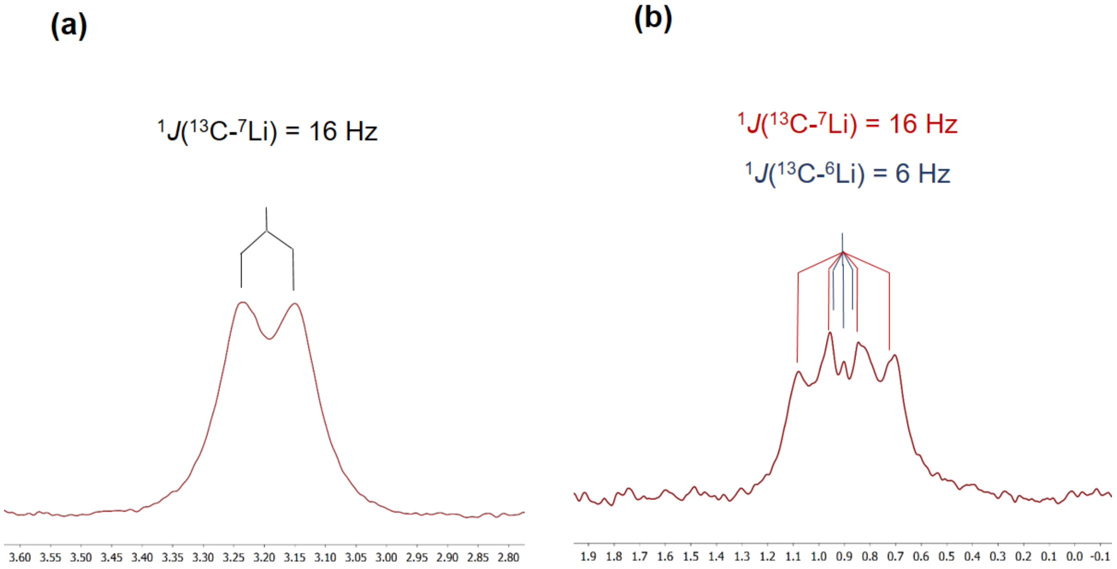

Figure S3. ^{7}Li{^{1}H} (**a**) and ^{13}C{^{1}H} DEPT-135 NMR (**b**) resonance of the Mo and Li bridging $^{13}CH_3$ group, of labelled complex **4·Li^{13}CH$_3$** showing one-bond ^{13}C-^{6}Li and ^{13}C-^{7}Li couplings.

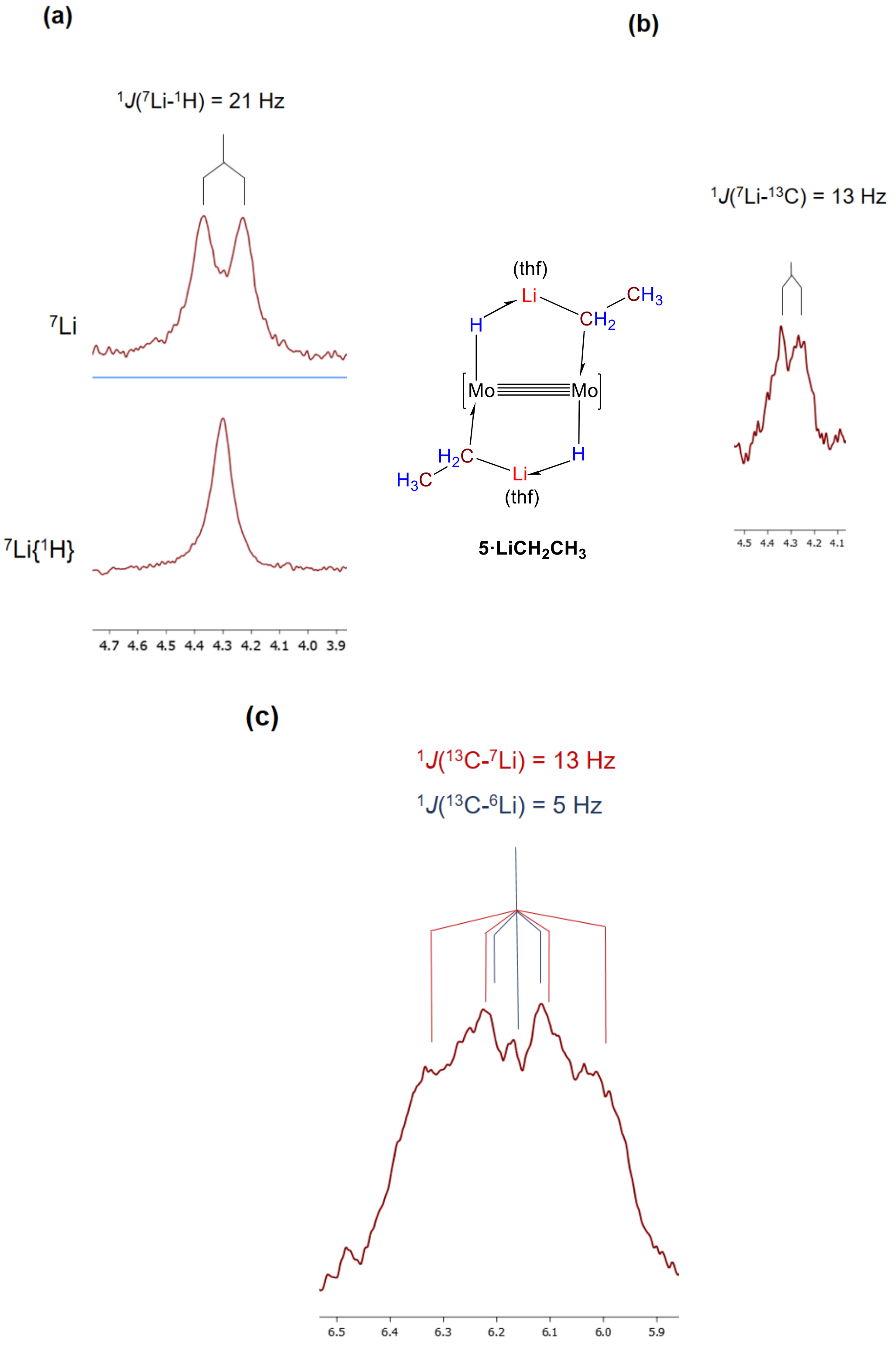

Figure S4. a) ^{7}Li and $^{7}Li\{^{1}H\}$ NMR spectra of complex **5·$LiCH_2CH_3$**; **b**) $^{7}Li\{^{1}H\}$ NMR spectrum of complex **5·$Li^{13}CH_2CH_3$**; **c**) $^{1}J(^{13}C,^{6}Li)$ and $^{1}J(^{13}C,^{7}Li)$ couplings disclosed by the $^{13}C\{^{1}H\}$ DEPT-135 NMR experiment for the bridging Mo–$^{13}CH_2CH_3$–Li labelled methylene group.

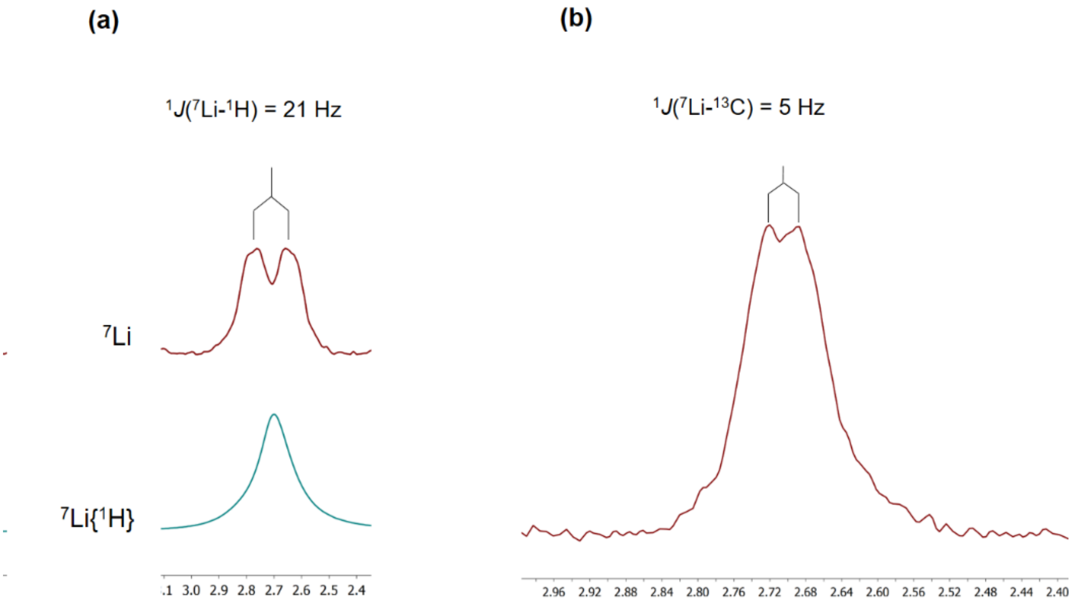

Figure S5. a) ^{7}Li and ^{7}Li{^{1}H} NMR spectra of complex **4·LiC$_6$H$_5$**; **b**) ^{7}Li{^{1}H} NMR spectrum of complex **4·Li^{13}C$_6$H$_5$**.

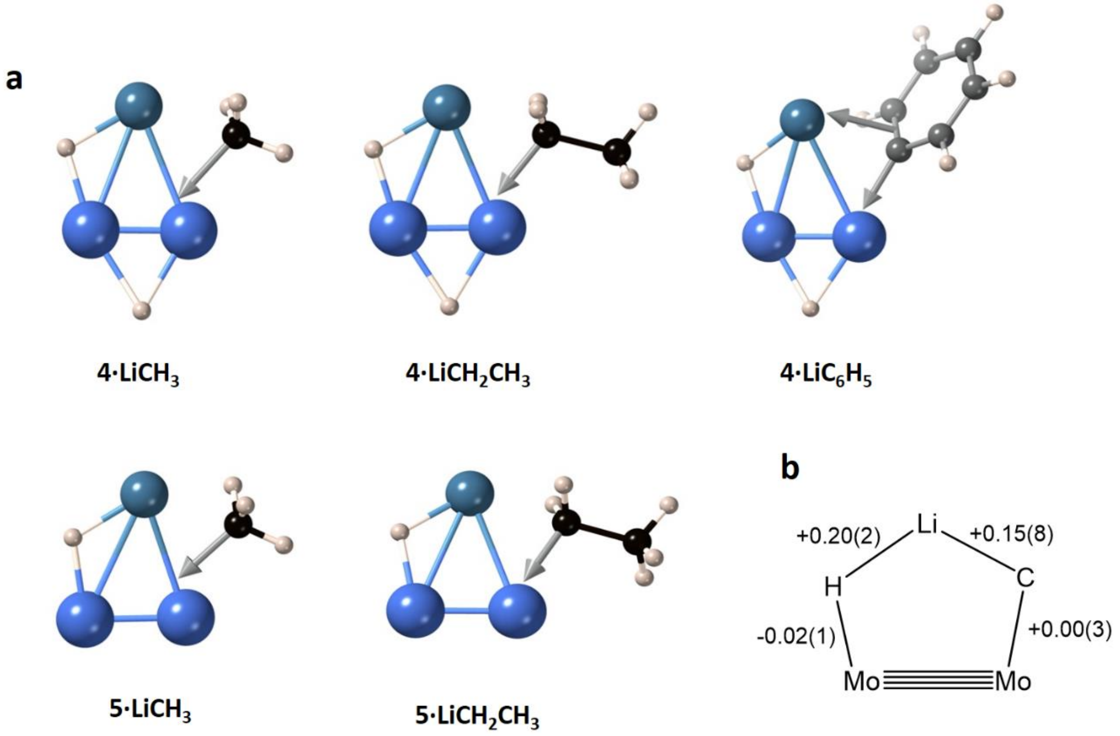

Figure S6. (**a**) Orientation of the R groups in **4·LiCH$_2$R** and **5·LiCH$_2$R** complexes (R = H, CH$_3$) and **4·LiC$_6$H$_5$** relative to a MoLi atom pair. The arrows indicate the approximate direction of the sp^3 orbital at the alkyl C atom and of the phenyl σ and π orbitals. (**b**) Differences between the interatomic distances and the sums of the covalent radii (in Å, standard deviations in parentheses).

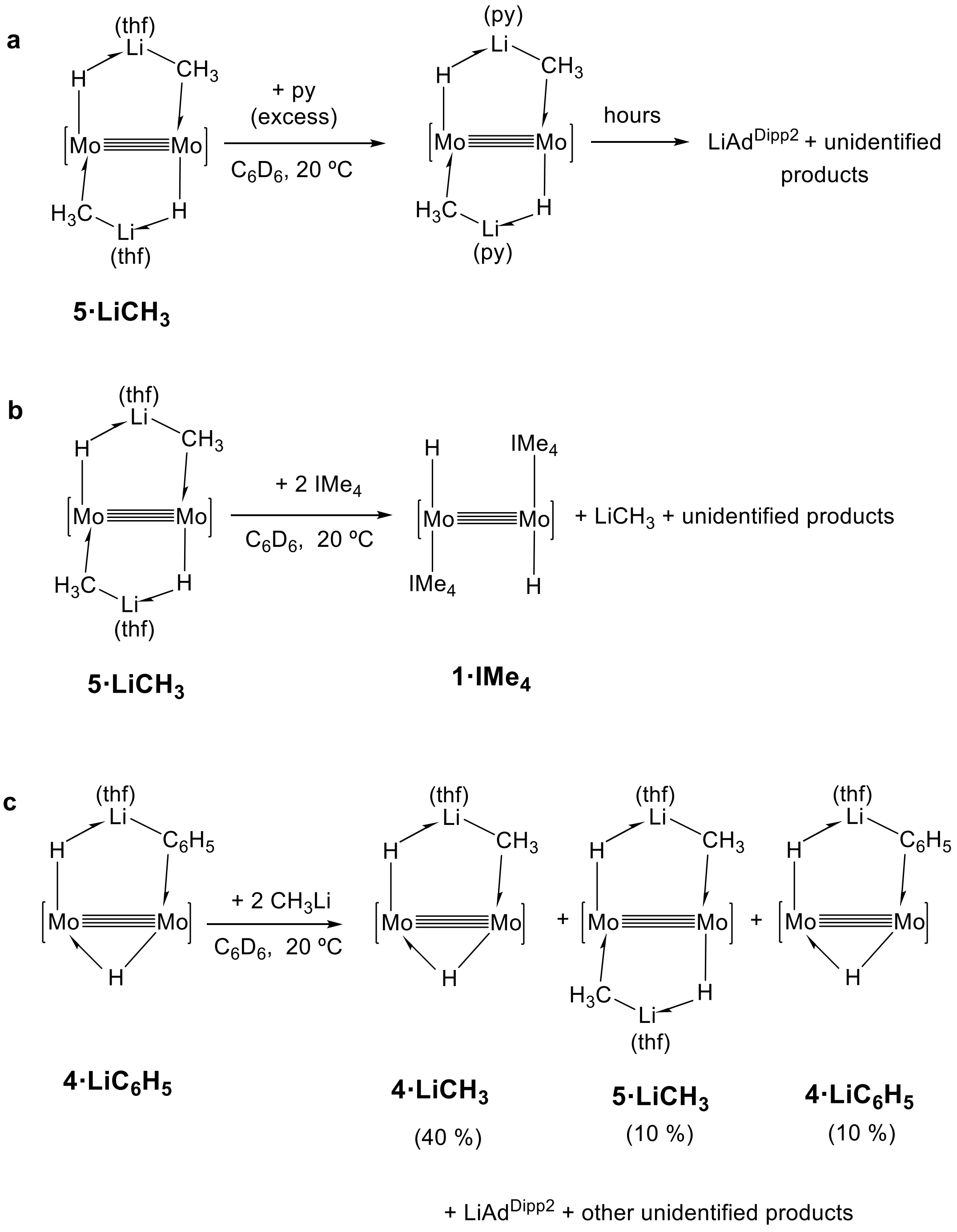

Scheme S1. The reactions of complex **5·LiCH₃** with pyridine (**a**) and IMe₄ (**b**), and of complex **4·LiC₆H₅** with LiCH₃(**c**). The product percentages correspond to NMR spectroscopic yields.

Table S1. Calculated geometric parameters of the Mo_2C-Li-H ring, NBO orbital interaction energies, and Mayer bond orders.

	Distances (Å)			Interaction energies (kcal/mol)					Mayer bond orders			
Cpd.	Mo–Mo	Mo–C	Li–C	Mo–Mo→Li	Mo–H →Li	Mo–C →Li	(C–C) →Li	Total	Mo–Mo	Mo–C	Li–C	Li–H
**4·LiEt·**	2.133	2.282	2.150									
**HCOO**	*2.107*	*2.194*	*1.997*						3.413	0.608	0.098	0.136
**4·LiMe**	2.107	2.297	2.179	4.3	12.1	5.5		22.9	3.615	0.618	0.089	0.135
**4·LiEt**	2.109	2.301	2.202	3.9	10.8	4.3		19.0	3.606	0.604	0.088	0.131
**5·LiMe**	2.144	2.321	2.128	1.8	8.5	3.3		13.6	3.400	0.605	0.098	0.128
	2.123	*2.292*	*2.125*									
**5·LiEt**	2.151	2.332	2.129	1.5	8.6	2.7		12.8	3.383	0.585	0.101	0.131
	2.129	*2.332*	*2.151*									
**4·LiPh**	2.108	2.264	2.328	2.9	10.9	4.9	1.8	18.7	3.584	0.627	0.061	0.132
			2.408		(2.260 centroid)						0.031	
	2.108	*2.264*	*2.282*									
			2.453									

In italics, experimental data

4. Assessing intermetallic bonding interactions in supported σ-Li–C complexes of Mo ≣Mo bonds and related complexes

As noted in the Results and Discussion section, X-ray crystallography continues to be the primary tool for defining intermetallic bonding. Nonetheless, interpreting metal-metal distances in terms of bonding is not a simple task, because absolute magnitudes of intermetal E···M distances cannot be compared. To circumvent this difficulty, tabulated covalent radii[1,2] and Pauling single-bond metallic radii[3] of atoms can be employed as a reference.

During studies on supershort multiple metal-metal bonds,[4] Cotton and colleagues defined the parameter "formal shortness ratio", FSR, for a bond A–B as follows:

$$FSR_{AB} = \frac{D\ (A - B)}{R_1^A + R_1^B}$$

Thus, FSR is the quotient of the experimental intermetal separation to the expected single-bond length for the bond in question (**s-b.l.** from this point onwards). To estimate the latter, Cotton used the set of Pauling's single-bond metallic radii, R_1 or r_m, that can be found in Table 11-1 (page 403) of Pauling's book *The Nature of the Chemical Bond*.[3] The applicability of the approximation finds strong support in the fact that regardless of the meaning of the absolute values of R_1, the collection affords a correct measure of the relative size of the atoms.[3]

Recently, other researchers have applied the FSR criterium to interpret metal-metal bonding in different intermetallic systems.[5-7] Thus, from X-ray studies on M(η^2-E–H) complexes, where E is predominantly Mg, Zn or Al, Buttler and Crimmin concluded that the magnitude of FSR can be used to distinguish between the above σ complex formulation and the isomeric M(H)(E) product resulting from oxidative addition. σ-Complex entities possess FSR values slightly above 1, FSR>1, while oxidative addition complexes are characterized by FSR = 1 or slightly less than 1.[7]

Covalent radii can also be used as an alternative to predict the length of a single bond as the sum of the atomic radii of the participating atoms:

$$R_{AB} = r_A + r_B$$

Various sets of covalent radii can be used for this purpose.[1,2,8] Álvarez and coworkers proposed in 2008 a revisited set of covalent radii for most of the elements with atomic numbers up to 96 deduced from crystallographic data deposited at the Cambridge Structural Data Base (CSD).[1] More recently, Pyykkö has provided an alternative collection of radii for covalent bonds in molecules.[2]

To appraise intermetal bonding in the LiR complexes investigated in this work, as well as in some reported complexes that might be tentatively thought of as containing a coordinated Li–C bond, we have determined their formal shortness ratio, FSR, using both the covalent radii by Alvarez and coworkers[1] and Pauling's single-bond metallic radii.[3] From now on, they will be abbreviated as FSR(r_c) and FSR(r_m), respectively. The latter are only aimed to facilitate comparison with data already in the literature.[5-7] Figures S7-S11 summarize these results.

Table S2. Values of tabulated atomic radii (r_m and r_c) for selected metals and of single-bond lengths (**s-b.l.**) determined for corresponding Li–M bonds (all values are in Å).

	**Li**	**V**	**Fe**	**Ni**	**Zr**	**Mo**	**Hf**
r_c	1.28	1.53	1.32[a]	1.24	1.75	1.54	1.75
r_m	1.225	1.224	1.170	1.154	1.454	1.296	1.442
Li–M, **s-b.l.** (r_c)	-	2.81	2.60	2.52	3.03	2.82	3.03
Li–M, **s-b.l.** (r_m)	-	2.44	2.39	2.37	2.67	2.52	2.66

r_m: Pauling single-bond metallic radius (ref. 3)

r_c: Covalent radii given by Álvarez and coworkers (ref. 1)

s-b.l. (Li–M) = r_c(Li) + r_c(M)

s-b.l. (Li–M) = r_m(Li) + r_m(M)

a, low spin value

(a)

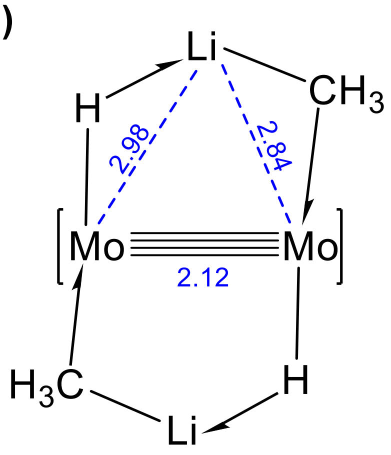

5·LiCH$_3$

Mo≡Mo

FSR (r_c) = 0.69; FSR (r_m) = 0.81

Mo–Li

using Li···Mo = 2.84

FSR (r_c) = 1.01; FSR (r_m) = 1.12

(b)

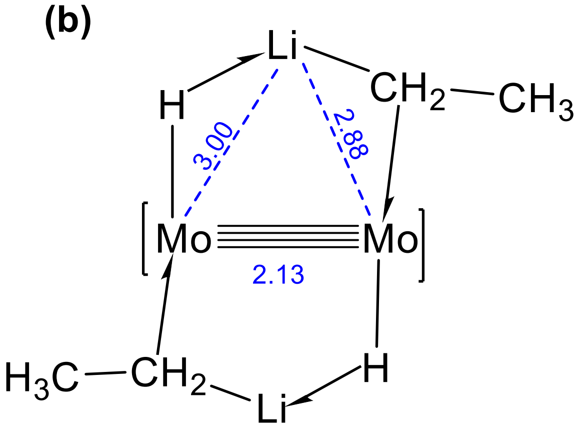

5·LiCH$_2$CH$_3$

Mo≡Mo

FSR (r_c) =0.69; FSR (r_m) = 0.82

Mo–Li

using Li···Mo = 2.88

FSR (r_c) = 1.02; FSR (r_m) = 1.14

Figure S7. Intermetal interactions in the complexes **5·LiCH$_3$** (**a**) and **5·LiCH$_2$CH$_3$** (**b**) reported in this work (all distances are in Å).

(a)

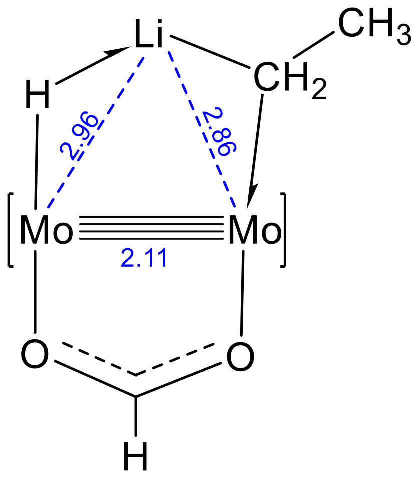

4·$LiCH_2CH_3$

Mo≡Mo

FSR (r_c) = 0.69; FSR (r_m) = 0.82

Mo–Li

using Li···Mo = 2.86

FSR (r_c) = 1.01; FSR (r_m) = 1.13

(b)

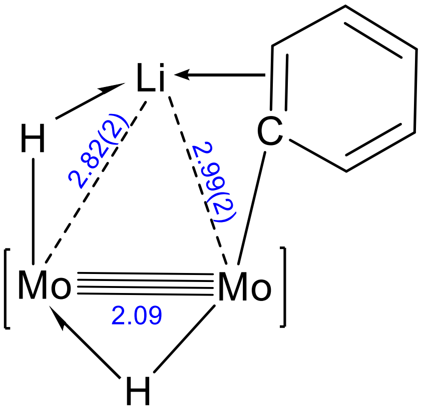

4·LiC_6H_5

Mo≡Mo

FSR (r_c) = 0.68; FSR (r_m) = 0.81

Mo–Li

using Li···Mo = 2.99

FSR (r_c) = 1.06; FSR (r_m) = 1.19

Figure S8. Intermetal interactions in the complexes **4·$LiCH_2CH_3$** (**a**) and **4·LiC_6H_5** (**b**) reported in this work (all distances are in Å).

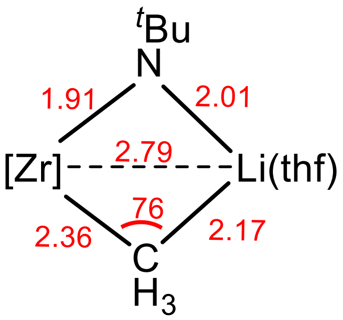

[Zr] = (η^5-C_5Me_5)$_2$Zr

FSR (r_c) = 0.92

FSR (r_m) = 1.04

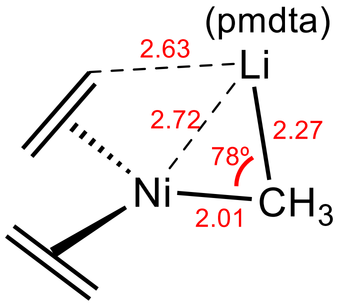

FSR (r_c) = 1.08

FSR (r_m) = 1.14

Figure S9. Formal shortness ratios (FSR) estimated for complexes {Ni(C_2H_4)$_2$[(pmdta)LiCH_3]} (ref. 9) and {(η^5-C_5Me_5)$_2$Zr(N^{t}Bu)[μ-(thf)LiCH_3]} (ref. 10) (all distances are in Å).

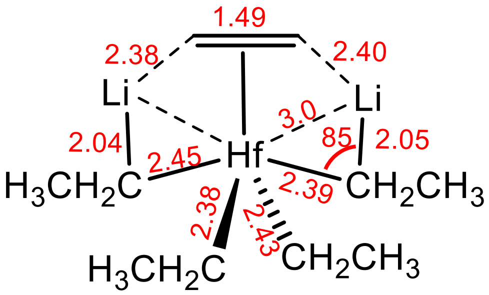

FSR (r_c) = 0.99

FSR (r_m) = 1.12

Figure S10. Formal shortness ratios (FSR) estimated for the Hf(II) complex [Li(tmeda)]$_2$[Hf(CH_2CH_3)$_4$(C_2H_4)] (ref. 11) (all distances are in Å).

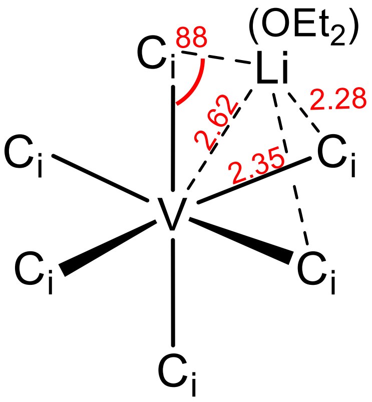

FSR (r_c) = 0.93

FSR (r_m) = 1.07

For simplicity, C_i means $C_{ipso}(C_6H_5)$ and only one of the Li atoms capping alternate C_3 faces has been represented.

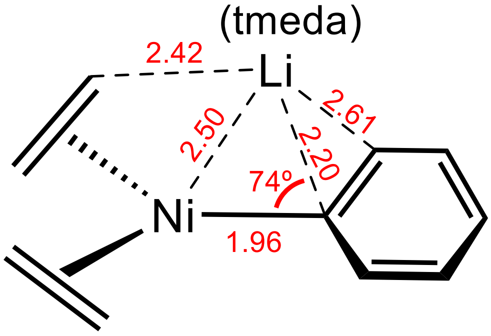

FSR (r_c) = 0.99

FSR (r_m) = 1.05

Figure S11. Formal shortness ratios (FSR) estimated for complexes $\{V(C_6H_5)_6[Li(OEt_2)]_4\}$ (top, ref. 12) and $[Li(tmeda)][Ni(C_6H_5)(C_2H_4)_2]$ (bottom, ref. 13) (all distances are in Å).

5. X-ray structural characterization of new compounds:

Single crystals of suitable size, coated with dry perfluoropolyether or FLOMBLIN oil were mounted on a glass fiber and fixed in a cold nitrogen stream [T = 193 K] to the goniometer head. Data collection was performed on a Bruker SMART APEX II CCD area detector on a D8 goniometer, using graphite-monochromated and 0.5 mm-Monocap-collimated Mo-Kα radiation (λ = 0.71073 Å) for complexes **5·LiCH$_3$**, **5·LiCH$_2$CH$_3$**, **4·LiCH$_2$CH$_3$·O$_2$CH** and a Bruker D8 Quest APEX-III CCD area detector PhotonIII using monochromatic radiation λ (Mo Kα1) = 0.71073 Å by a IµS 3.0 microfocus X-ray source for complex **4·LiC$_6$H$_5$**. Data collections were processed with APEX-W2D-NT (Bruker, 2004), cell refinement and data reduction with SAINT-Plus (Bruker, 2004) and the absorption was corrected by multiscan method applied by SADABS.[14] The space-group assignment was based upon systematic absences, E statistics, and successful refinement of the structure. The structure was solved by direct methods and expanded through successive difference Fourier maps, F^2 (SHELXTL).[15] In the last cycles of refinement, ordered non-hydrogen atoms were refined anisotropically. Hydrogen atoms connected to carbon atoms were included in idealised positions, and a riding model was used for their refinement. Mo–H hydrides of complexes **5·LiCH$_3$**, **5·LiCH$_2$CH$_3$** and **4·LiCH$_2$CH$_3$·O$_2$CH** were located in the Fourier Map and refined freely. C–H hydrogen atoms from the CH_3 and CH_2 groups of complexes **5·LiCH$_3$** and **5·LiCH$_2$CH$_3$**, respectively, were also located in the electron density map and freely refined. For complex **4·LiCH$_2$CH$_3$·O$_2$CH** the methylene hydrogen atoms were located and fixed in the electron density map. For **4·LiC$_6$H$_5$** complex, the bridging Mo(µ-H)Mo hydrogen atom was found and refined freely, whereas the Mo(µ-H)Li hydrogen atom could not be located. Complex **4·LiC$_6$H$_5$** was solved as a twinned structure with 50:50 components. TWINABS-2012/1 (Bruker,2012) was used for absorption correction.

	**5·LiCH$_3$**	**5·LiCH$_2$CH$_3$**
Formula	$C_{60}H_{90.187}Li_2Mo_2N_4O_2$	$C_{62}H_{98}Li_2Mo_2N_4O_2$
fw	1105.30	1137.20
Crystal size, mm	0.24x0.16x0.14	0.25x0.11x0.10
Crystal system	monoclinic	orthorhombic
Space group	P 1 21/n 1	P b c a
a, Å	11.9659(11)	21.1195(6)
b, Å	13.0752(12)	12.8082(4)
c, Å	19.0870(17)	22.6436(8)
α, deg	90	90
β, deg	92.800(4)	90
γ, deg	90	90
V, Å^3	2982.7(5)	6125.2(3)
T, K	193(2)	193(2)
Z	2	4
ρ_{calc}, g·cm^{-3}	1.231	1.233
μ, mm^{-1} (Mo1Kα)	0.462	0.452
F (000)	1168	2416
Absoption correction	multi-scan 0.6371- 0.7461	multi-scan 0.6430- 0.7461
θ range, deg	2.509- 30.626	1.929-30.496
No. of rflns measd	9132	9336
R_{int}	0.0393	0.0362
No. of rlfns unique	9132	9336
No. of params/restraints	382/199	343/35
R1 (I > 2σ(I))	0.0336	0.0455
R1 (all data)	0.0576	0.0791
wR2 (I > 2σ(I))	0.0709	0.1098
wR2 (all data)	0.0788	0.1266
Diff. Fourier peaks min/max, eÅ^{-3}	-0.440/0.563	-2.060/1.732
CCDC number	2090048	2090049

	**4·LiCH$_2$CH$_3$·O$_2$CH**	**4·LiC$_6$H$_5$**
Formula	$C_{57}H_{85}LiMo_2N_4O_3$	$C_{64}H_{96}LiMo_2N_4O_2$
fw	1073.10	1152.26
Crystal size, mm	0.24x0.24x0.24	0.24x0.2x0.03
Crystal system	monoclinic	triclinic
Space group	P 1 21/n 1	P -1
a, Å	13.4693(10)	13.563(2)
b, Å	18.1751(13)	15.404(2)
c, Å	23.2721(16)	15.607(2)
α, deg	90	84.899(5)
β, deg	91.415(3)	83.349(5)
γ, deg	90	78.630(5)
V, Å^3	5695.4(7)	3167.8(8)
T, K	193(2)	193(2)
Z	4	2
ρ_{calc}, g·cm^{-3}	1.251	1.208
μ, mm^{-1} (Mo1Kα)	0.484	0.438
F (000)	2264	1222
Absoption correction	multi-scan 0.5801-0.7461	multiscan 0.501818-0.745687
θ range, deg	1.422-30.501	1.950-25.499
No. of rflns measd	17287	11572
R_{int}	0.0626	0.0797
No. of rlfns unique	17287	11572
No. of params/restraints	627/49	681/27
R1 (I > 2σ(I))	0.0503	0.0786
R1 (all data)	0.0826	0.1078
wR2 (I > 2σ(I))	0.1265	0.1772
wR2 (all data)	0.1540	0.1945
Diff. Fourier peaks min/max, eÅ^{-3}	-1.228/1.885	-1.352/0.985
CCDC number	2090050	2090051

6. Synthesis and characterization of new complexes

General Considerations:

All manipulations were carried out using standard Schlenk (under argon) and glovebox techniques (under hight purity nitrogen). All solvents were dried and degassed prior to use. Toluene (C_7H_8) and *n*-pentane (C_5H_{12}) were distilled under nitrogen over sodium. Tetrahydrofuran (thf) and diethyl ether were distilled under nitrogen over sodium/benzophenone. Benzene-d_6 was dried over 4 Å molecular sieves. Solution NMR spectra were recorded on Bruker DRX-400 and DRX-500 spectrometers and in a Bruker Avance III 500 MHz equipped with a CryoProbe (Citius). Spectra were referenced to external $SiMe_4$ (δ: 0 ppm) using the residual proton solvent peaks as internal standards (^1H NMR experiments), or the characteristic resonances of the solvent nuclei (^{13}C NMR experiments). Spectral assignments were made by routine one- and two-dimensional NMR experiments, where appropriate. For elemental analyses a LECO TruSpec CHN elementary analyser was utilised. Infrared spectra were recorded on a Bruker Vector 22 spectrometer and sampling preparation was made in Nujol. Complex $[Mo_2(H)_2(\mu\text{-}Ad^{Dipp2})_2(thf)_2]$ and $[Mo_2(H)(\mu\text{-}O_2CH)(\mu\text{-}Ad^{Dipp2})_2(thf)]$ were prepared as reported in the literature.[16,17]

{Mo_2[HLi(thf)CH_3]$_2$(μ-Ad^{Dipp2})$_2$} (5·$LiCH_3$)

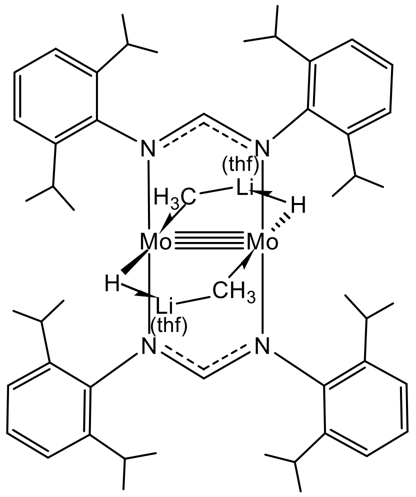

Complex [$Mo_2(H)_2$(μ-Ad^{Dipp2})$_2$(thf)$_2$] (50 mg, 0.05 mmol) and [CH_3Li]$_4$ (2.4 mg, 0.028 mmol) were dissolved in thf (4 mL) at room temperature (molar ratio, **1·thf**:CH_3Li, 1:2.2). The solution was stirred for one hour and the solvent was removed under vacuum. Toluene (2 mL) was added over the dark solid and the yellow solution formed was filtered. The solution was concentrated to about half the initial volume and kept still for hours. Yellow crystals appeared at room temperature after a few hours (21 mg, 38 % yield). LiAd^{Dipp2} was identified as a by-product forming during the reaction time. The product is highly insoluble in common solvents as benzene, toluene, fluorobenzene or thf and decomposes in more polar ones as dichloromethane. The product is unstable in solution and decomposes partially over time ($t_{1/2} \approx$ 24 h, 25 °C) forming LiAd^{Dipp2}.

[CH_3Li]$_4$ and [$^{13}CH_3Li$]$_4$ were prepared from methyl iodide or ^{13}C-methyl iodide by treatment with equimolar quantities of *n*-butyllithium in hexane solutions.[18]

^{1}H NMR (500 MHz, C_6D_6, 25 °C) δ (ppm): -0.80 (br s, 6 H, Li–CH_3–Mo, becomes a sharper singlet in ^{1}H{^{7}Li} NMR spectrum), 1.02, 1.26, 1.33, 1.41 (d, 48 H, 12 H each, $^3J_{HH}$ = 6.9 Hz, CH*Me*$_2$), 1.25 (br, OCH_2CH_2), 3.70 (br m, 2 H, Mo–H–Li, becomes a br singlet in ^{1}H{^{7}Li} NMR spectrum), 3.70 (br, OCH_2CH_2), 3.29, 4.53 (sept, 8 H, 4 H each, $^3J_{HH}$ = 6.9 Hz, C*H*Me$_2$), 7.11-7.21 (m, 12 H, *p, m*-Dipp), 8.24 (s, 2 H, NC(H)N).

^{7}Li{^{1}H} NMR (161 MHz, C_6D_6, 25 °C) δ (ppm): 4.8 (br); ^{7}Li NMR: 4.8 (d, $^1J_{LiH}$ = 20 Hz).

^{13}C{^{1}H} NMR (100 MHz, C_6D_6, 25 °C) δ (ppm): 24.8, 25.0, 25.4, 25.7, 28.2, 28.7, 29.0 (CH*Me*$_2$, OCH_2CH_2, *C*HMe$_2$), 68.6 (OCH_2CH_2, detected by ^{1}H-^{13}C HSQC), 123.4, 124.2,

124.7 (*m*-Dipp, *p*-Dipp), 159.9 (NC(H)N, detected by ^{1}H-^{13}C HSQC). *o*-Dipp, *ipso*-Dipp were not detected.

Elemental analysis (%): Calc. for $C_{60}H_{94}Li_2Mo_2N_4O_2$: C, 65.0; H, 8.5; N, 5.1; Expt.: C, 64.9; H, 8.4; N, 5.4.

For enriched in ^{13}C sample:

^{1}H NMR (500 MHz, C_6D_6, 25 °C) δ (ppm): -0.80 (d, 6 H, Li–CH_3–Mo, $^{1}J_{CH}$ = 109 Hz), 3.70 (br, 2 H, Mo–H–Li, becomes a br s in $^{1}H\{^{7}Li\}$ NMR spectrum).

DEPT-135 NMR (125 MHz, cryoprobe, C_6D_6, 25 °C) δ (ppm): 0.6 (Li–CH_3–Mo, $^{1}J_{CH}$= 109 Hz, $^{1}J_{C\text{-}7Li}$= 16 Hz, $^{1}J_{C\text{-}6Li}$= 6 Hz).

$^{7}Li\{^{1}H\}$ NMR (161 MHz, C_6D_6, 25 °C) δ (ppm): 4.8 (d, $^{1}J_{13C\text{-}7Li}$= 16 Hz); ^{7}Li NMR: 4.8 (m).

{Mo_2(μ-H)[HLi(thf)CH_3](μ-Ad^{Dipp2})$_2$} (4·$LiCH_3$)

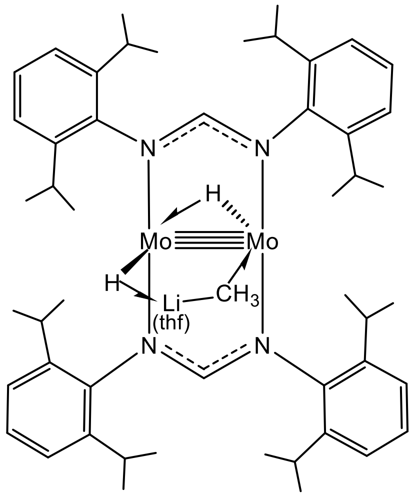

Complex [$Mo_2(H)_2$(μ-Ad^{Dipp2})$_2$(thf)$_2$] (20 mg, 0.02 mmol) and [$LiCH_3$]$_4$ (0.5 mg, 0.006 mmol) were dissolved in C_6D_6 in a Young NMR tube inside the glovebox (molar ratio, **1·thf**:$LiCH_3$, 1:1.2). The solution turned dark red immediately and by ^{1}H NMR experiments the formation of the intermediate species {Mo_2(μ-H)[HLi(thf)CH_3](μ-Ad^{Dipp2})$_2$} was detected. The complex was not isolated, but characterized by NMR in solution. The same experiment was performed with labelled [$^{13}CH_3Li$]$_4$ to obtain C–Li coupling constants.

^{1}H NMR (500 MHz, C_6D_6, 25 °C) δ (ppm): -0.79 (br s, 3 H, C*H$_3$*Li), 0.86, 0.94, 1.13, 1.26, 1.30, 1.35, 1.43, 1.47 (d, 6 H each, 48 H, $^3J_{HH}$ = 6.6 Hz, CH*Me$_2$*), 1.25, 3.54 (br, OCH_2CH_2, OCH_2CH_2), 3.63, 4.15, 4.27, 4.38 (sept, 2 H each, 8 H, $^3J_{HH}$ = 6.6 Hz, C*H*Me$_2$), 4.55 (d, 1 H, Mo–H, $^2J_{HH}$ = 8 Hz), 5.26 (br m, 1 H, Li–H–Mo, becomes a d in ^{1}H{^{7}Li} NMR spectrum, $^2J_{HH}$ = 8 Hz), 7.07-7.16 (m, 12 H, *m, p*-Dipp), 8.56 (s, 2 H, NC(H)N).

^{7}Li{^{1}H} NMR (161 MHz, C_6D_6, 25 °C) δ (ppm): 3.2 (br); ^{7}Li NMR: 3.2 (d, $^1J_{HLi}$ = 20 Hz).

For enriched in ^{13}C sample:

^{1}H NMR (500 MHz, C_6D_6, 25 °C) δ (ppm): -0.79 (br d, 3 H, C*H$_3$*Li, $^1J_{CH}$ = 110 Hz), 4.55 (dd, 1 H, Mo–H, $^2J_{HH}$ = 8 Hz, $^2J_{CH}$ = 10 Hz), 5.26 (br m, 1 H, Li–H–Mo, becomes a d in ^{1}H{^{7}Li} NMR spectrum, $^2J_{HH}$ = 8 Hz).

^{7}Li{^{1}H} NMR (161 MHz, C_6D_6, 25 °C) δ (ppm): 3.2 (d, $^1J_{13C\text{-}7Li}$ = 16 Hz); ^{7}Li NMR: 3.2 (br m).

^{13}C{^{1}H} NMR (100 MHz, C_6D_6, 25 °C) δ (ppm): 0.9 (CH_3Li), 14.2, 14.6, 24.2, 24.4, 25.2, 25.7, 25.9, 26.1 (CH*Me$_2$*), 25.8 (OCH_2*C*H_2), 28.0, 28.2, 28.5, 28.8 (*C*HMe$_2$), 68.9

(OCH$_2$CH$_2$), 123.0, 123.5, 123.6, 123.8, 125.7, 126.0 (*m*-Dipp, *p*-Dipp), 143.3, 144.0, 144.7, 145.0, 145.3, 146.7 (*ipso*-Dipp, *o*-Dipp), 160.3 (NC(H)N).

DEPT-135 NMR (125 MHz, cryoprobe, C_6D_6, 25 °C) δ (ppm): 0.9 (CH_3Li, $^1J_{C\text{-}7Li}$ = 16 Hz, $^1J_{C\text{-}6Li}$ = 6 Hz).

{$Mo_2[HLi(thf)CH_2CH_3]_2(\mu\text{-}Ad^{Dipp2})_2$} (5·$LiCH_2CH_3$)

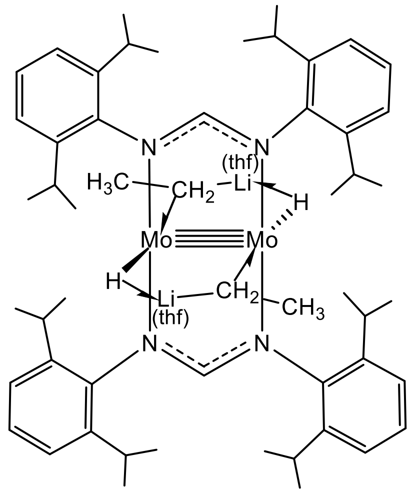

0.3 mL of a $LiCH_2CH_3$ solution (0.11 mmol, 0.35 M solution in benzene:cyclohexane, 90:10) was added to a solution of $[Mo_2(H)_2(\mu\text{-}Ad^{Dipp2})_2(thf)_2]$ (50 mg, 0.05 mmol) dissolved in toluene (5 mL) at room temperature. The yellow solution was stirred for 30 minutes and the solvent was removed under vacuum to about half its volume inside a glovebox and left still for hours. Yellow crystals appeared at room temperature that were isolated in 40 % yield (23 mg).

For a ^{13}C enriched sample: $[Li^{13}CH_2CH_3]_4(OEt_2)_4$ was prepared from $CH_3{}^{13}CH_2I$ by treatment at low temperature (-70 °C) in diethyl ether with equimolar amounts of *t*-butyllithium, 1.7 M solution in pentane.[19] 20 mg (0.02 mmol) of $[Mo_2(H)_2(\mu\text{-}Ad^{Dipp2})_2(thf)_2]$ and 4.4 mg (0.01 mmol) of $[Li^{13}CH_2CH_3]_4(OEt_2)_4$ were dissolved in toluene (1 mL) and stirred for 30 minutes (molar ratio, **1·thf**:$Li^{13}CH_2CH_3$, 1:2), the yellow precipitate was decanted, separated from the solution, dried and dissolved in C_6D_6 for NMR experiments.

^1H NMR (500 MHz, C_6D_6, 25 °C) δ (ppm): -0.59 (t, 6 H, $^3J_{HH}$ = 7.5 Hz, Li–CH_2–C*H*$_3$), 0.56 (q, 4 H, $^3J_{HH}$ = 7.5 Hz, Li–C*H*$_2$–CH_3), 1.13, 1.21, 1.34, 1.46 (d, 48 H, 12 H each, $^3J_{HH}$ = 6.9 Hz, CH*Me*$_2$), 1.30, 3.73 (br, OCH_2C*H*$_2$, OC*H*$_2CH_2$), 3.74 (br, 2 H, Mo–H–Li, becomes a br singlet in $^1H\{^7Li\}$ NMR spectrum), 3.44, 4.70 (sept, 8 H, 4 H each, $^3J_{HH}$ = 6.9 Hz, C*H*Me_2), 7.11-7.19 (m, 12 H, *p, m*-Dipp), 8.24 (s, 2 H, NC(H)N).

DEPT-135 NMR (125 MHz, cryoprobe, C_6D_6, 25 °C) δ (ppm): 4.0 ($^1J_{CH}$ = 124 Hz, CH_3), 6.1 ($^1J_{CH}$ = 112 Hz, CH_2), 23.6, 24.1, 24.7, 25.4 (CH*Me*$_2$), 25.5 (OCH_2CH_2), 28.4, 29.0 (*C*HMe_2), 68.7 (O*C*H_2CH_2, detected by ^1H-^{13}C HSQC) 123.0, 123.8, 124.6 (*m*-Dipp, *p*-Dipp), 159.7

(NC(H)N, detected by ^{1}H-^{13}C HSQC). *o*-Dipp, *ipso*-Dipp were not detected. CH coupling constants were measured by ^{1}H-^{13}C HSQC coupled experiments.

$^{7}Li\{^{1}H\}$ NMR (161 MHz, C_6D_6, 25 °C) δ (ppm): 4.3 (br s); ^{7}Li NMR: 4.3 (d, $^{1}J_{LiH}$ = 21 Hz).

Elemental analysis (%): Calc. for $C_{62}H_{98}Li_2Mo_2N_4O_2$: C, 65.5; H, 8.7; N, 4.9; Expt.: C, 65.1; H, 8.6; N, 4.9.

For enriched in ^{13}C sample:

^{1}H NMR (500 MHz, C_6D_6, 25 °C) δ (ppm): -0.59 (td, 6 H, $^{3}J_{HH}$ = 7.5 Hz, $^{2}J_{CH}$ = 3 Hz, Li–$^{13}CH_2$–CH_3), 0.56 (dq, 4 H, $^{1}J_{CH}$ = 112 Hz, $^{3}J_{HH}$ = 7.5 Hz, Li–^{13}CH_2–CH_3), 3.74 (br, 2 H, Mo–H–Li, becomes a br singlet in $^{1}H\{^{7}Li\}$ NMR spectrum).

$^{7}Li\{^{1}H\}$ NMR (161 MHz, C_6D_6, 25 °C) δ (ppm): 4.3 (d, $^{1}J_{13C\text{-}7Li}$ = 13 Hz); ^{7}Li NMR: 4.3 (m).

DEPT-135 NMR (125 MHz, C_6D_6, 25 °C) δ (ppm): 4.0 (s, *C*$H_3{}^{13}CH_2Li$), 6.1 (m, $^{1}J_{13C\text{-}7Li}$ = 13 Hz, $^{1}J_{13C\text{-}6Li}$ = 5 Hz, $CH_3{}^{13}$*C*H_2Li).

{Mo_2(μ-H)[HLi(thf)$^{13}CH_2CH_3$](μ-Ad^{Dipp2})$_2$} (4·$LiCH_2CH_3$)

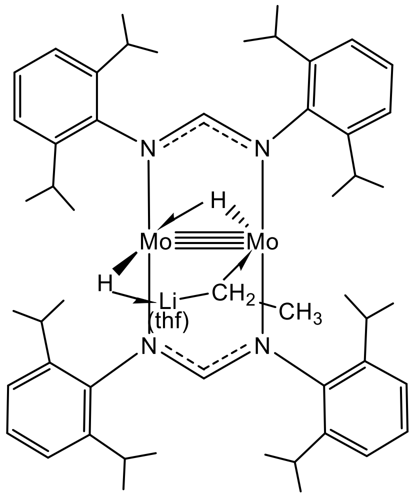

Complex [$Mo_2(H)_2$(μ-Ad^{Dipp2})$_2$(thf)$_2$] (20 mg, 0.02 mmol) and [$Li^{13}CH_2CH_3]_4(OEt_2)_4$ (2.6 mg, 0.006 mmol) were dissolved in C_6D_6 in a Young NMR tube inside the glovebox (molar ratio, **1·thf**:$LiCH_2CH_3$, 1:1.2). The solution turned dark orange and by ^{1}H NMR experiments the formation of [Mo_2(μ-H)[HLi(thf)$^{13}CH_2CH_3$](μ-Ad^{Dipp2})$_2$] was detected. The complex was not isolated, but characterised by NMR in solution.

^{1}H NMR (500 MHz, C_6D_6, 25 °C) δ (ppm): -0.71 (td, 3 H, C*H*$_3$^{13}CH$_2$Li, $^3J_{HH}$ = 7 Hz, $^2J_{CH}$ = 3 Hz), 0.46 (dq, 2 H, CH$_3$^{13}C*H*$_2$Li, $^1J_{CH}$ = 110 Hz, $^3J_{HH}$ = 7 Hz), 1.00, 1.05, 1.09, 1.34, 1.36, 1.41, 1.44, 1.51 (d, 6 H each, 48 H, $^3J_{HH}$ = 7 Hz, CH*Me*$_2$), 1.28, 3.74 (br, OCH$_2$C*H*$_2$, OC*H*$_2$CH$_2$), 3.68, 4.15, 4.28, 4.46 (sept, 2 H each, 8 H, $^3J_{HH}$ = 7 Hz, C*H*Me$_2$), 4.78 (dd, 1 H, Mo–H, $^2J_{HH}$ = 7 Hz, $^2J_{CH}$ = 11 Hz), 5.26 (br m, 1 H, Li–H–Mo, becomes a d in ^{1}H{^{7}Li} NMR spectrum, $^2J_{HH}$ = 7 Hz), 7.05-7.14 (m, 12 H, *m, p*-Dipp), 8.54 (s, 2 H, NC(H)N).

^{7}Li{^{1}H} NMR (161 MHz, C_6D_6, 25 °C) δ (ppm): 2.8 (d, $^1J_{13C\text{-}7Li}$ = 13 Hz); ^{7}Li NMR: 2.8 (br m).

^{13}C{^{1}H} NMR (100 MHz, C_6D_6, 25 °C) δ (ppm): 6.7 (s, H$_3$*C*^{13}CH$_2$Li), 10.1 (br m, H$_3$C^{13}*C*H$_2$Li), 23.8, 23.9, 25.0, 25.4, 25.6, 25.8, 26.1, 26.1 (CH*Me*$_2$), 24.4 (OCH$_2$*C*H$_2$), 27.8, 27.9, 28.3, 28.9 (C*H*Me$_2$), 69.9 (O*C*H$_2$CH$_2$), 123.1, 123.3, 123.4, 123.7, 125.5, 125.9 (*m*-Dipp, *p*-Dipp), 143.3, 144.0, 144.3, 144.7, 145.3, 147.0 (*ipso*-Dipp, *o*-Dipp), 160.0 (NC(H)N).

{Mo_2(μ-O_2CH)[HLi(thf)CH_2CH_3](μ-Ad^{Dipp2})$_2$} (4·LiCH_2CH_3·O_2CH)

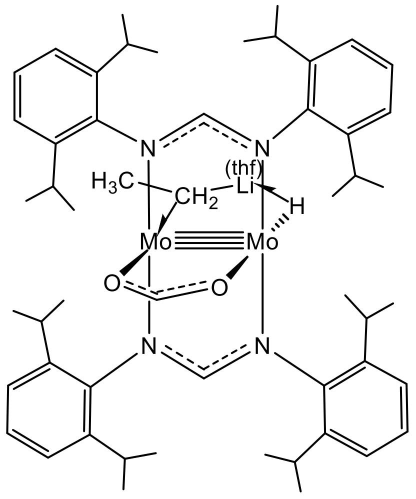

Complex [Mo_2(H)(μ-O_2CH)(μ-Ad^{Dipp2})$_2$(thf)] (50 mg, 0.05 mmol) was dissolved in toluene and 0.15 mL of a LiCH_2CH_3 solution (0.05 mmol, 0.35 M solution in benzene:cyclohexane, 90:10) was added slowly at -20 °C. The mixture was allowed to reach room temperature in a period of about 4 hours, then concentrated and kept at -30 °C. The yellow solid that precipitated was washed with pentane and isolated in a 30% yield (16 mg). Crystals suitable for X-ray diffraction studies were obtained from a saturated solution in benzene at room temperature.

^{1}H NMR (500 MHz, C_6D_6, 25 °C) δ (ppm): -0.70 (t, 3 H, $^3J_{HH}$ = 7.6 Hz, Li–CH_2–C*H*$_3$), 0.58 (q, 2 H, $^3J_{HH}$ = 7.6 Hz, Li–CH_2), 0.92, 1.17, 1.23, 1.27, 1.32, 1.36, 1.43, 1.47 (d, 48 H, 6 H each, $^3J_{HH}$ = 6.9 Hz, CH*Me*$_2$), 1.41, 3.74 (br, OCH$_2$C*H*$_2$, OC*H*$_2$CH$_2$), 3.46, 3.64, 3.76, 4.48 (sept, 8 H, 2 H each, $^3J_{HH}$ = 6.9 Hz, C*H*Me$_2$), 4.09 (m, 1 H, Mo–H–Li), 7.02-7.18 (m, 12 H, *m, p*-Dipp), 8.25 (s, 2 H, NC(H)N), 8.80 (s, 1 H, O_2CH).

^{13}C{^{1}H} NMR (125 MHz, cryoprobe, C_6D_6, 25 °C) δ (ppm): 4.1 (CH_3, $^1J_{CH}$ = 127 Hz), 8.0 (br, CH_2, $^1J_{CH}$ = 111 Hz), 24.1, 24.9, 25.1, 25.2, 25.6, 25.9, 27.2, 27.9 (CH*Me*$_2$), 25.5 (OCH$_2$*C*H$_2$), 28.2, 28.3, 28.8, 28.9 (*C*HMe$_2$), 69.4 (O*C*H$_2$CH$_2$), 123.4, 123.6, 124.2, 125.1, 125.6, 125.9 (*m*-Dipp, *p*-Dipp), 143.6, 144.1, 145.2, 145.4, 145.5, 147.1 (*ipso*-Dipp, *o*-Dipp), 162.9 (NC(H)N), 167.5 (O_2CH). C–H coupling constants were detected by coupled ^{1}H-^{13}C HSQC experiments. C–Li coupling constants were not observed due to the broadness of the signal.

^{7}Li{^{1}H} NMR (161 MHz, C_6D_6, 25 °C) δ (ppm): 3.3; ^{7}Li NMR (d, $^1J_{LiH}$ = 20 Hz).

Elemental analysis (%): Calc. for $C_{57}H_{85}LiMo_2N_4O_3$: C, 63.8; H, 8.0; N, 5.2; Expt.: C, 63.6; H, 8.3; N, 5.1.

{Mo_2(μ-H)[HLi(thf)C_6H_5](μ-Ad^{Dipp2})$_2$} (4·LiC_6H_5)

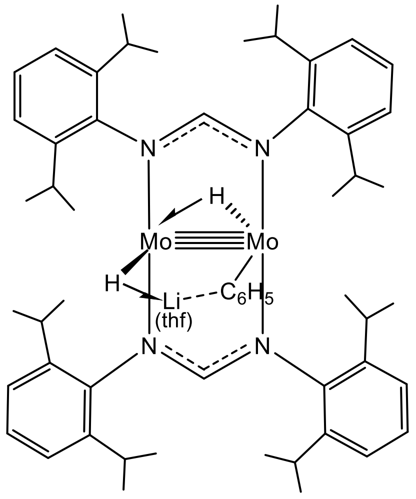

Complex [$Mo_2(H)_2$(μ-Ad^{Dipp2})$_2$(thf)$_2$] (50 mg, 0.05 mmol) was dissolved in toluene and cooled at 0 °C in an ice bath. Then, a LiC_6H_5 solution (0.03 mL, 0.06 mmol, 1.8 M in Bu_2O) was added slowly and the mixture was stirred for 5 minutes (dark red colour), when the solvent was removed under vacuum. Pentane was added to afford a brown precipitate in 60 % yield (32 mg). Crystals suitable for X-ray diffraction were obtained by placing a diethyl ether/pentane solution of the complex into the glovebox freezer at -30 °C for 2 days.

To prepare complex {Mo_2(μ-H)[HLi(thf)$^{13}C_6H_5$](μ-Ad^{Dipp2})$_2$}, ($^{13}C_6H_5Li$)$_4$(OEt_2)$_4$ was prepared from $^{13}C_6H_5Br$ by reaction with equimolar ^{t}BuLi (1.7 M solution) in hexane at -80 °C in diethyl ether.[19]

^{1}H NMR (400 MHz, C_6D_6, 25 °C) δ (ppm): 0.50, 0.58, 0.71 (d, 6 H each, 48 H, $^3J_{HH}$ = 6.8 Hz, CH*Me*$_2$), 1.21-1.33 (m, 24 H, CH*Me*$_2$ + OCH_2C*H*$_2$), 1.43 (6 H, $^3J_{HH}$ = 6.8 Hz, CH*Me*$_2$), 3.13 (br, OC*H*$_2$CH$_2$), 3.70 (d, 1 H, $^2J_{HH}$ = 8 Hz, Mo–H), 3.36, 4.02, 4.11, 4.49 (sept, 2 H each, 8 H, $^3J_{HH}$ = 6.80 Hz, C*H*Me$_2$), 6.10 (br m, Li–H–Mo, becomes a doublet, $^2J_{HH}$ = 8 Hz, in ^{1}H{^{7}Li} NMR spectrum), 6.82 (m, 1 H, *p*-PhLi), 6.91 (m, 2 H, *m*-PhLi), 7.06-7.11 (m, 12 H, *m*-Dipp, *p*-Dipp), 7.45 (m, 2 H, *o*-PhLi), 8.65 (s, 2 H, NC(H)N).

^{7}Li{^{1}H} NMR (161 MHz, C_6D_6, 25 °C) δ (ppm): 2.7 (br); ^{7}Li NMR: 2.7 (d, $^1J_{LiH}$ = 21 Hz).

^{13}C{^{1}H} NMR (100 MHz, C_6D_6, 25 °C) δ (ppm): 22.7, 23.1, 24.6, 24.9, 24.9, 25.2, 25.8, 26.9 (CH*Me*$_2$), 25.4 (OCH_2*C*H$_2$), 28.1, 28.4, 28.5, 28.6 (*C*HMe$_2$), 68.8 (O*C*H$_2$CH$_2$), 123.6, 123.8, 124.0, 124.4, 125.8, 126.0 (*m*-Dipp, *p*-Dipp), 125.6 (*p*-PhLi), 127.2 (*m*-PhLi), 141.0 (*o*-

PhLi), 143.6, 143.8, 144.3, 144.9, 145.3, 146.0 (*ipso*-Dipp, *o*-Dipp), 162.1 (NC(H)N), 179.0 (*ipso*-PhLi). *ipso*-PhLi, *p*-PhLi and *m*-PhLi were detected by HSQC and HMBC experiments.

Elemental analysis (%): Calc. for $C_{60}H_{85}LiMo_2N_4O$: C, 66.9; H, 8.0; N, 5.2; Expt.: C, 66.6; H, 8.1; N, 5.4.

For enriched ^{13}C sample:

^{1}H NMR (400 MHz, C_6D_6, 25 °C) δ (ppm): 3.70 (dd, 1 H, $^{2}J_{CH}$ = 12 Hz, $^{2}J_{HH}$ = 8 Hz, Mo–H), 6.10 (br m, Li–H–Mo, becomes a doublet, $^{2}J_{HH}$ = 8 Hz, in $^{1}H\{^{7}Li\}$ NMR spectrum).

$^{7}Li\{^{1}H\}$ NMR (161 MHz, C_6D_6, 25 °C) δ (ppm): 2.7 (d, $^{1}J_{13C\text{-}7Li}$ = 5 Hz); ^{7}Li NMR: 2.7 (br m).

$^{13}C\{^{1}H\}$ NMR (125 MHz, cryoprobe, C_6D_6, 25 °C) δ (ppm): 22.7, 23.1, 24.6, 24.9, 24.9, 25.2, 25.8, 26.9 (CH*Me*$_2$), 25.4 (OCH$_2$*C*H$_2$), 28.1, 28.4, 28.5, 28.6 (*C*HMe$_2$), 68.4 (O*C*H$_2$CH$_2$), 123.6, 123.8, 124.0, 124.4, 125.8, 126.0 (*m*-Dipp, *p*-Dipp), 125.6 (m, *p*-PhLi), 127.2 (m, *m*-PhLi), 141.0 (m, *o*-PhLi), 143.6, 143.8, 144.3, 144.9, 145.3, 146.0 (*ipso*-Dipp, *o*-Dipp), 162.1 (NC(H)N), 179.0 (t, $^{1}J_{CC}$ = 38 Hz, *ipso*-PhLi).

7. NMR spectra of new complexes

{$Mo_2[HLi(thf)CH_3]_2(\mu\text{-}Ad^{Dipp2})_2$} (5·$LiCH_3$)

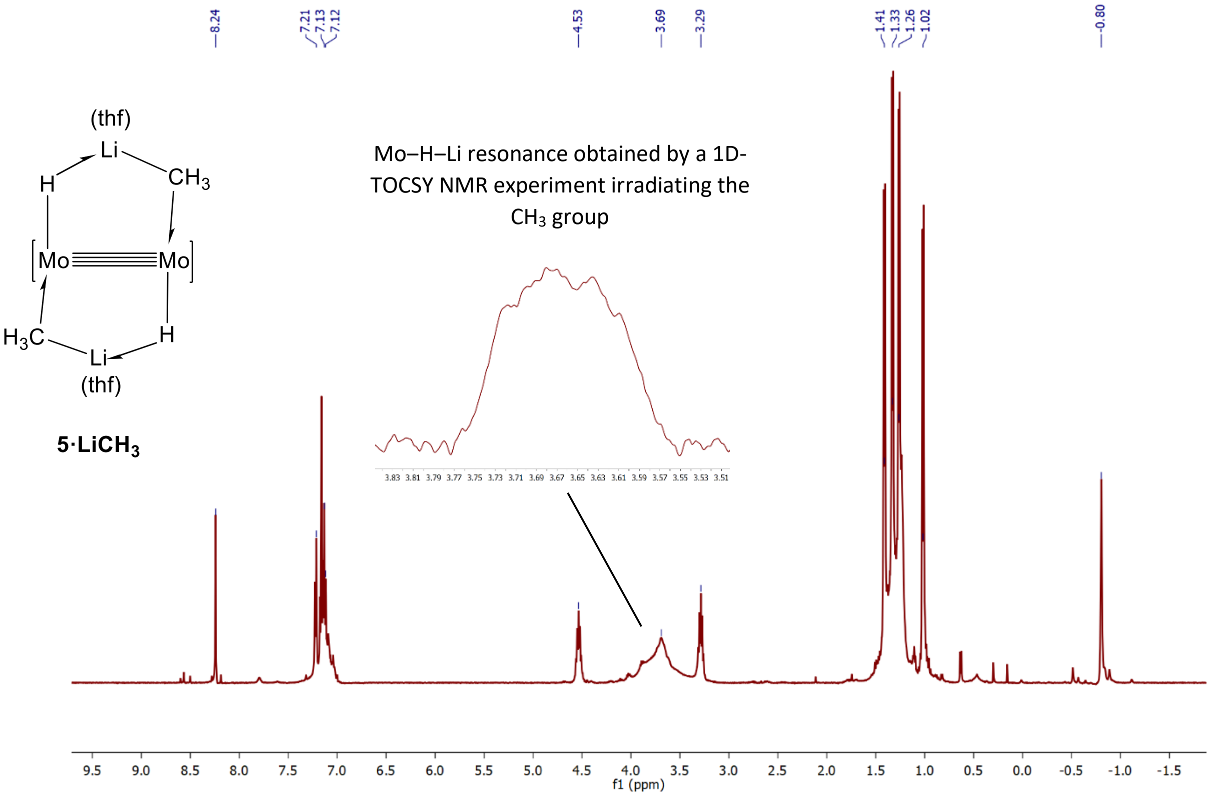

^1H NMR (500 MHz, C_6D_6, 25 °C)

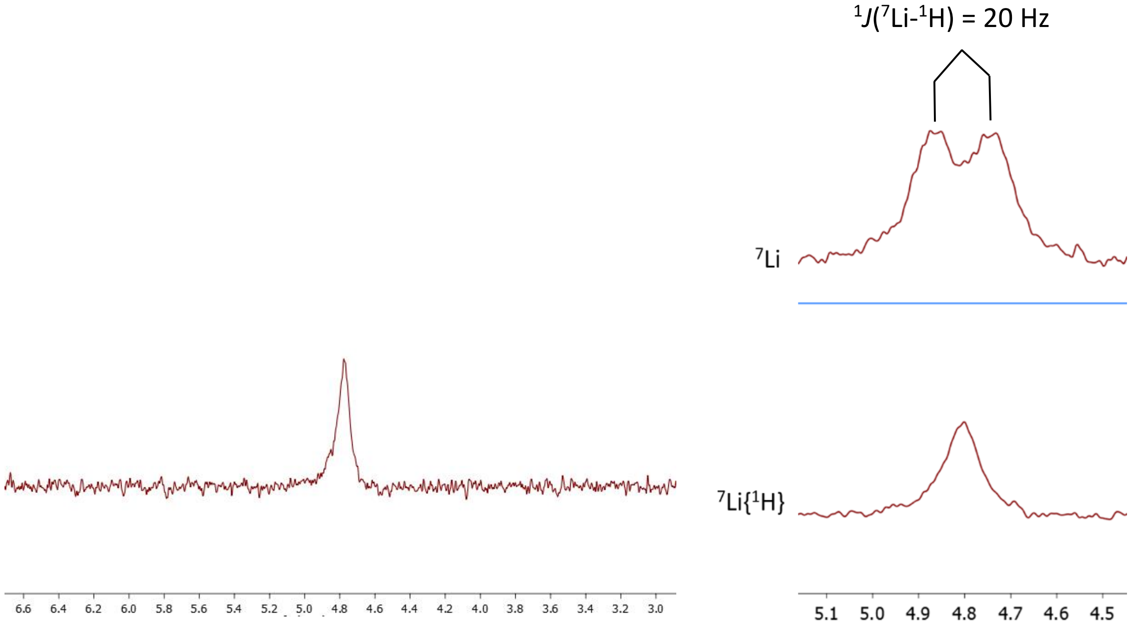

$^7Li\{^1H\}$ and ^7Li NMR (161 MHz, C_6D_6, 25 °C)

{Mo_2[HLi(thf)CH_3]$_2$(μ-Ad^{Dipp2})$_2$} (5·$LiCH_3$)

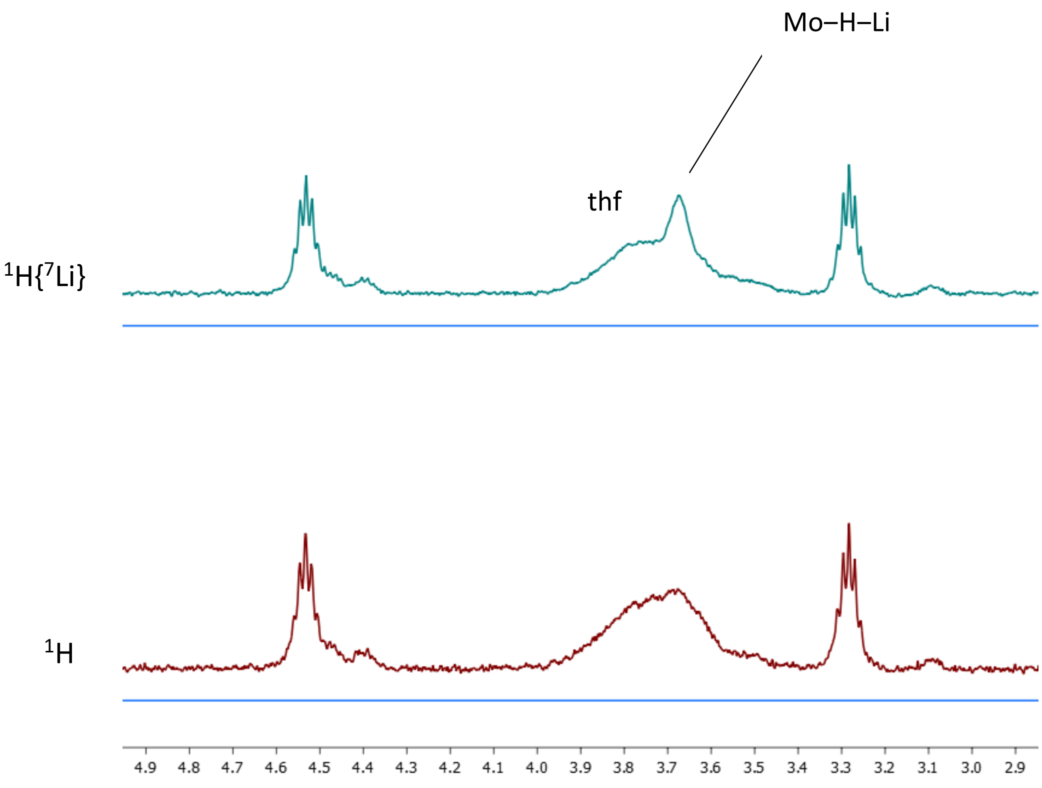

^{1}H{^{7}Li} NMR (top) and ^{1}H NMR (bottom) (500 MHz, C_6D_6, 25 °C)

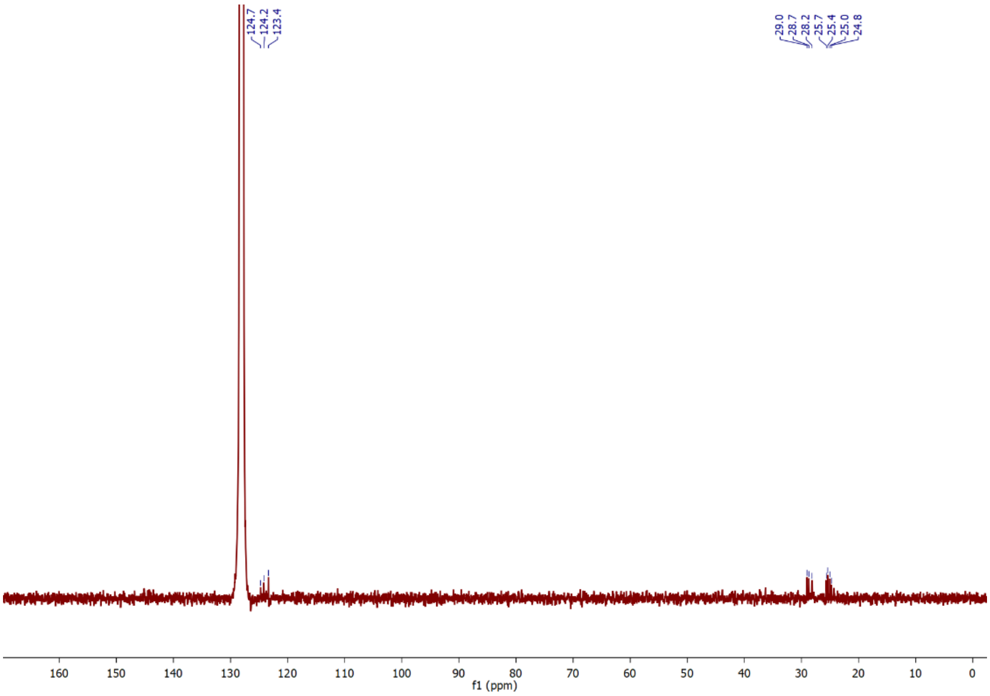

^{13}C{^{1}H} NMR (100 MHz, C_6D_6, 25 °C)

{$Mo_2[HLi(thf)^{13}CH_3]_2(\mu\text{-}Ad^{Dipp2})_2$} ($5\cdot Li^{13}CH_3$)

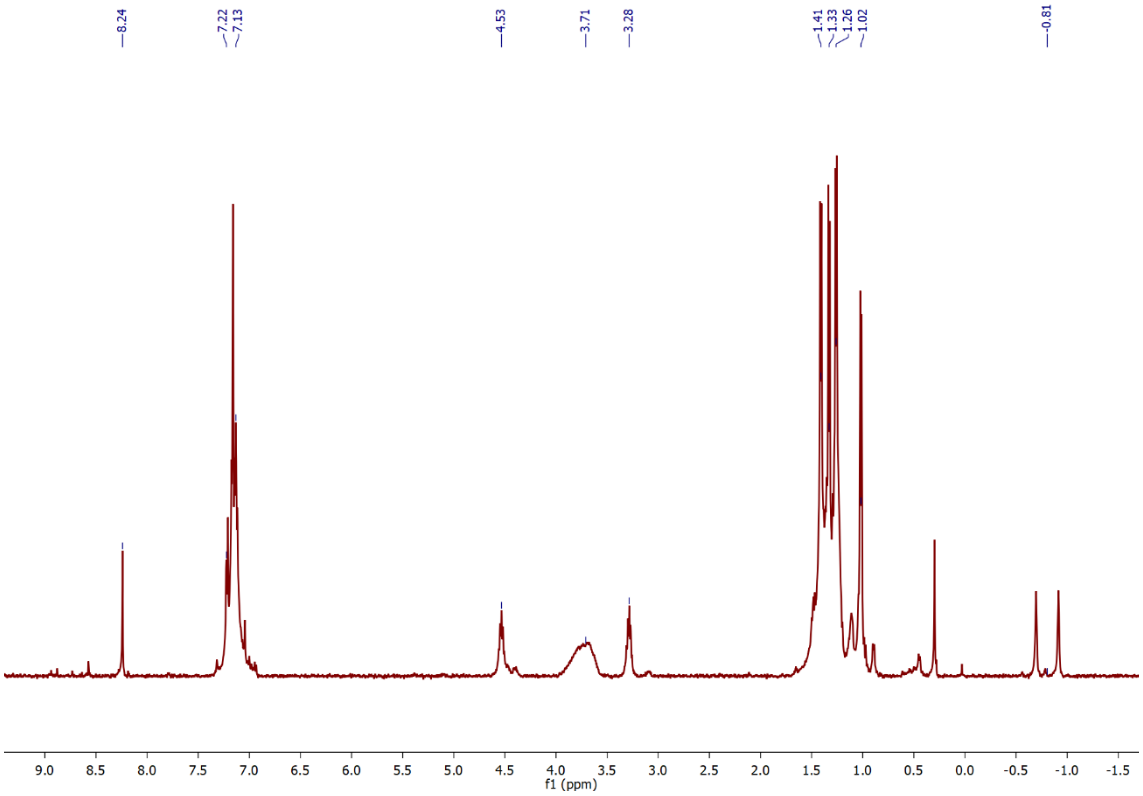

^1H NMR (500 MHz, C_6D_6, 25 °C)

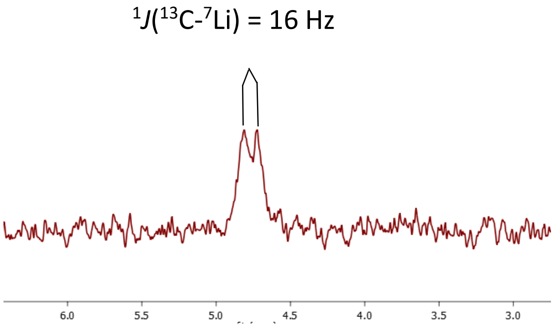

$^7Li\{^1H\}$ NMR (161 MHz, C_6D_6, 25 °C)

{$Mo_2[HLi(thf)^{13}CH_3]_2(\mu\text{-}Ad^{Dipp2})_2$} (5·$Li^{13}CH_3$)

$^1J(^{13}C\text{-}^7Li)$ = 16 Hz

$^1J(^{13}C\text{-}^6Li)$ = 6 Hz

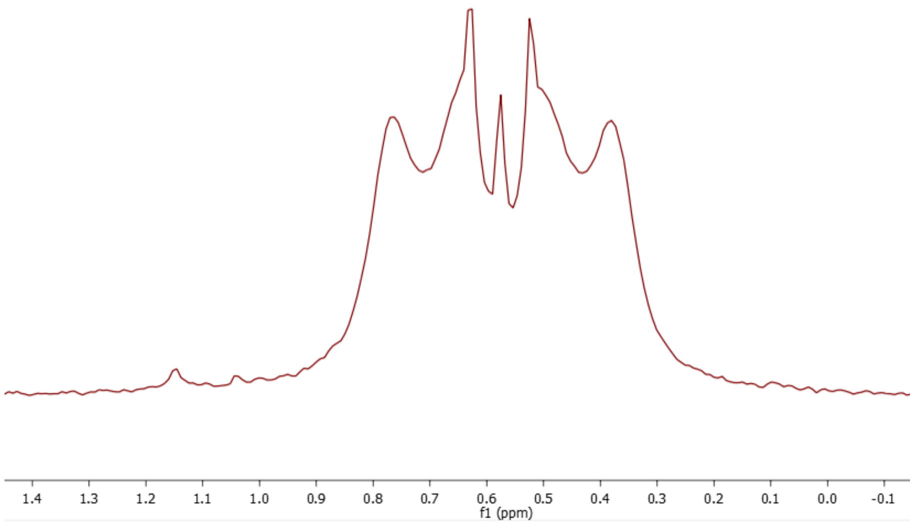

$^{13}C\{^1H\}$ DEPT-135 NMR (125 MHz, C_6D_6, 25 °C)

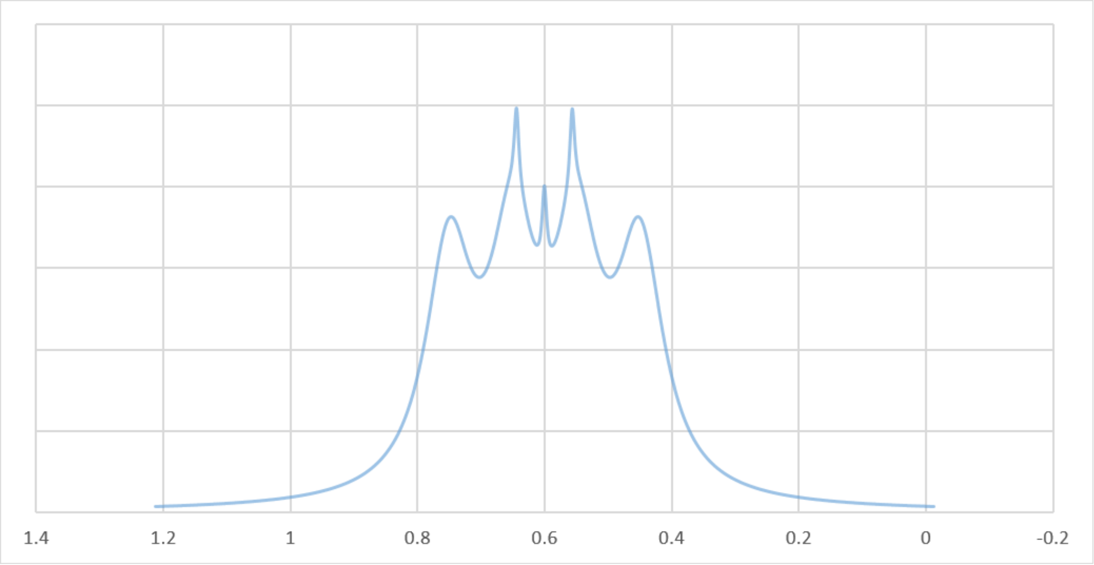

Simulated $^{13}C\{^1H\}$ NMR for the H_3C-Li signal by using the gNMR program. The simulation was done separately for ^6Li and ^7Li coupling and the sum was performed using Excel. The intensity of the signal due to the $^6Li\text{-}^{13}C$ coupling was multiplied by a factor of 0.025 to approach the experimental data. The gNMR program does not take into account the higher sensitivity of the ^6Li compared to the ^7Li nucleus that results in narrower signals for the triplet due to the coupling with ^6Li. $^1J_{CLi}$ values were obtained experimentally from the DEPT-135 NMR spectrum.

{$Mo_2[HLi(thf)CH_3](\mu\text{-}H)(\mu\text{-}Ad^{Dipp2})_2$} ($4\cdot LiCH_3$)

4·LiCH$_3$

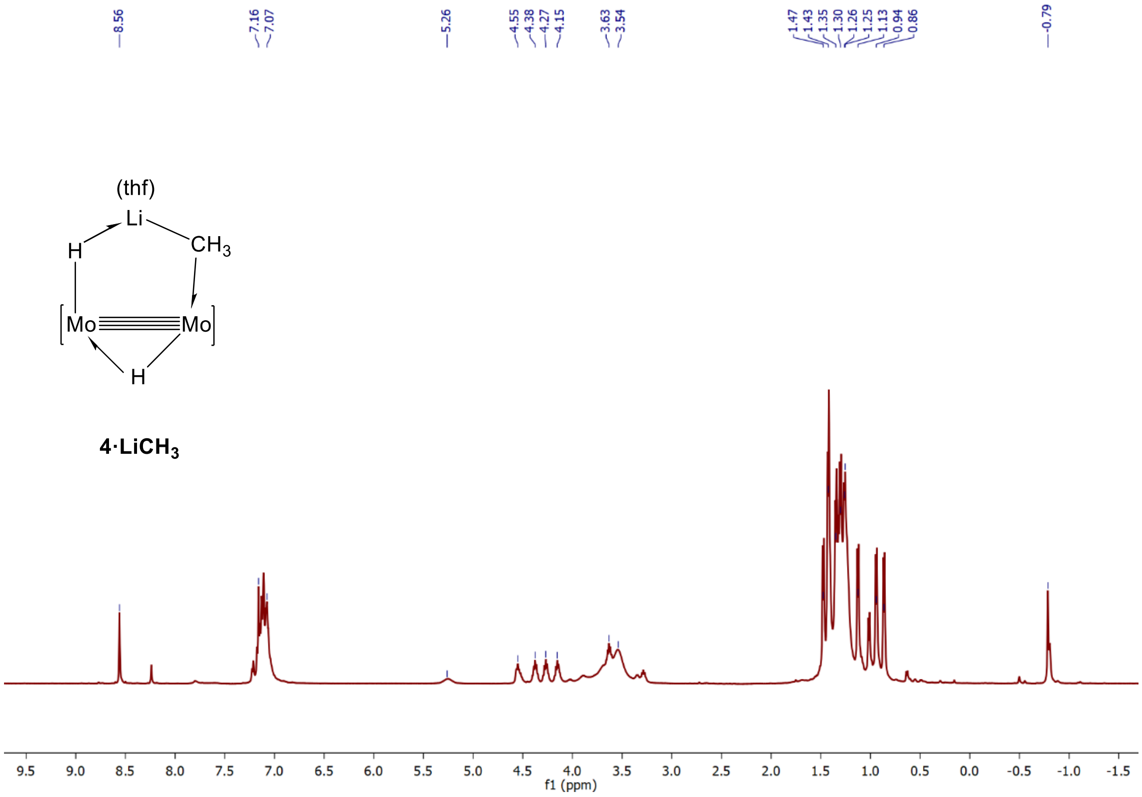

^1H NMR (500 MHz, C_6D_6, 25 °C)

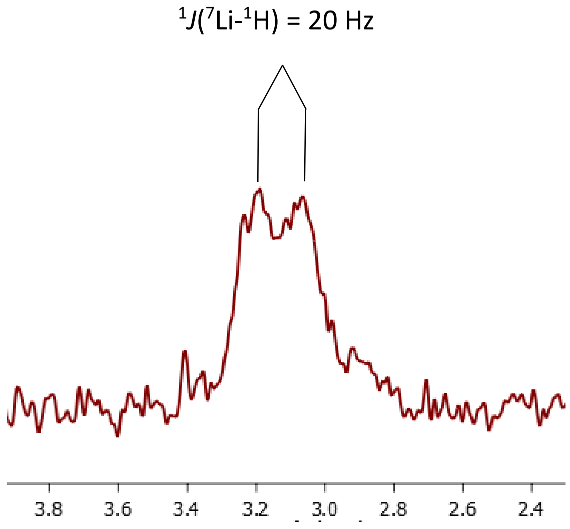

$^7Li\{^1H\}$ NMR (161 MHz, C_6D_6, 25 °C)

{Mo_2[HLi(thf)CH_3](μ-H)(μ-Ad^{Dipp2})$_2$} (4·$LiCH_3$)

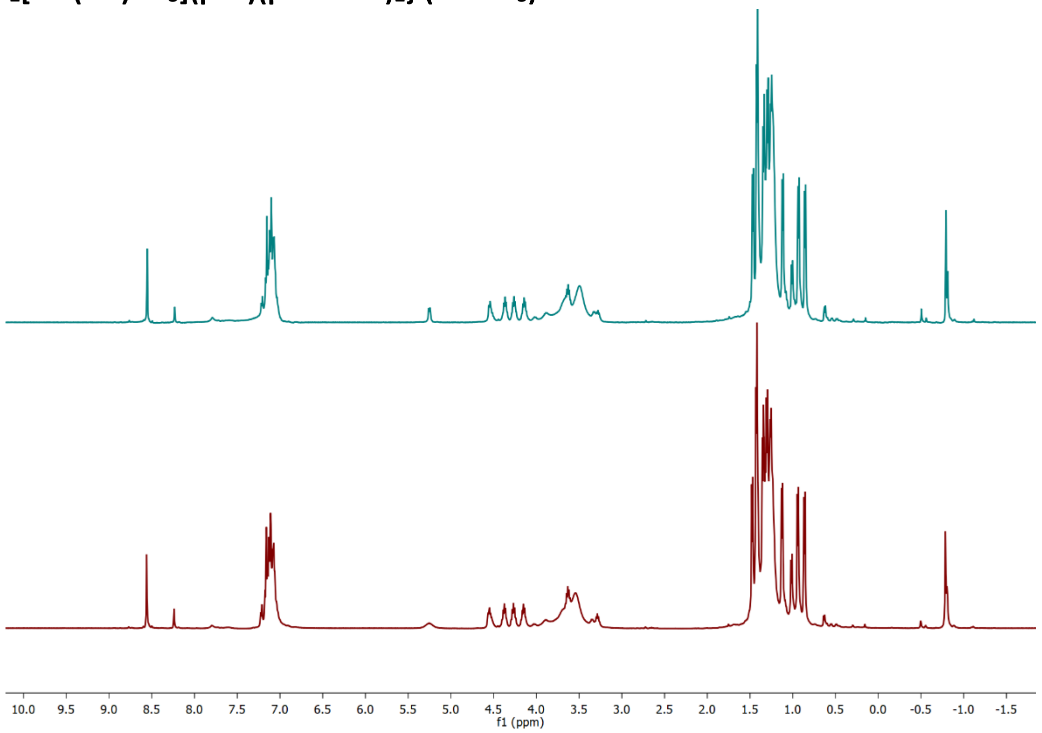

$^1H\{^7Li\}$ (top) and ^1H (bottom) NMR (500 MHz, C_6D_6, 25 °C)

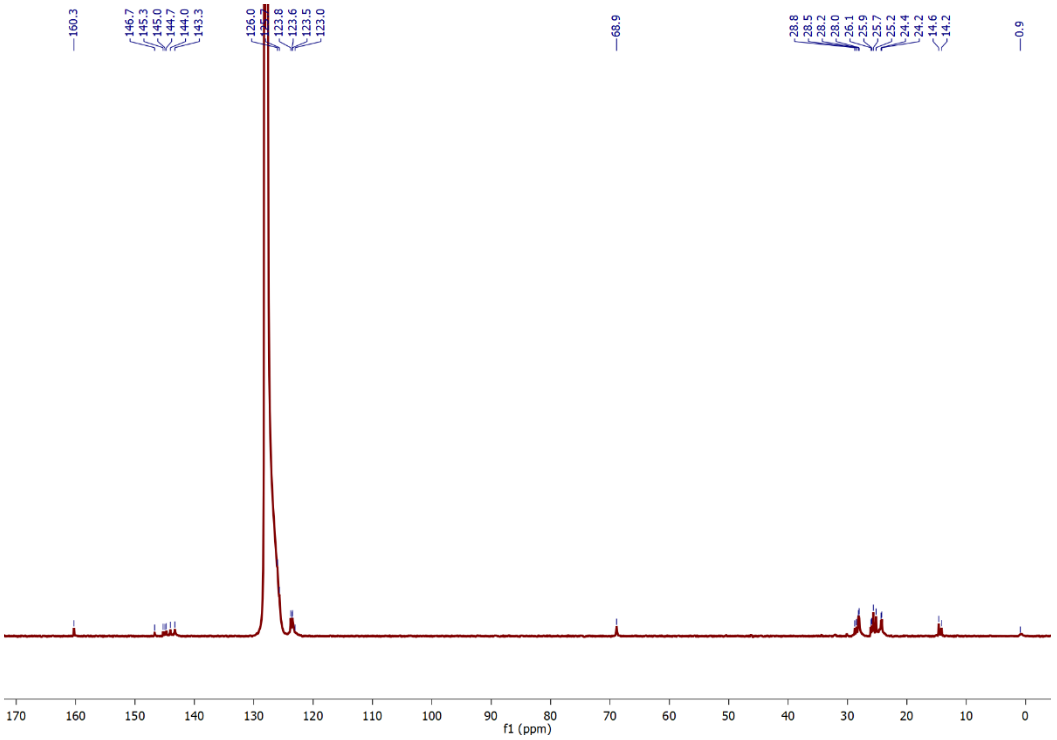

$^{13}C\{^1H\}$ NMR (100 MHz, C_6D_6, 25 °C)

$\{Mo_2[HLi(thf)^{13}CH_3](\mu\text{-}H)(\mu\text{-}Ad^{Dipp2})_2\}$ ($4{\cdot}Li^{13}CH_3$)

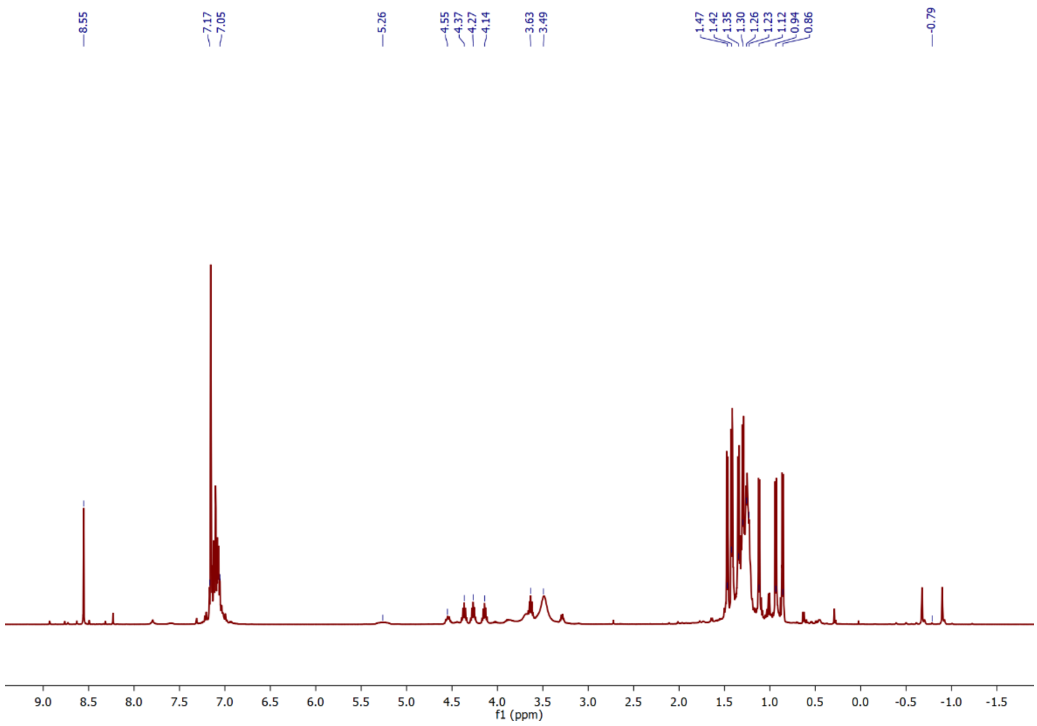

^{1}H NMR (500 MHz, C_6D_6, 25 °C)

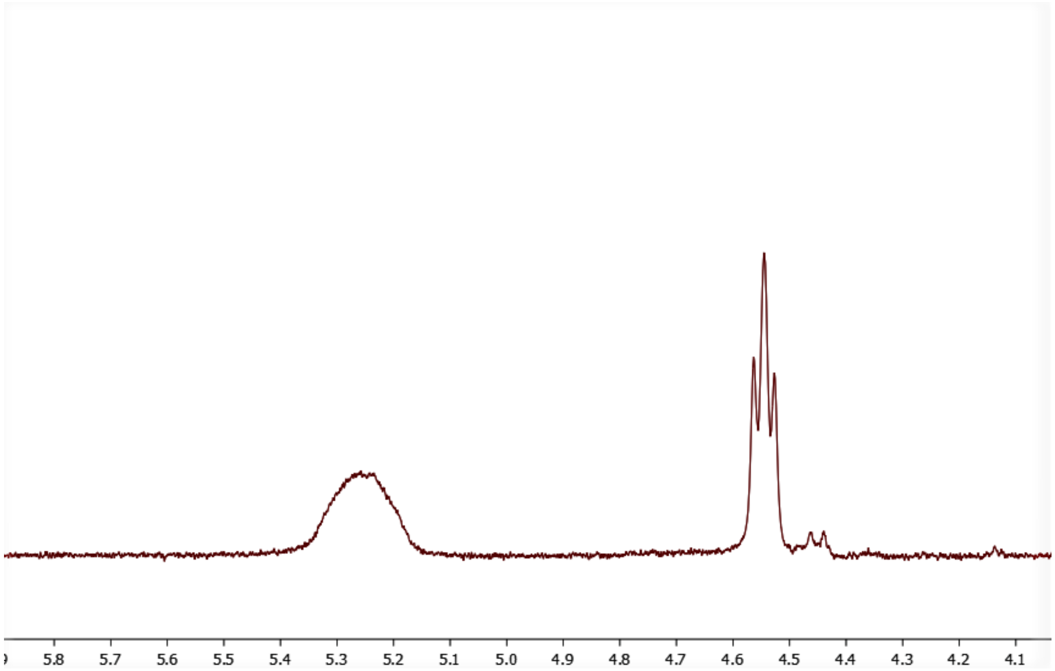

Fragment that shows the Mo–H–Li and Mo–H–Mo resonances in the ^{1}H NMR spectrum (500 MHz, C_6D_6, 25 °C)

{$Mo_2[HLi(thf)CH_2CH_3]_2(\mu\text{-}Ad^{Dipp2})_2$} (5·$LiCH_2CH_3$)

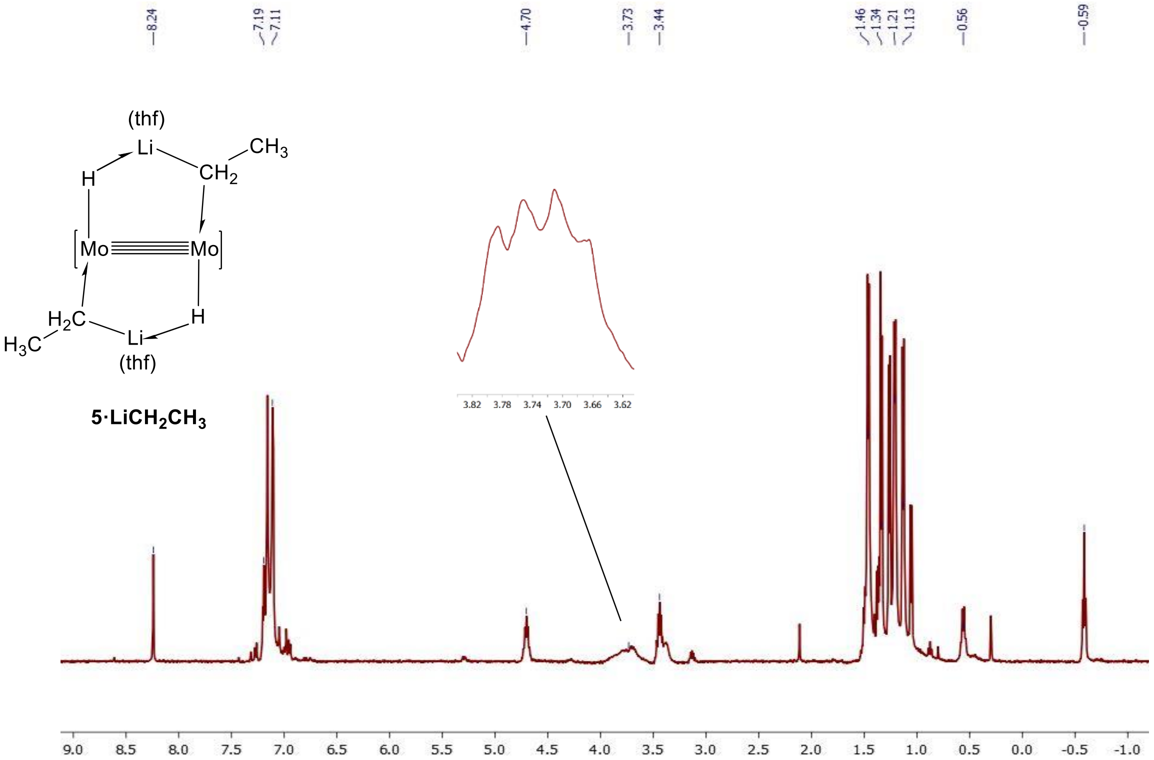

^1H NMR (500 MHz, C_6D_6, 25 °C). The caption shows the Mo–H–Li resonance obtained by a 1D-TOCSY NMR experiment, irradiating the CH_2 signal of the ethyl group.

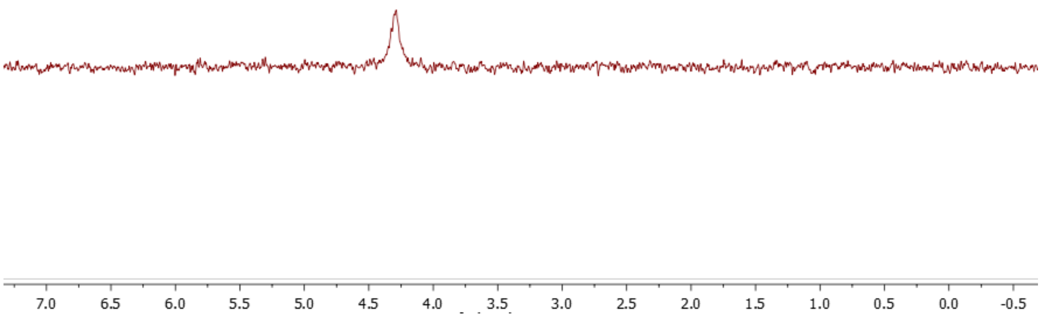

$^7Li\{^1H\}$ NMR (161 MHz, C_6D_6, 25 °C)

{$Mo_2[HLi(thf)CH_2CH_3]_2(\mu\text{-}Ad^{Dipp2})_2$} (5·$LiCH_2CH_3$)

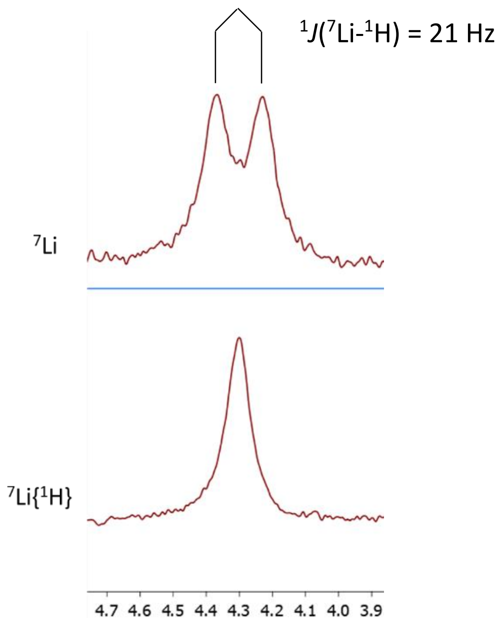

^7Li and $^7Li\{^1H\}$ NMR (161 MHz, C_6D_6, 25 °C)

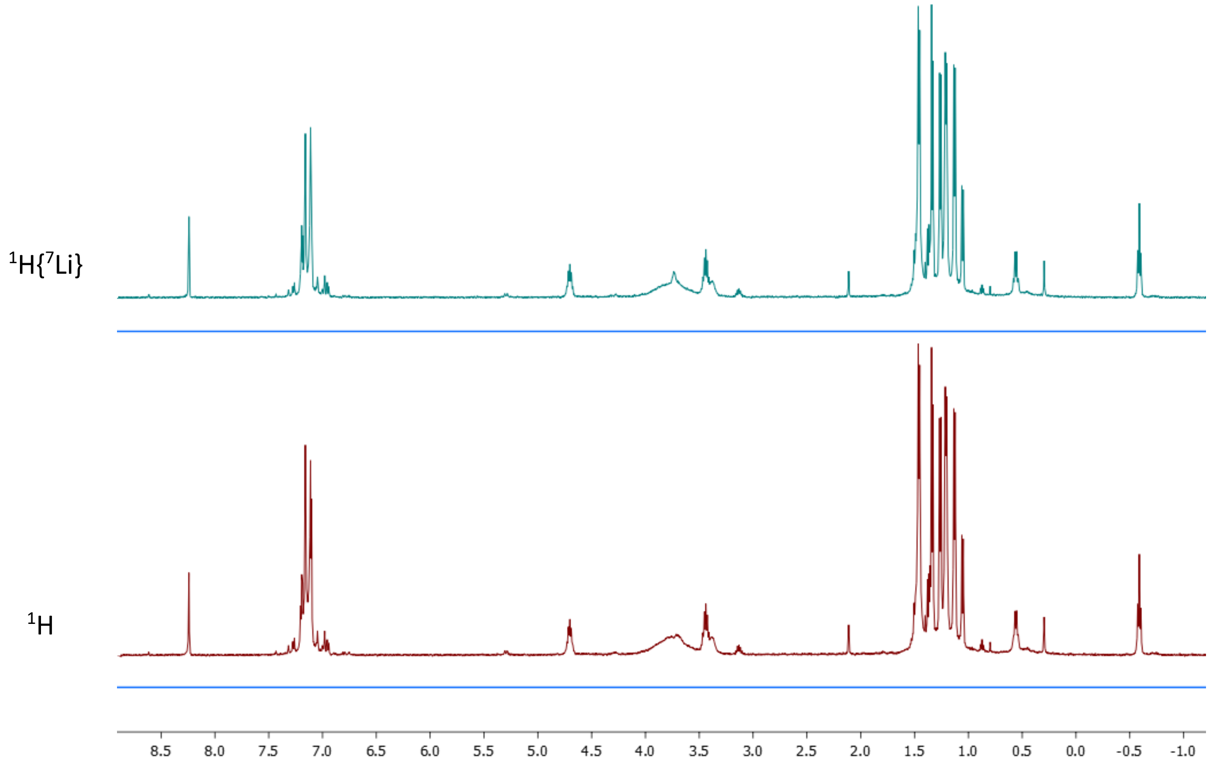

$^1H\{^7Li\}$ NMR (top) and ^1H NMR (bottom) (500 MHz, C_6D_6, 25 °C)

{Mo_2[HLi(thf)CH_2CH_3]$_2$(μ-Ad^{Dipp2})$_2$} (5·LiCH_2CH_3)

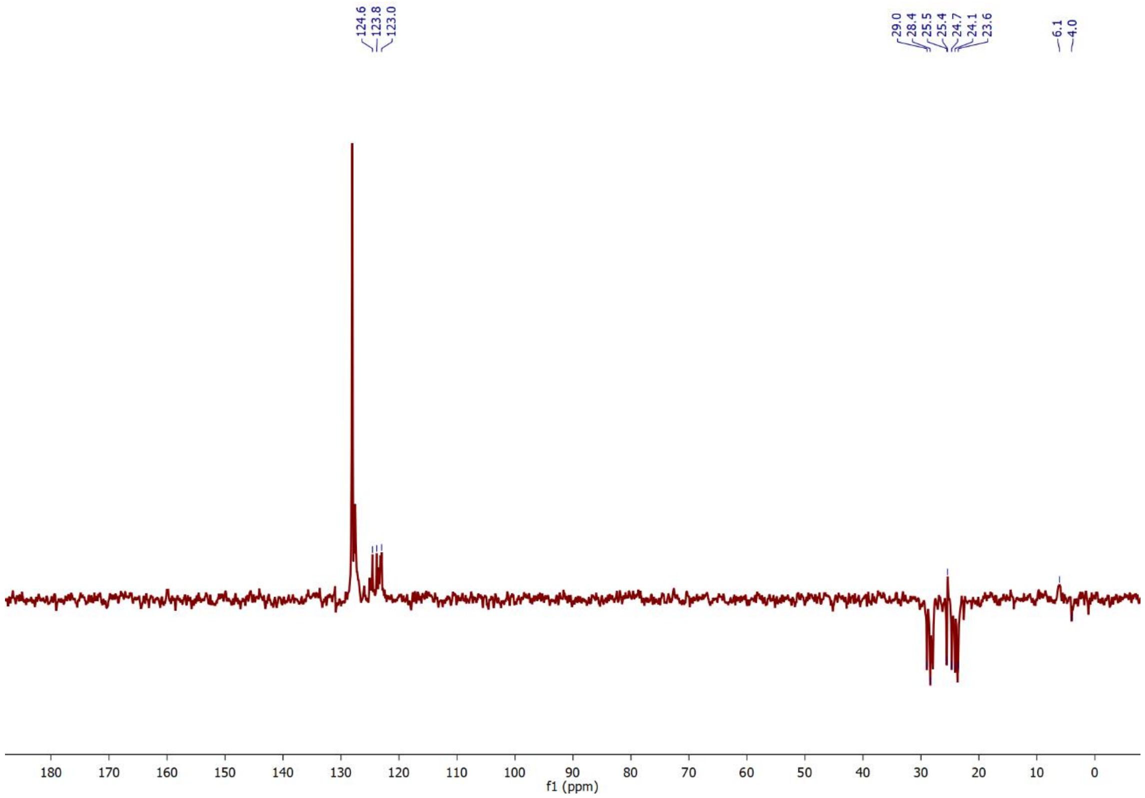

DEPT-135 (125 MHz, C_6D_6, 25 °C)

{Mo_2[HLi(thf)$^{13}CH_2CH_3$]$_2$(μ-Ad^{Dipp2})$_2$} (5·Li$^{13}CH_2CH_3$)

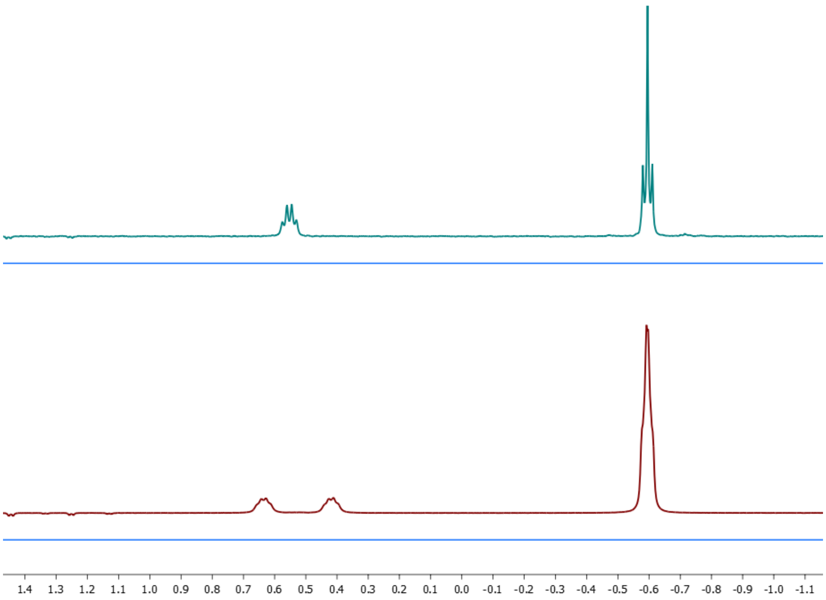

1D-TOCSY experiment (500 MHz, C_6D_6, 25 °C). Top: non labelled sample. Bottom: labelled sample.

{$Mo_2(\mu\text{-}H)[HLi(thf)^{13}CH_2CH_3](\mu\text{-}Ad^{Dipp2})_2$} ($4\cdot Li^{13}CH_2CH_3$)

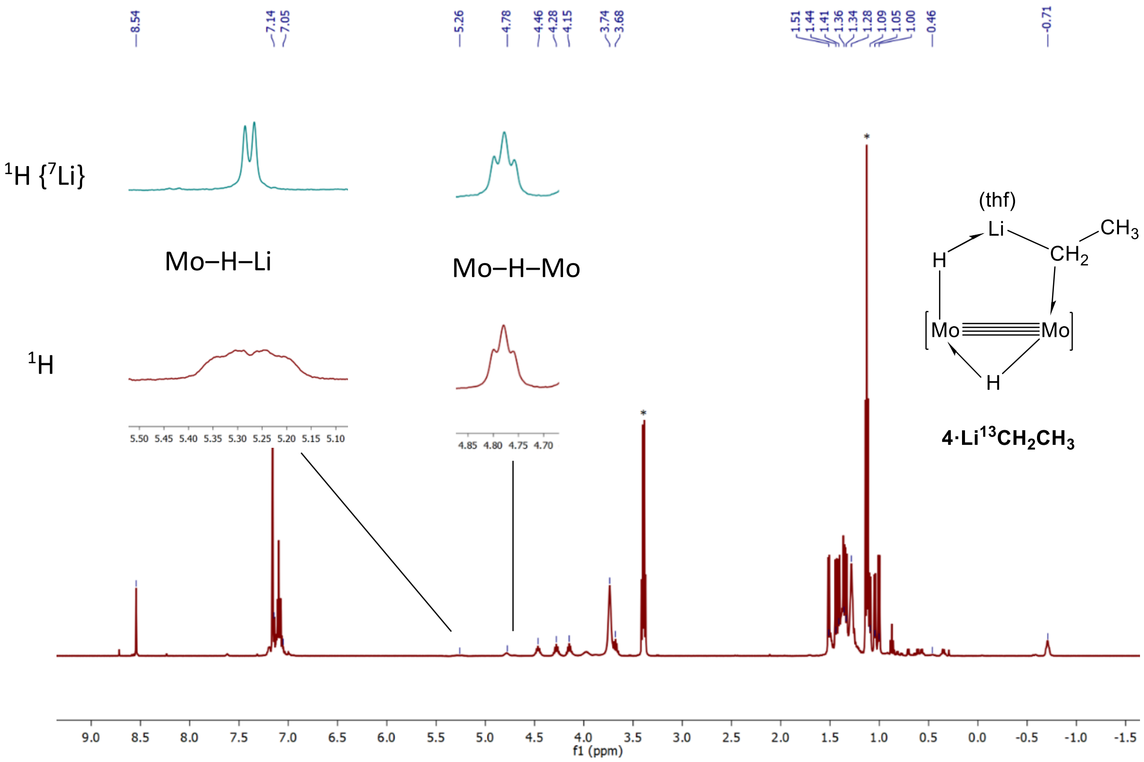

^1H NMR (500 MHz, C_6D_6, 25 °C) *Et_2O. The captions show the Mo–H–Li and Mo–H–Mo resonances.

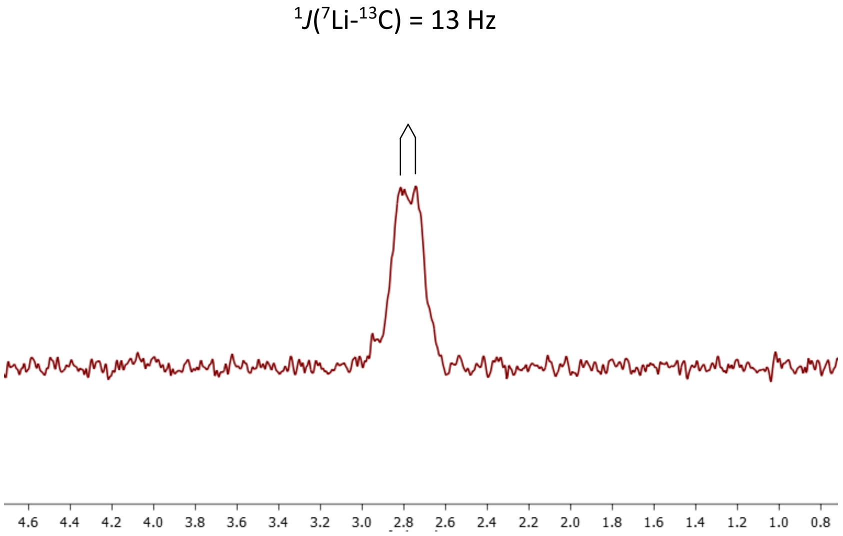

$^7Li\{^1H\}$ NMR (161 MHz, C_6D_6, 25 °C)

{Mo_2(μ-H)[HLi(thf)$^{13}CH_2CH_3$](μ-Ad^{Dipp2})$_2$} (4·Li$^{13}CH_2CH_3$)

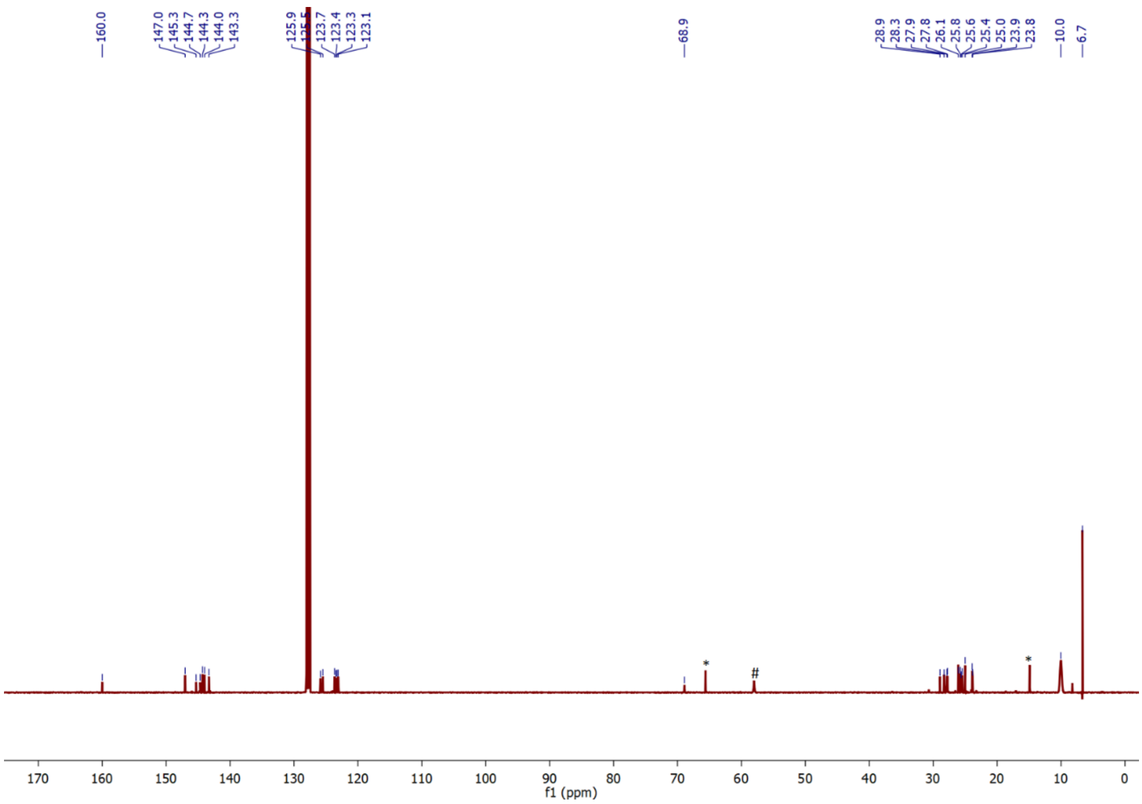

$^{13}C\{^1H\}$ NMR (100 MHz, C_6D_6, 25 °C) *Et_2O #impurity from ($CH_3{}^{13}CH_2$)Li

{$Mo_2(\mu\text{-}O_2CH)[HLi(thf)CH_2CH_3](\mu\text{-}Ad^{Dipp2})_2$} (4·$LiCH_2CH_3 \cdot O_2CH$)

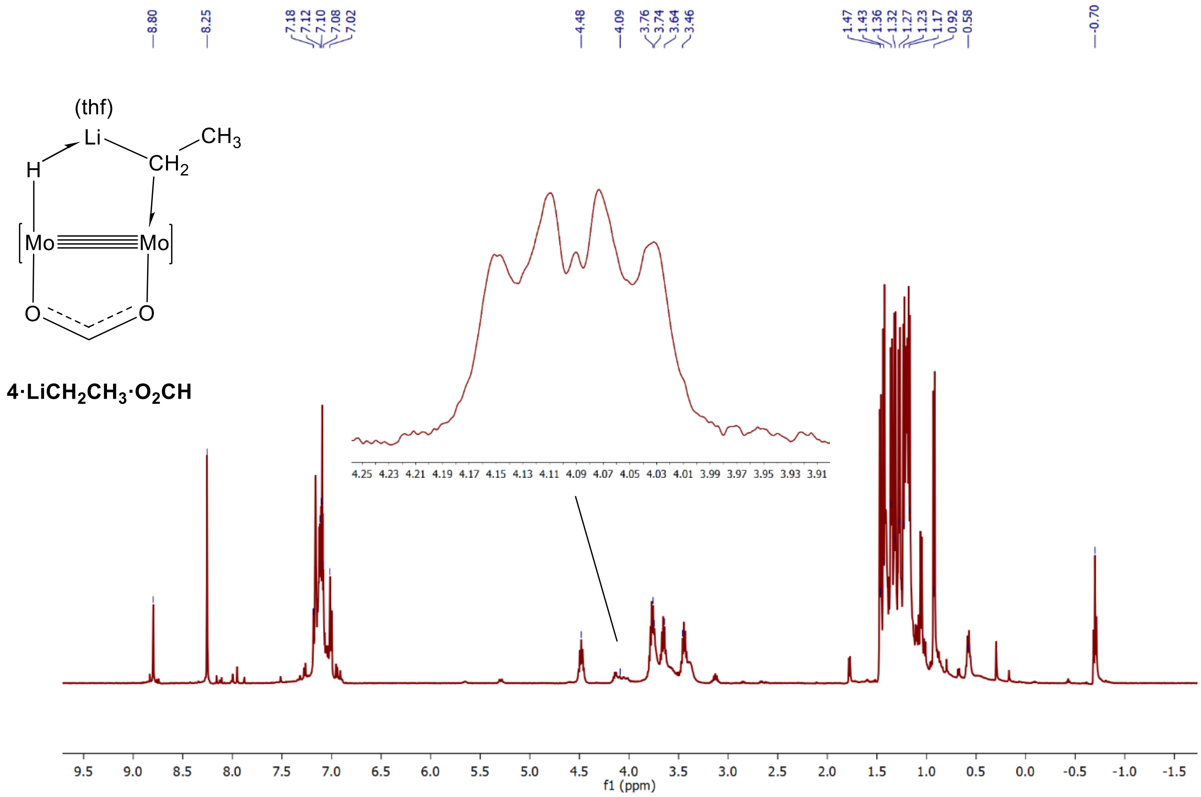

^1H NMR (500 MHz, C_6D_6, 25 °C). The caption shows the Mo–H–Li resonance obtained by a 1D-TOCSY NMR experiment irradiating the CH_2 group of the ethyl fragment.

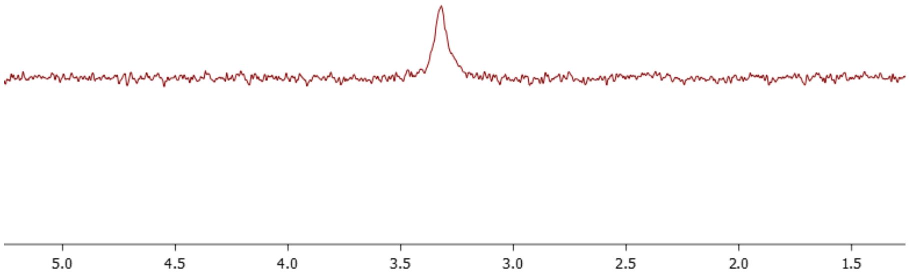

$^7Li\{^1H\}$ NMR (161 MHz, C_6D_6, 25 °C)

{$Mo_2(\mu\text{-}O_2CH)[HLi(thf)CH_2CH_3](\mu\text{-}Ad^{Dipp2})_2$} (4·$LiCH_2CH_3$·$O_2CH$)

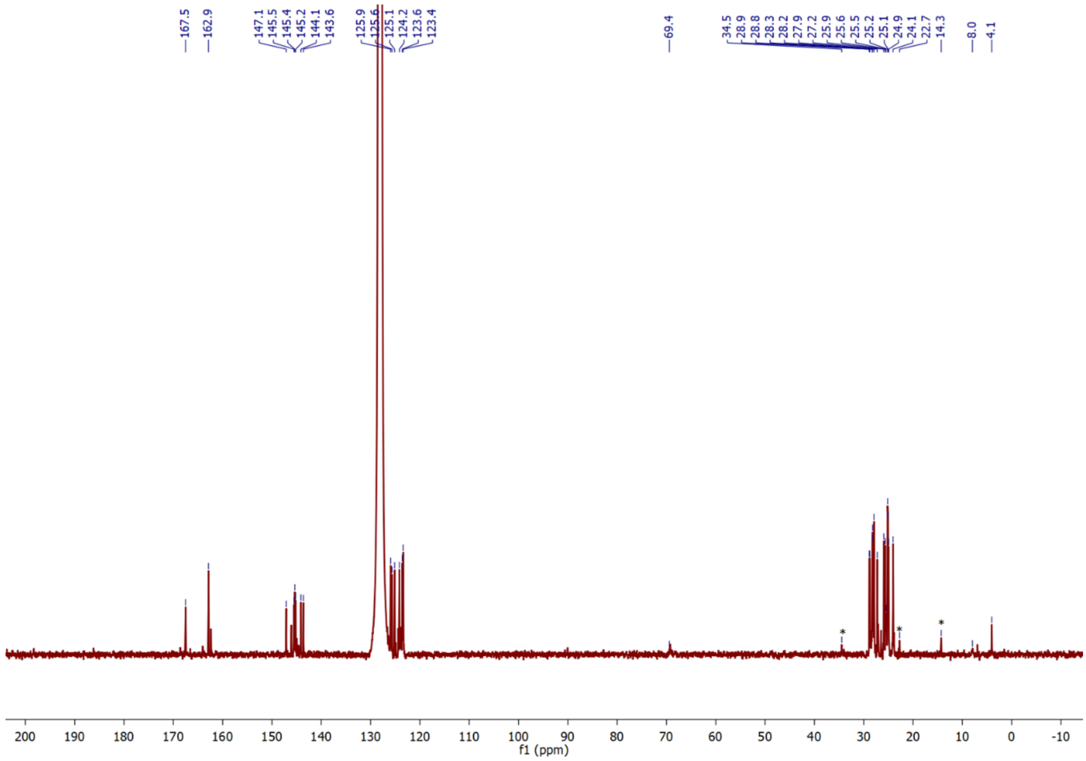

$^{13}C\{^1H\}$ NMR (125 MHz, C_6D_6, 25 °C) *pentane

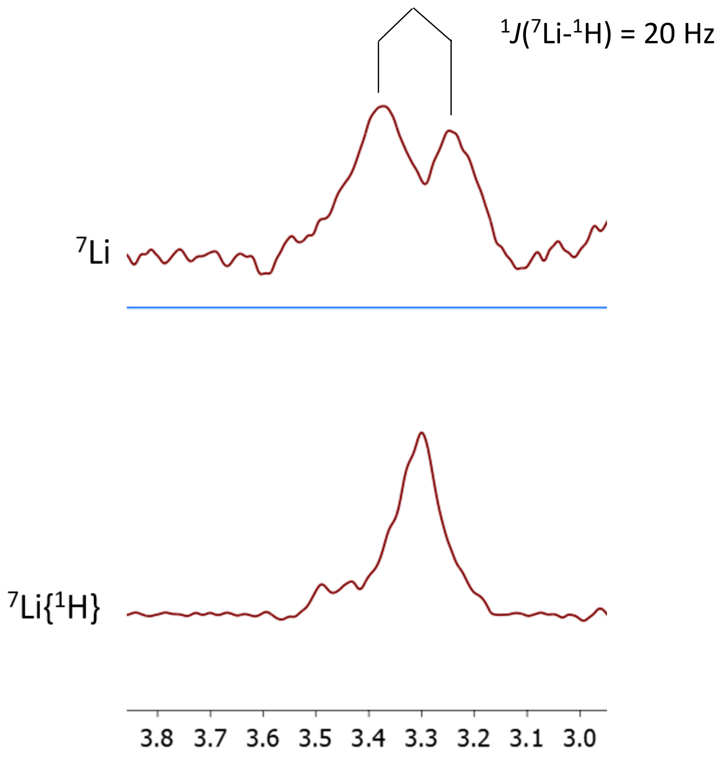

^7Li and $^7Li\{^1H\}$ NMR (161 MHz, C_6D_6, 25 °C)

$\{Mo_2(\mu\text{-}H)[HLi(thf)C_6H_5](\mu\text{-}Ad^{Dipp2})_2\}$ ($4\cdot LiC_6H_5$)

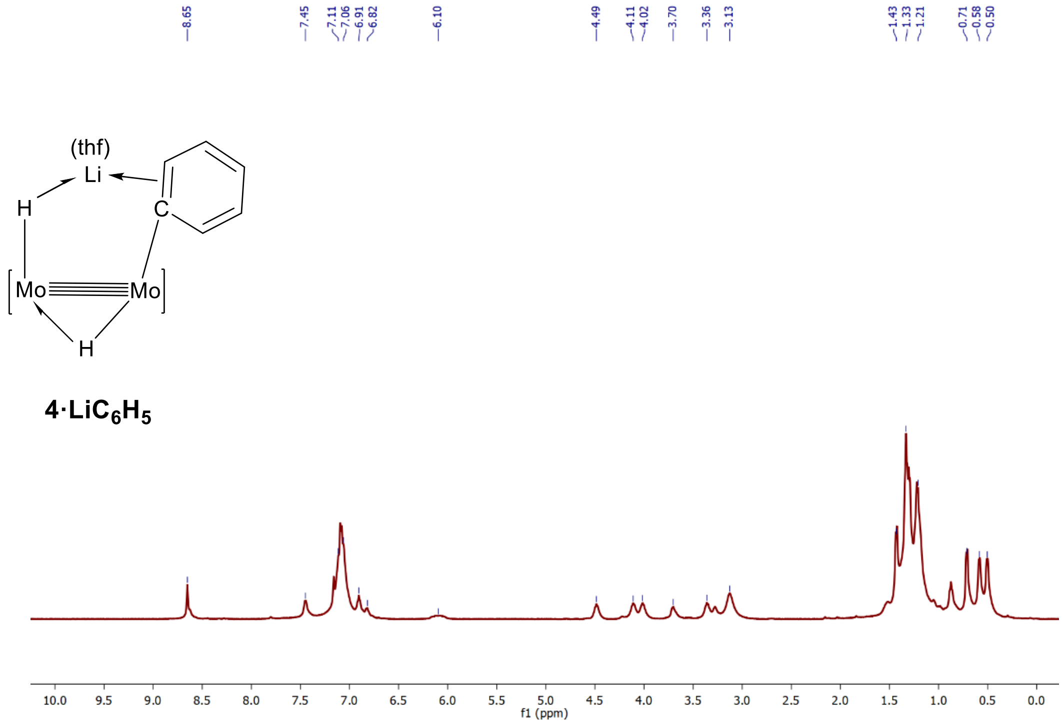

^1H NMR (500 MHz, C_6D_6, 25 °C)

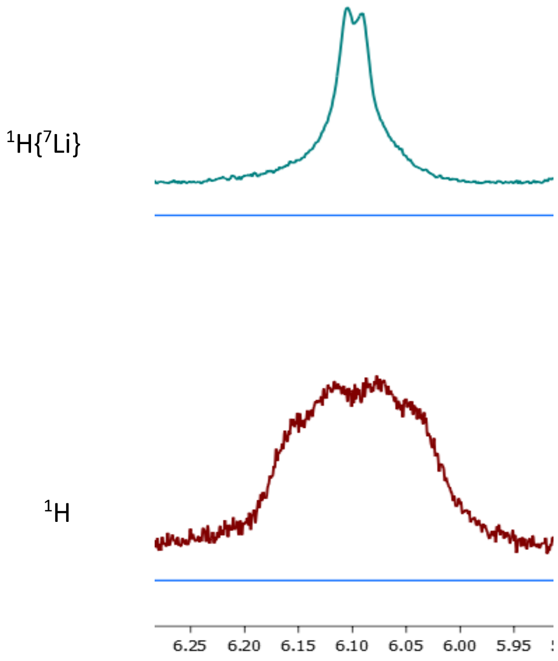

$^1H\{^7Li\}$ NMR (top) and ^1H NMR (bottom) (500 MHz, C_6D_6, 25 °C)

{Mo_2(μ-H)[HLi(thf)C_6H_5](μ-Ad^{Dipp2})$_2$} (4·LiC_6H_5)

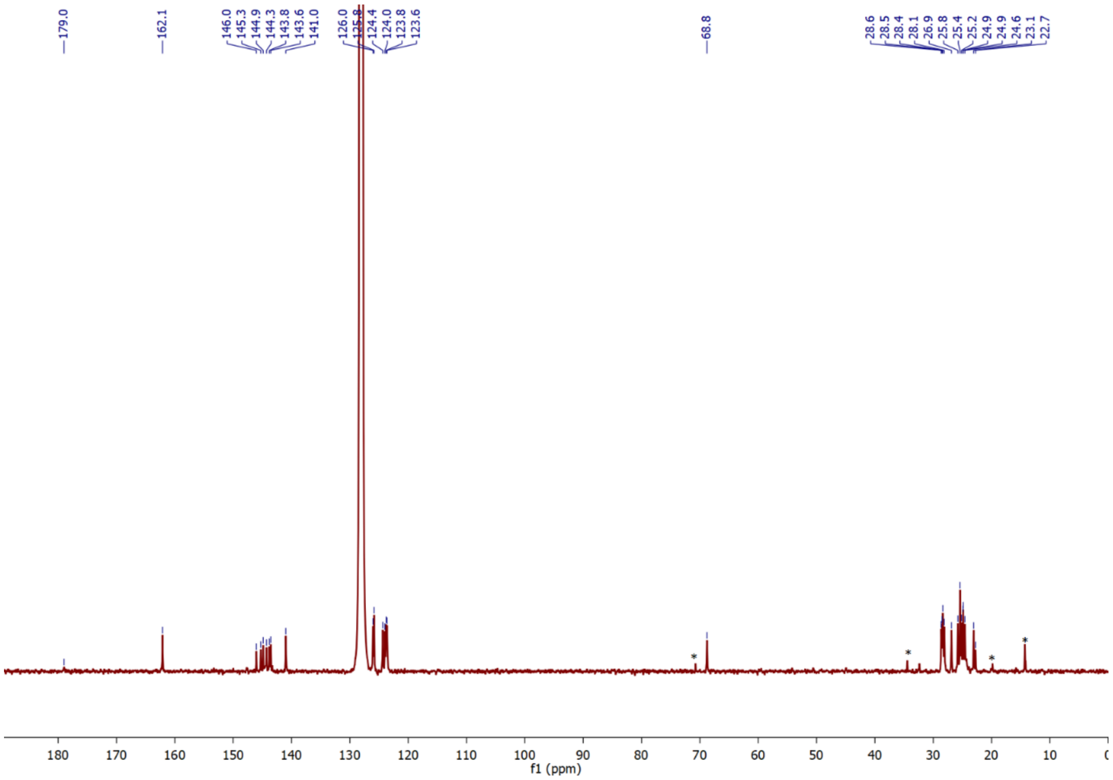

$^{13}C\{^1H\}$ NMR (100 MHz, C_6D_6, 25 °C) *Bu_2O

{Mo_2(μ-H)[HLi(thf)$^{13}C_6H_5$](μ-Ad^{Dipp2})$_2$} (4·$Li^{13}C_6H_5$)

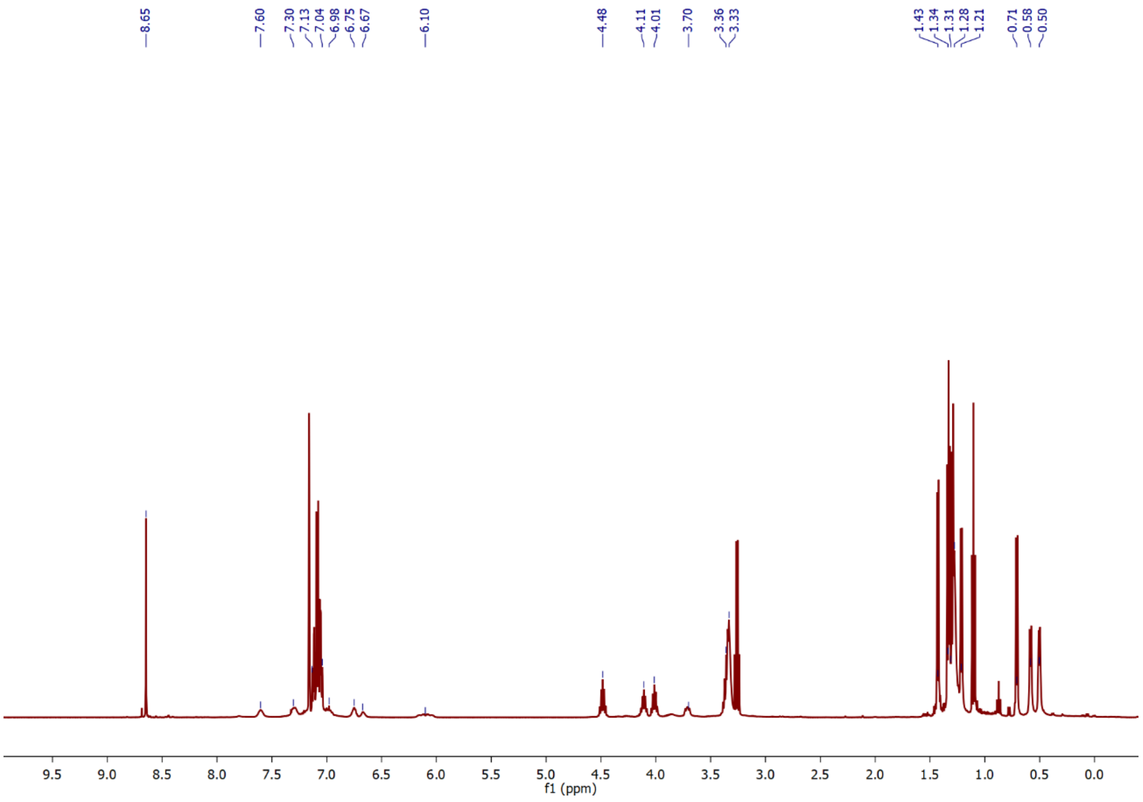

^{1}H NMR (500 MHz, C_6D_6, 25 °C)

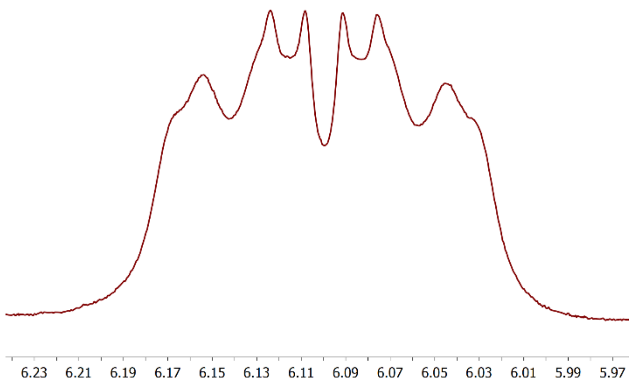

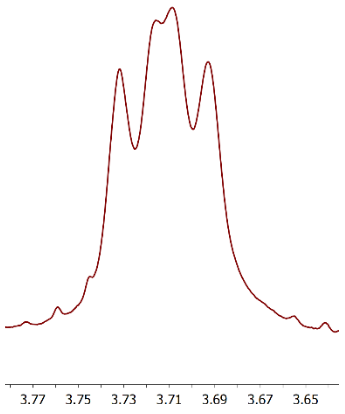

^{1}H NMR (500 MHz, C_6D_6, 25 °C) fragments of the Mo–H–Li and Mo–H–Mo resonances.

Mo$_2$(μ-H)[HLi(thf)$^{13}C_6H_5$](μ-Ad^{Dipp2})$_2$} (4·Li$^{13}C_6H_5$)

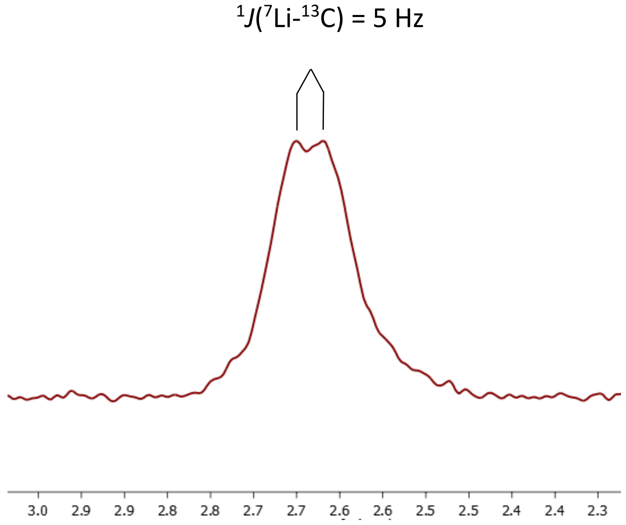

$^7Li\{^1H\}$ NMR (161 MHz, C_6D_6, 25 °C)

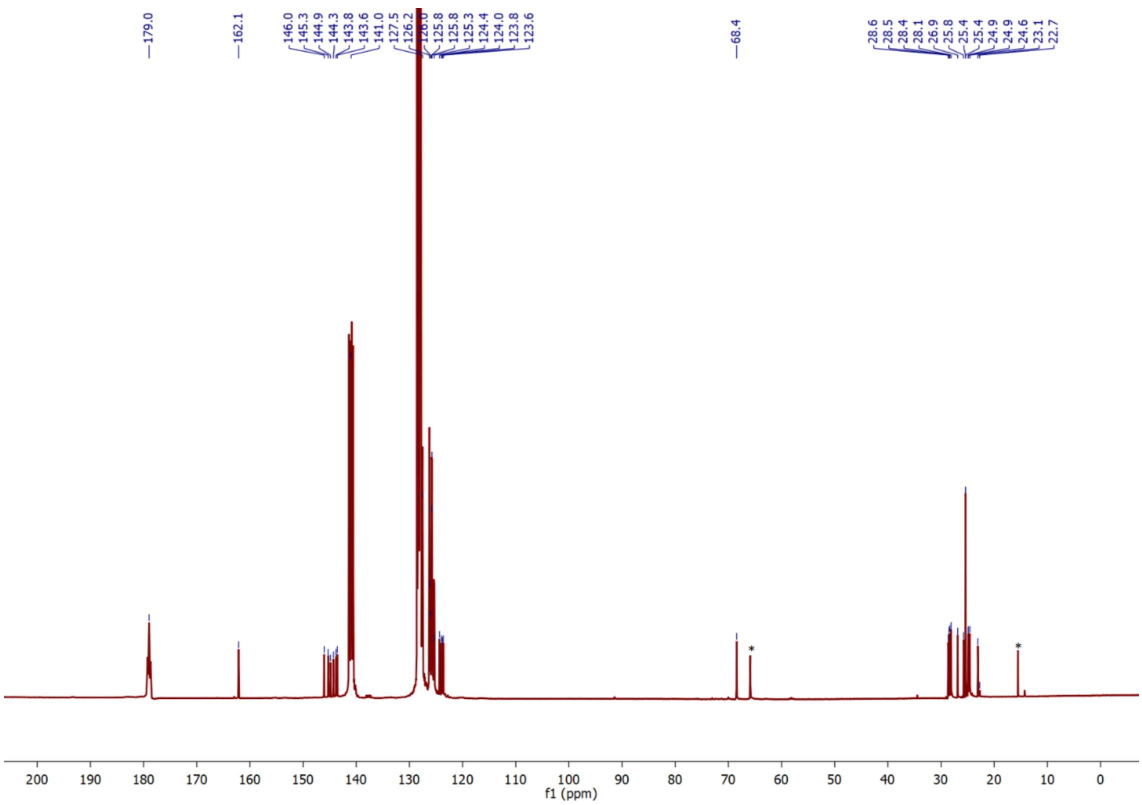

$^{13}C\{^1H\}$ NMR (125 MHz, C_6D_6, 25 °C) *Et_2O

8. IR spectra (Nujol)

5·LiCH₃

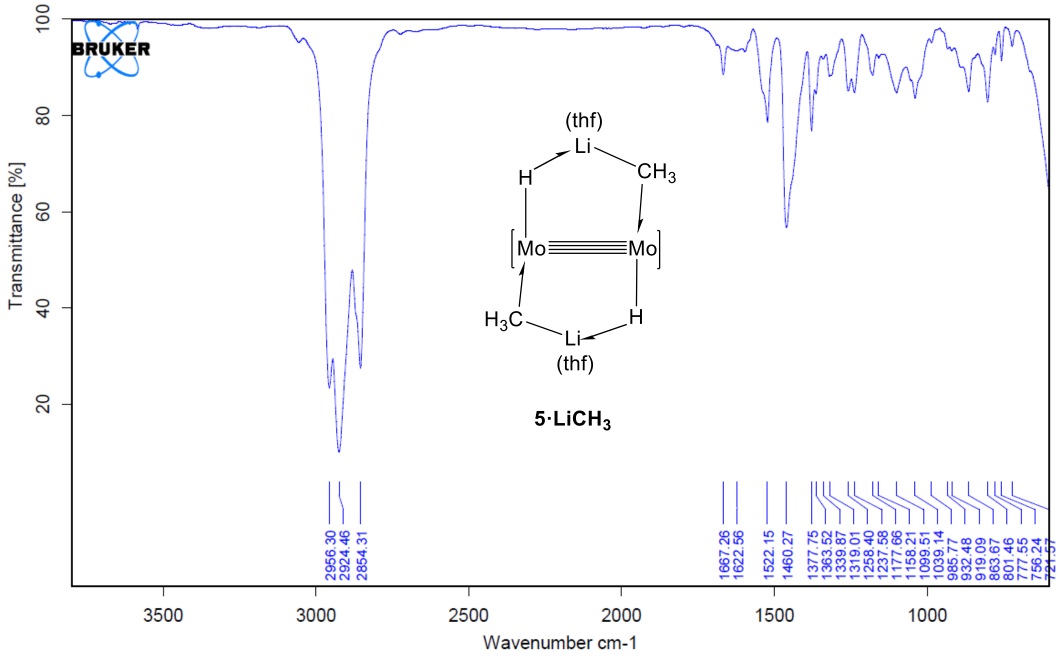

5·LiCH₂CH₃

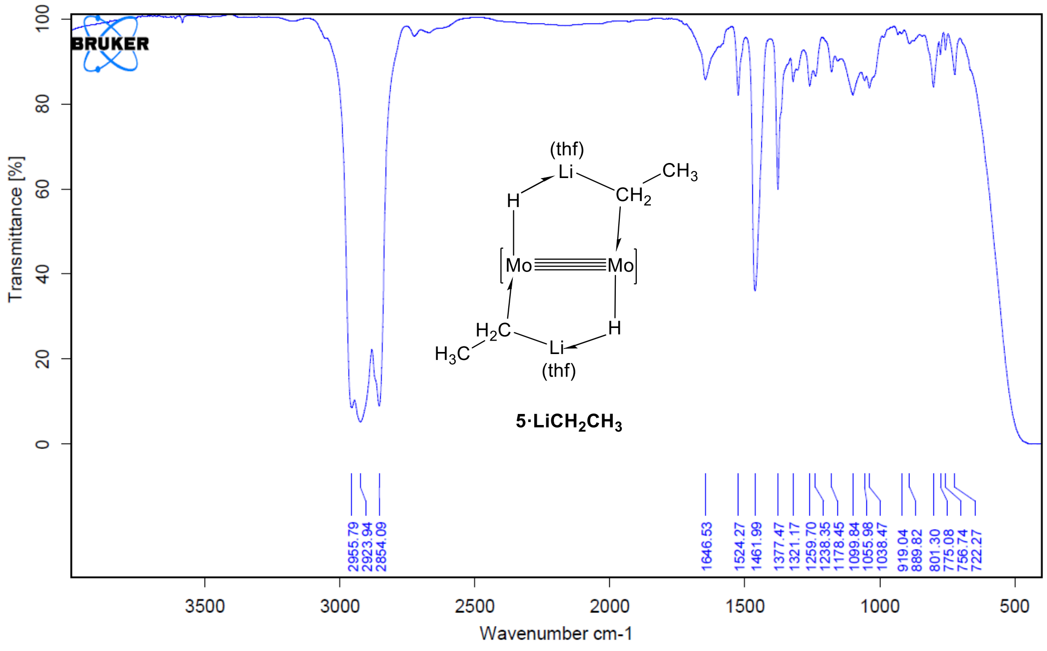

4·LiC_6H_5

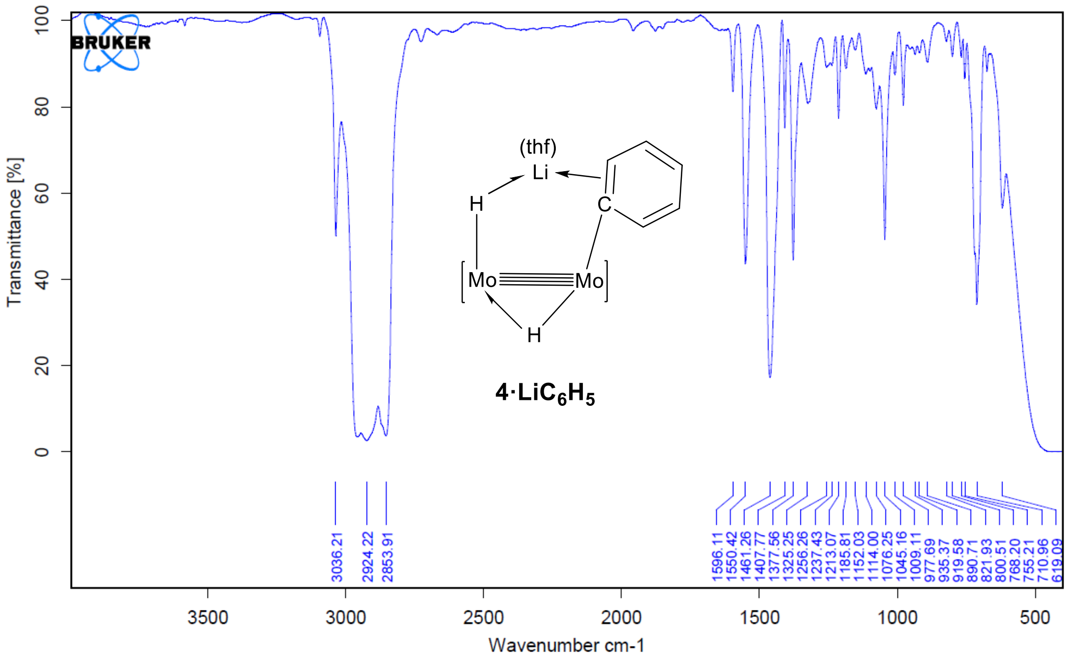

BRUKER
Transmittance [%]
100
80
60
40
20
0
(thf)
Li
H
C
Mo
Mo
H
4·LiC6H5
3036.21
2924.22
2853.91
1596.11
1550.42
1461.26
1407.77
1377.56
1325.25
1256.26
1237.43
1213.07
1185.81
1152.03
1114.00
1076.25
1045.16
1009.11
977.69
935.37
919.58
890.71
821.93
800.51
768.20
755.21
710.96
619.09
3500
3000
2500
2000
1500
1000
500
Wavenumber cm-1

9. Computational details

All the structures have been optimized without any restriction in gas phase employing the Gaussian09 software[20] along with the hybrid B3LYP functional.[21-23] All the light atoms have been described by the Ahlrichs all-electron triple-ζ basis sets,[24,25] in particular: TZV for H, C, N and O atoms, and TZVP for Li. In the case of Mo, the triple-ζ all-electron CGTO basis set, developed by Ahlrichs, was used.[26]

The NBO and NLMO analyses have been carried out at the same level of theory as above with the NBO 7.0 program.[27]

Cartesian coordinates of computed complexes in xyz format

```
146
4·LiMe
Mo   -0.11130100   0.25744800   2.02360300
Mo   -0.10553600   0.58931100  -0.05681300
Li   -0.18008400   3.04313100   1.41846400
C    -2.88538500  -0.12326500   0.91546000
H    -3.93463700  -0.41557300   0.88601700
C    -2.90724500  -0.13700100  -1.48068000
C    -2.95696900  -1.47759200  -1.94319500
C    -3.53352700  -1.73246800  -3.19322300
H    -3.57393800  -2.74965700  -3.55890800
C    -4.06498600  -0.71082700  -3.97026300
H    -4.50208600  -0.93068100  -4.93551700
C    -4.05091600   0.59254200  -3.48838000
H    -4.49519200   1.38037700  -4.08126500
C    -3.48606300   0.90356100  -2.24644200
C    -2.46574300  -2.66180900  -1.10739000
H    -1.94767700  -2.26958700  -0.23466400
C    -3.66147500  -3.51215200  -0.61930200
H    -4.37337300  -2.91066600  -0.05155800
H    -3.31373400  -4.32428600   0.02199700
H    -4.19884600  -3.95484500  -1.46110600
C    -1.45205500  -3.55123300  -1.85524700
H    -1.88714200  -4.01216700  -2.74475200
H    -1.10838200  -4.35433200  -1.19964800
H    -0.57981100  -2.97585600  -2.16171800
C    -3.59375500   2.33113100  -1.71802600
H    -2.93408500   2.40463600  -0.85591400
C    -5.03436800   2.62025000  -1.23474800
H    -5.74152400   2.57085000  -2.06584100
H    -5.09867200   3.61932900  -0.79629600
H    -5.35337800   1.89793100  -0.48287900
C    -3.15619100   3.39448800  -2.74602000
H    -2.15528900   3.19341700  -3.12672100
H    -3.14862700   4.38419100  -2.28309000
H    -3.83946800   3.43907700  -3.59661600
C    -3.00088000  -0.24413500   3.31191400
C    -3.74893700   0.84440000   3.82599600
C    -4.45475800   0.67156500   5.02117300
H    -5.03235000   1.49558000   5.41884800
C    -4.42702900  -0.53473500   5.71099400
H    -4.98006000  -0.64857300   6.63410300
C    -3.68312500  -1.59250700   5.20457800
H    -3.66020200  -2.52973200   5.74396900
C    -2.96225500  -1.47455900   4.00993200
C    -3.80368500   2.20478100   3.13244300
H    -3.18084500   2.14713900   2.24161400
C    -5.23826800   2.56412200   2.68824900
H    -5.66129700   1.78909000   2.04793900
H    -5.24236500   3.50408000   2.13163600
H    -5.90107100   2.68295500   3.54792000
C    -3.21678900   3.31855200   4.02735200
H    -3.80477500   3.45102400   4.93785500
H    -3.21430100   4.27137800   3.49073000
H    -2.19244200   3.07929500   4.31360200
C    -2.16656900  -2.68011400   3.51456900
H    -1.69573400  -2.40693800   2.57027100
C    -3.07744700  -3.89827600   3.24688700
H    -3.53307600  -4.26646400   4.16858600
H    -2.49640100  -4.71595300   2.81441500
H    -3.88067100  -3.64731100   2.55361600
C    -1.04332000  -3.05572400   4.50609700
H    -0.35137600  -2.22558200   4.65933100
H    -0.47312000  -3.90655700   4.12963100
H    -1.45016300  -3.33355900   5.48069800
N    -2.23969000  -0.07584000   2.08934500
N    -2.23549700   0.16257100  -0.22515500
H     0.02424000  -1.27983100   0.67934300
C     2.71903600   0.27657500   0.99176600
H     3.79779300   0.13431000   0.97155900
C     2.77380600  -0.47335400   3.25905900
C     2.99967000   0.30047500   4.42290900
C     3.66893500  -0.28696600   5.50215000
H     3.84630900   0.29652800   6.39585500
C     4.11875800  -1.60051900   5.44781400
H     4.63749100  -2.03508800   6.29221200
C     3.89891100  -2.35096500   4.29919000
H     4.25141500  -3.37277800   4.26069500
C     3.22686500  -1.81799000   3.19310900
C     2.56964000   1.76175500   4.52979300
H     2.04396000   2.01137600   3.61038800
C     3.79054800   2.70037700   4.65412900
H     4.48075100   2.56253900   3.82094000
H     3.47024500   3.74608200   4.66250900
```

H	4.34328100	2.51800800	5.57817100
C	1.58430300	1.98710100	5.69584200
H	2.03998800	1.75047500	6.65961600
H	1.26371200	3.03153100	5.72927500
H	0.69391300	1.36897900	5.57951100
C	3.01774400	-2.70738800	1.96403700
H	2.35480300	-2.18523700	1.27661800
C	4.35213700	-2.98192300	1.23350900
H	5.04762300	-3.52561200	1.87708000
H	4.17843900	-3.58767000	0.34136200
H	4.84108600	-2.05661500	0.92539700
C	2.32219600	-4.04008300	2.31408000
H	1.37290100	-3.86358500	2.81770100
H	2.12115400	-4.60411700	1.40078200
H	2.93953700	-4.66613300	2.96150700
C	2.89304500	0.96152800	-1.28209000
C	2.83122200	0.18534700	-2.46598700
C	3.64421600	0.53744800	-3.54893100
H	3.60773900	-0.05882000	-4.45057500
C	4.50196900	1.62795200	-3.49131000
H	5.12810500	1.87755600	-4.33768100
C	4.54296600	2.39749200	-2.33634600
H	5.20383700	3.25306700	-2.29670800
C	3.74621300	2.09718000	-1.22544600
C	1.92839000	-1.03808000	-2.61219500
H	1.35279400	-1.14394400	-1.69130400
C	2.74959200	-2.33612700	-2.77623900
H	3.43839800	-2.47292500	-1.94164100
H	2.08593600	-3.20264000	-2.81583300
H	3.33674700	-2.32198600	-3.69708800
C	0.93198400	-0.86737000	-3.77993300
H	1.45017700	-0.78492400	-4.73731700
H	0.26000300	-1.72419900	-3.84006600
H	0.32218700	0.02884000	-3.65412300
C	3.83383900	3.02808800	-0.01180200
H	3.06061000	2.73532700	0.69695000
C	5.19915800	2.91224400	0.70313700
H	6.01435700	3.19214000	0.03272800
H	5.23621800	3.57534000	1.57050600
H	5.38840900	1.89469800	1.04661000
C	3.56530600	4.50191200	-0.39417100
H	2.62288400	4.60428900	-0.93313700
H	3.51991900	5.11971100	0.50643300
H	4.35717200	4.90442400	-1.02822200
C	-0.15488000	2.78071000	-0.74488800
O	-0.06434100	4.86661100	1.91561400
C	-0.20239600	6.05317600	1.02756000
H	-1.15490600	5.97401500	0.50927900
H	0.60774300	6.01537500	0.30132400
C	-0.10289200	7.27108900	1.95409300
H	0.35100900	8.12504300	1.45379200
H	-1.09208700	7.56933000	2.30461400
C	0.74806800	6.75278100	3.13261900
H	0.60698800	7.33849500	4.03969800
H	1.80820400	6.76894700	2.87550200
C	0.25816200	5.31445800	3.29526400
H	0.99938100	4.62774600	3.69324800
H	-0.65088600	5.25484800	3.89358000
N	2.07563800	0.60523000	-0.13973300
N	2.05079000	0.09795600	2.13896200
H	-0.21792800	1.90582700	2.81618300
H	0.75757400	3.37172700	-0.59209800
H	-1.02138500	3.43431000	-0.56759000
H	-0.17792400	2.57170900	-1.82221000

164

5·LiMe

Mo	0.99330100	-0.29298100	0.16916800
Mo	-1.03674900	0.34069100	-0.10597300
Li	-1.07922900	-2.32332300	0.97905200
Li	1.04435000	2.40329400	-0.84563700
C	0.01133800	-1.04105600	-2.63247400
H	0.02426800	-1.44345600	-3.64401100
C	-2.28266900	-0.54613000	-3.03939800
C	-2.78880600	0.63830800	-3.63548900
C	-3.94684600	0.57318500	-4.41815700
H	-4.33099900	1.47971100	-4.86637000
C	-4.60313200	-0.62753100	-4.64736400
H	-5.49726100	-0.65865100	-5.25609200
C	-4.08322200	-1.79209200	-4.10083600
H	-4.57906700	-2.73189200	-4.30198900
C	-2.93228200	-1.78438800	-3.30297500
C	-2.09081900	1.98971200	-3.51318100
H	-1.25189800	1.86617100	-2.83317600
C	-1.54559900	2.46018200	-4.88016700
H	-0.88987100	1.71120400	-5.32314900
H	-0.97656000	3.38754700	-4.76706400
H	-2.35563000	2.65407200	-5.58710000
C	-3.01957400	3.05820900	-2.90539900
H	-3.87551500	3.26435300	-3.55168600
H	-2.48112700	3.99772500	-2.76060500
H	-3.39744100	2.73616000	-1.93689800
C	-2.41689300	-3.13728900	-2.80834000
H	-1.63951400	-2.95264000	-2.06829700
C	-1.79619800	-3.94636100	-3.97258000
H	-2.54263900	-4.13543300	-4.74673200
H	-1.42664500	-4.91121600	-3.61910800
H	-0.96567600	-3.41550100	-4.43862700
C	-3.51519700	-3.98780600	-2.13060700
H	-4.03948600	-3.42351000	-1.35970900
H	-3.06839800	-4.87482400	-1.67401400
H	-4.25672700	-4.33529900	-2.85188500
C	2.32510300	-1.59335300	-2.56315100
C	2.92843100	-2.82122800	-2.17171200
C	4.10579400	-3.23363700	-2.80358100
H	4.56677000	-4.16328300	-2.50545300
C	4.69081300	-2.48587900	-3.81785600
H	5.60138600	-2.82583400	-4.29362900
C	4.08569300	-1.30584200	-4.21923000
H	4.53125300	-0.73195800	-5.02023100
C	2.91085400	-0.83726000	-3.61549100
C	2.27866200	-3.75046900	-1.14837300
H	1.75887000	-3.13007100	-0.41967500
C	1.21942600	-4.63947200	-1.83813200
H	0.45760400	-4.03522800	-2.32400900
H	0.72482700	-5.28909700	-1.10991000
H	1.68269100	-5.27549900	-2.59656300
C	3.27950800	-4.64026000	-0.38604500
H	3.71008500	-5.41457400	-1.02519000
H	2.76904400	-5.15003600	0.43409800
H	4.09696300	-4.05914000	0.04022600
C	2.31588200	0.46183600	-4.16622700
H	1.52648900	0.78732800	-3.49007000
C	1.69221300	0.23817000	-5.56430600
H	2.44477900	-0.12804200	-6.26572300
H	1.29171800	1.17354100	-5.96055200

H	0.88386200	-0.49268500	-5.53594400
C	3.35616500	1.60218500	-4.25292700
H	3.88957800	1.72674300	-3.31096500
H	2.85662900	2.54213100	-4.50138500
H	4.09666600	1.41279900	-5.03208500
C	2.71209800	1.15880400	-0.39922900
H	3.42746200	3.89757800	-2.22294000
H	0.68527600	6.97007400	-3.05908000
N	1.12581500	-1.11965000	-1.88736300
N	-1.11548200	-0.47676400	-2.17189500
H	-0.69454200	2.03937000	-0.72626100
H	2.90802800	1.46142200	-1.43636700
H	2.95988900	2.00197000	0.25817400
H	3.47904300	0.40128300	-0.18669500
C	-0.05648800	1.07584600	2.70247700
H	-0.07090300	1.47109600	3.71639000
C	2.27377900	0.76700900	3.03907900
C	2.94720900	-0.35425200	3.58836400
C	4.11497900	-0.15574200	4.33271400
H	4.62800800	-1.01397300	4.74589300
C	4.62135500	1.11481100	4.56803000
H	5.52436000	1.24800500	5.14924800
C	3.94318700	2.21361400	4.05972200
H	4.32660100	3.20540900	4.25758600
C	2.77670400	2.07323100	3.29816200
C	2.42471600	-1.78058900	3.44798600
H	1.53255100	-1.74514100	2.82772600
C	2.03310900	-2.37259000	4.82046100
H	1.31059500	-1.73735100	5.33289200
H	1.58907700	-3.36442700	4.69569200
H	2.90220900	-2.48203600	5.47288200
C	3.44072300	-2.69462400	2.73604600
H	4.36574500	-2.79292100	3.30870800
H	3.02500100	-3.69540300	2.60176500
H	3.69431400	-2.30168800	1.75159700
C	2.09732500	3.35775500	2.81531900
H	1.31194200	3.08189000	2.11285600
C	1.44404800	4.11978400	3.99269000
H	2.19735800	4.40320900	4.73053000
H	0.95918900	5.03259100	3.64019600
H	0.69479600	3.51334300	4.50206800
C	3.07027000	4.30284500	2.07484400
H	3.60508900	3.78081600	1.28178900
H	2.51527500	5.13471900	1.63408400
H	3.81193700	4.72831800	2.75337800
C	-2.39688700	1.43706800	2.71686000
C	-3.16532900	2.57493400	2.34917000
C	-4.33111100	2.86834400	3.06361100
H	-4.91776800	3.73308300	2.79038300
C	-4.75131700	2.08297800	4.13014700
H	-5.65465300	2.33271900	4.67115200
C	-3.99670000	0.97864900	4.49281700
H	-4.32206900	0.37025700	5.32590100
C	-2.82604900	0.62932700	3.80690600
C	-2.71010100	3.53375100	1.25167000
H	-2.14147200	2.95914400	0.52115500
C	-1.75322000	4.59857800	1.83404500
H	-0.88537100	4.13477500	2.29849300
H	-1.40082500	5.27067000	1.04628800
H	-2.26028800	5.20332200	2.59019700
C	-3.86880600	4.22652000	0.50966800
H	-4.38782400	4.94994400	1.14196000
H	-3.48021200	4.77372400	-0.35091500
H	-4.60333000	3.50620000	0.14682500
C	-2.07856300	-0.61578100	4.29737700
H	-1.29093900	-0.84620800	3.58090700
C	-1.41600900	-0.37025700	5.67311200
H	-2.16704700	-0.11110500	6.42219800
H	-0.89774700	-1.26831400	6.01582300
H	-0.69283600	0.44461200	5.63499700
C	-2.99653100	-1.85630400	4.38728000
H	-3.53134100	-2.02196400	3.45246200
H	-2.39946700	-2.74434900	4.60922000
H	-3.73746600	-1.75261300	5.18181000
C	-2.75038200	-1.12894800	0.44002300
O	-1.44606700	-4.06499600	1.64862200
C	-2.75766000	-4.75157100	1.75491700
H	-3.33820500	-4.47969300	0.87894700
H	-3.26157900	-4.39175100	2.65203600
C	-2.41118500	-6.23665600	1.85124700
H	-3.19343200	-6.80907500	2.34773000
H	-2.25657000	-6.65796900	0.85671300
C	-1.08873300	-6.22566600	2.64750000
H	-0.49940900	-7.12790700	2.49159200
H	-1.29203500	-6.13381700	3.71545400
C	-0.36964600	-4.97576700	2.12462800
H	0.20212500	-4.44856200	2.88384700
H	0.27348000	-5.19047500	1.27344500
N	-1.18896400	1.10149900	1.98271700
N	1.08852300	0.57297100	2.22016200
H	0.65706700	-1.96359300	0.85643800
H	-3.00165600	-1.33106700	1.48964800
H	-2.93547500	-2.04253100	-0.14005200
H	-3.52325200	-0.42569900	0.10086000
O	1.47243400	4.12946200	-1.52915000
C	2.81923200	4.69364900	-1.80533000
H	3.24914400	5.02317000	-0.86021100
C	2.56769400	5.86266200	-2.75686600
H	2.51057100	5.51131400	-3.78830800
H	3.35177100	6.61595700	-2.69528700
C	1.19616300	6.39338300	-2.28950300
H	1.31552300	7.02917400	-1.41091100
C	0.43181300	5.11452400	-1.93111200
H	-0.10019600	4.69947300	-2.78544500
H	-0.25607300	5.22748800	-1.09761800

149

4·LiEt

Mo	-0.50689700	0.88794100	-0.04517400
Mo	0.28989500	-1.06510700	-0.06574600
Li	2.35329300	0.91992300	0.01053300
O	4.00593000	1.80731300	0.26296200
C	5.58350700	3.55311200	0.73230500
H	5.89921500	4.22629100	1.52783300
H	5.76712900	4.04637300	-0.22345000
H	3.14374000	-0.89862700	-0.85621300
H	3.06585100	-0.84959600	0.91063800
H	0.92444800	2.02485500	0.01412000
N	-0.85664700	0.88276300	2.09306000
N	0.14534900	-1.23081300	2.12193600
C	-0.47698400	-0.21939400	2.75153100
H	-0.68763900	-0.30464900	3.81600900
C	0.67841100	-2.27258600	2.98240100
C	1.73300900	-1.97143900	3.88619400
C	-3.03367100	1.46672500	3.13662600
C	-1.70541100	1.83092400	2.78940600

C	2.39451900	-0.59317200	3.97634200
H	1.99847100	0.02251000	3.17016200
C	0.15275700	-3.58686600	2.92570000
C	-3.62042600	0.08145700	2.85251600
H	-2.91391200	-0.46761700	2.23249500
C	0.21370000	3.55756100	2.78946100
H	0.70907000	2.71906000	2.30452800
C	0.66555300	-4.55689100	3.79485300
H	0.25815600	-5.55819500	3.76613900
C	-1.21882500	3.12538100	3.09471700
C	-0.96957900	-3.99438700	1.97274800
H	-1.17773100	-3.14842300	1.31597000
C	3.92603700	-0.67351700	3.78253800
H	4.40831500	-1.20783400	4.60287300
H	4.35283500	0.33245000	3.75129300
H	4.18109700	-1.18560300	2.85442700
C	0.99026300	3.89239900	4.08262800
H	0.56731500	4.76190400	4.59020700
H	2.03429600	4.12164800	3.85160100
H	0.97146900	3.05639600	4.78263500
C	-3.84130200	2.41338900	3.77756200
H	-4.85330900	2.14562200	4.04965200
C	1.67780900	-4.26398900	4.69893800
H	2.05390900	-5.02857900	5.36578600
C	2.07124800	0.11115300	5.31358900
H	0.99706600	0.23827100	5.45163100
H	2.53702900	1.09842900	5.34869600
H	2.44776400	-0.46672100	6.16017800
C	2.20552500	-2.98020300	4.73328300
H	3.00139800	-2.75534700	5.43066000
C	0.25610000	4.74614200	1.80590800
H	-0.23332100	4.49304600	0.86542200
H	1.29079200	5.01926700	1.58322300
H	-0.23769100	5.62772800	2.22029100
C	-4.94011100	0.15424900	2.05549000
H	-5.73656100	0.63498100	2.62727300
H	-5.27601700	-0.85349500	1.80168900
H	-4.80567500	0.70935600	1.12866400
C	-3.83561300	-0.71259500	4.16157800
H	-2.90434500	-0.83620600	4.71676700
H	-4.23546100	-1.70493700	3.94310500
H	-4.54584900	-0.20304200	4.81666600
C	-2.07374500	4.03444400	3.72759700
H	-1.70990000	5.02573100	3.96314400
C	-3.37548600	3.68918200	4.06927600
H	-4.01934800	4.40602400	4.56173400
C	-0.56007000	-5.19726200	1.09499100
H	0.33506700	-4.97976200	0.51070200
H	-1.36128500	-5.45437400	0.40154100
H	-0.35696400	-6.08257300	1.70026200
C	-2.27586900	-4.30122800	2.73858400
H	-2.15402300	-5.16268200	3.39934100
H	-3.08253100	-4.52674000	2.03751700
H	-2.58241600	-3.45157900	3.34886700
H	-1.70586200	-0.76866700	-0.08684100
N	0.12012800	-1.18520400	-2.24504200
N	-0.66401700	1.00440900	-2.18670800
C	-0.36704300	-0.10565300	-2.87820200
H	-0.54167400	-0.13630400	-3.95322300
C	-1.09880200	2.18664800	-2.90769300
C	-2.47979800	2.46501300	-3.04766200
C	1.50526900	-2.75525400	-3.59859600
C	0.26377200	-2.41683600	-3.00583500
C	2.77985000	-2.80496500	0.07849600
H	3.83900600	-3.09329300	0.07759500
H	2.34522700	-3.22275800	0.98764200
H	2.31625800	-3.31103900	-0.77193500
C	2.58056000	-1.27054300	0.01596000
C	-3.56857900	1.53429500	-2.51811100
H	-3.08289200	0.67116200	-2.06420700
C	-0.13506200	3.08301200	-3.43351200
C	2.65690700	-1.75507400	-3.68460300
H	2.47145900	-0.97943300	-2.94296200
C	-2.25655000	-2.88230800	-2.69467100
H	-2.17679200	-1.97799300	-2.09420000
C	-0.57493000	4.24153600	-4.08243300
H	0.15403500	4.93022000	-4.48815000
C	-0.84732900	-3.28950800	-3.12751900
C	1.36820600	2.83458100	-3.32265000
H	1.50659600	1.89475700	-2.79061800
C	-4.41533600	2.22073100	-1.42466700
H	-4.94099500	3.09328000	-1.81832100
H	-5.16435500	1.52828400	-1.03585500
H	-3.79575300	2.55443700	-0.59072600
C	-3.11882800	-2.56521400	-3.93907800
H	-3.24412800	-3.45315400	-4.56313900
H	-4.11015400	-2.22059000	-3.63819100
H	-2.66096900	-1.78940600	-4.55452900
C	1.63650300	-4.00434600	-4.21603700
H	2.58139200	-4.27798100	-4.66472500
C	-1.92822700	4.52690600	-4.21722700
H	-2.24862700	5.42916700	-4.72140100
C	-4.47822900	1.01905200	-3.65562400
H	-3.89635100	0.53490100	-4.44056500
H	-5.19565500	0.29323900	-3.26670300
H	-5.04449400	1.83332600	-4.11277500
C	-2.86568800	3.64057400	-3.70364900
H	-3.91862400	3.86182500	-3.81462900
C	-2.96299600	-3.93456100	-1.81869400
H	-2.38464100	-4.14754100	-0.92189200
H	-3.94029600	-3.56027800	-1.50650800
H	-3.12385600	-4.87405500	-2.35193800
C	4.04305200	-2.36570100	-3.39431900
H	4.34112300	-3.07772200	-4.16646500
H	4.79916700	-1.57685500	-3.37535000
H	4.06366900	-2.87989400	-2.43493400
C	2.66617300	-1.08332000	-5.07893900
H	1.71165100	-0.60391700	-5.29705400
H	3.45012200	-0.32457400	-5.13564400
H	2.85291300	-1.82290300	-5.86077200
C	-0.66077100	-4.52762900	-3.75395100
H	-1.49848300	-5.20562900	-3.84514000
C	0.57254000	-4.89690900	-4.27624000
H	0.69756700	-5.86203400	-4.74953000
C	5.36777900	1.27138300	0.00673500
H	5.37904000	0.23604800	0.33608900
H	5.54982900	1.31482200	-1.06770100
C	2.06605900	3.93952900	-2.50010800
H	1.62293100	4.01517400	-1.50744600
H	3.13009200	3.71234900	-2.38973600
H	1.98271100	4.91244200	-2.98893500
C	2.02760400	2.69897900	-4.71276200
H	1.96180700	3.63277900	-5.27475800
H	3.08576300	2.44623300	-4.61040400
H	1.54814500	1.91969600	-5.30546100
C	6.30969300	2.19215600	0.78444000

H	7.30300200	2.23037300	0.33980700
H	6.41000200	1.85222000	1.81621600
C	4.10801500	3.16548000	0.86278100
H	3.42650000	3.81492100	0.32162000
H	3.79228700	3.09185600	1.90327700

170
5·LiEt

Mo	-1.07115400	0.04411400	0.08358300
Mo	1.07153800	-0.04436200	-0.08330300
Li	-0.37113600	-1.99924200	-1.83536300
Li	0.37128300	1.99813700	1.83651600
N	-1.11003800	-1.52670000	1.66469300
N	1.23928900	-1.56733000	1.54207500
O	-0.30871700	-3.35475900	-3.16964600
C	0.08027300	-1.99453200	2.06809600
H	0.10739300	-2.74663700	2.85494200
C	2.44249400	-2.03972000	2.21191200
C	2.71283700	-1.67088500	3.55873600
C	-2.59815900	-3.52630100	1.93460700
C	-2.28030400	-2.17352500	2.24256900
C	-3.76960600	-1.02145600	-0.83371800
H	-4.52969500	-1.60374000	-1.37154500
H	-3.98589800	0.03005600	-1.03274700
H	-3.93562400	-1.20047700	0.23037900
C	-2.32780400	-1.39442500	-1.25392100
C	1.78568500	-0.79954100	4.41186400
H	1.00873100	-0.39293400	3.76572200
C	3.36117000	-2.88063600	1.52511400
C	-1.71686000	-4.43690100	1.07639500
H	-0.94744200	-3.82298200	0.61035700
C	-2.80694900	-0.04059000	3.61106800
H	-1.90790200	0.28888400	3.09652700
C	4.52049700	-3.30434500	2.18222900
H	5.21938800	-3.94235400	1.66184900
C	-3.12583800	-1.45159500	3.12497000
C	3.08251000	-3.40247600	0.11752000
H	2.47010400	-2.66209200	-0.39476500
C	2.52682300	0.39462500	5.05628800
H	3.23677600	0.06463600	5.81696600
H	1.80834700	1.05680300	5.54651300
H	3.07884900	0.96834400	4.31216900
C	-2.53208400	-0.02843200	5.13156300
H	-3.42221400	-0.30625100	5.70052600
H	-2.23052500	0.97063400	5.45920600
H	-1.73834100	-0.72684000	5.39487800
C	-3.76398400	-4.08978300	2.46831200
H	-4.00807300	-5.11686800	2.23328600
C	4.79091800	-2.93829800	3.49480300
H	5.69320000	-3.28054400	3.98447400
C	1.10117700	-1.63220600	5.52141200
H	0.49222900	-2.43572600	5.10662400
H	0.45745000	-0.99856200	6.13490700
H	1.84618400	-2.08693500	6.17753500
C	3.88622900	-2.13389000	4.16907500
H	4.09122800	-1.85587200	5.19394900
C	-3.92824100	0.95538800	3.25732300
H	-4.11126000	0.96964200	2.18408200
H	-3.65230000	1.96691100	3.56340400
H	-4.86582400	0.70320300	3.75789600
C	-2.50247600	-5.13642500	-0.05559600
H	-3.21760500	-5.86049700	0.33809200
H	-1.81117900	-5.68185900	-0.70322000
H	-3.05363700	-4.41762000	-0.66126300
C	-1.01791500	-5.50611200	1.94989600
H	-0.39250300	-5.05340000	2.71992300
H	-0.38752200	-6.15421200	1.33762300
H	-1.75719800	-6.13372800	2.45179900
C	-4.28271900	-2.06215500	3.62151100
H	-4.92677600	-1.50473200	4.28807400
C	-4.61378600	-3.36923400	3.29487800
H	-5.51286700	-3.82451500	3.68898700
C	-1.45845700	-4.07008400	-3.78276900
H	-2.25012900	-3.34323000	-3.93744600
H	-1.79587300	-4.83134600	-3.08011100
C	4.35352000	-3.62734400	-0.72461000
H	4.99949800	-2.74886900	-0.72451200
H	4.07897300	-3.83920400	-1.76005800
H	4.93871000	-4.47793500	-0.36798900
C	2.26293700	-4.71039000	0.18569900
H	2.82591100	-5.49586500	0.69630500
H	2.02121300	-5.06839800	-0.81938500
H	1.33051900	-4.56000200	0.72502600
C	-0.90237000	-4.68272700	-5.06862000
H	-1.45426400	-5.57192500	-5.36981600
H	-0.94437900	-3.96063700	-5.88550500
C	0.95458900	-3.79969400	-3.81651500
H	1.66147900	-4.04279200	-3.02836400
H	1.33805600	-2.96499900	-4.40132800
C	0.56306500	-4.99092100	-4.69589300
H	1.20772500	-5.08117800	-5.56890600
H	0.62357000	-5.92230700	-4.13065300
H	-2.21643000	-2.47647600	-1.07789400
H	-2.25924800	-1.24143200	-2.34192100
H	1.20832100	-1.36248900	-1.35697900
N	1.11047700	1.52653500	-1.66430200
N	-1.23886300	1.56699500	-1.54185700
O	0.30788100	3.35364400	3.17080100
C	-0.07983800	1.99427000	-2.06779800
H	-0.10695600	2.74634000	-2.85467800
C	-2.44202900	2.03909200	-2.21198100
C	-2.71202900	1.67000300	-3.55880900
C	2.59835700	3.52631800	-1.93404200
C	2.28071700	2.17349300	-2.24204700
C	3.76990200	1.02175400	0.83448700
H	4.52974100	1.60450800	1.37215700
H	3.98663100	-0.02962200	1.03377400
H	3.93585400	1.20055700	-0.22965900
C	2.32795400	1.39414200	1.25459600
C	-1.78457000	0.79865800	-4.41159900
H	-1.00770400	0.39224700	-3.76523300
C	-3.36101000	2.87989100	-1.52547900
C	1.71693600	4.43668400	-1.07571100
H	0.94767600	3.82257100	-0.60967400
C	2.80789600	0.04056800	-3.61033500
H	1.90873900	-0.28895200	-3.09600500
C	-4.52025400	3.30334500	-2.18291400
H	-5.21936100	3.94131100	-1.66276900
C	3.12643500	1.45169700	-3.12437900
C	-3.08283100	3.40181300	-0.11782700
H	-2.47027700	2.66161900	0.39456600
C	-2.52542200	-0.39569300	-5.05600800
H	-3.23520600	-0.06590600	-5.81693900
H	-1.80675400	-1.05789800	-5.54592000
H	-3.07758800	-0.96933900	-4.31193700
C	2.53352700	0.02806800	-5.13091700

H	3.42383900	0.30578900	-5.69964900
H	2.23212100	-0.97108200	-5.45845700
H	1.73984800	0.72638700	-5.39464800
C	3.76408000	4.09001200	-2.46773200
H	4.00799700	5.11712600	-2.23266400
C	-4.79031700	2.93710400	-3.49550100
H	-5.69253500	3.27915900	-3.98542400
C	-1.09995500	1.63125500	-5.52113500
H	-0.49117200	2.43489500	-5.10633700
H	-0.45604900	0.99760100	-6.13442700
H	-1.84489100	2.08581400	-6.17745300
C	-3.88535700	2.13273300	-4.16946500
H	-4.09010500	1.85452000	-5.19433600
C	3.92923000	-0.95516900	-3.25605400
H	4.11196400	-0.96914400	-2.18276200
H	3.65351700	-1.96681500	-3.56195200
H	4.86691000	-0.70296500	-3.75643400
C	2.50251200	5.13622000	0.05630000
H	3.21749800	5.86046100	-0.33734200
H	1.81117500	5.68146300	0.70404100
H	3.05384800	4.41742400	0.66183100
C	1.01773500	5.50585500	-1.94905700
H	0.39233600	5.05310400	-2.71906700
H	0.38728300	6.15379000	-1.33666700
H	1.75686900	6.13364600	-2.45096100
C	4.28321400	2.06246600	-3.62090000
H	4.92738800	1.50514700	-4.28743500
C	4.61402100	3.36961700	-3.29429600
H	5.51302000	3.82506000	-3.68840300
C	1.45723100	4.06942900	3.78445100
H	2.24696500	3.34175500	3.94510000
H	1.79828000	4.82661100	3.07923400
C	-4.35413200	3.62617900	0.72401700
H	-4.99975500	2.74744300	0.72378900
H	-4.07992100	3.83817900	1.75952900
H	-4.93958000	4.47652700	0.36724500
C	-2.26371900	4.71001900	-0.18574400
H	-2.82683900	5.49532200	-0.69645600
H	-2.02238500	5.06807200	0.81941700
H	-1.33110000	4.55999800	-0.72483200
C	0.89856100	4.68915200	5.06593100
H	1.45009400	5.57981400	5.36344900
H	0.93875500	3.97153600	5.88684600
C	-0.95600400	3.79950600	3.81572600
H	-1.66340500	4.03720100	3.02640200
H	-1.33800400	2.96726100	4.40505400
C	-0.56607100	4.99568200	4.68880500
H	-1.21230700	5.09119100	5.56008900
H	-0.62529600	5.92371200	4.11792000
H	2.21613400	2.47620700	1.07882200
H	2.25930800	1.24083000	2.34255200
H	-1.20824500	1.36228800	1.35710800

155

4·LiPh

Mo	0.14635000	-1.07954100	-0.33616000
Mo	-0.33924200	0.81352300	0.45423200
Li	2.46305000	0.64657900	-0.81299700
H	-1.72752700	-0.67815900	0.22179800
N	-0.35808500	0.05601700	2.52205400
N	0.41584900	-1.95316800	1.61447400
N	-1.06259200	1.52219800	-1.48241500
N	-0.67278600	-0.63252500	-2.29947200
C	0.00829300	-1.22727500	2.66721300
H	-0.05592400	-1.69772500	3.64778200
C	-1.09303500	-1.64454200	-3.25388400
C	-1.17986400	0.59783800	-2.45117500
H	-1.72761200	0.84400100	-3.35793900
C	-1.10659800	0.64565300	3.62953200
C	-2.45852700	-2.03554100	-3.31554500
C	-2.43913200	0.21845300	3.87906800
C	-2.56035600	3.46763500	-1.09732800
C	0.25890600	-4.43017000	1.46749200
C	0.96752300	-3.27084500	1.86694300
C	-0.83563200	3.59305100	-2.84317300
C	-3.33666100	2.74716000	0.00357000
H	-2.94897700	1.72977500	0.07858700
C	-1.49428900	2.86745600	-1.81285900
C	2.23790800	-3.39078200	2.48626800
C	0.34042700	3.03406100	-3.64968900
H	0.64055100	2.08823100	-3.20129500
C	3.08370200	-2.18025900	2.88200800
H	2.53209300	-1.28386700	2.60765700
C	-3.12500500	-0.90306500	3.09557000
H	-2.48647200	-1.17722700	2.25900400
C	-1.09536100	-4.37370200	0.76357700
H	-1.40514600	-3.32892100	0.71784900
C	-1.26988400	4.89083600	-3.13564400
H	-0.78058400	5.44603400	-3.92438100
C	-0.51832300	1.63988000	4.44683400
C	-3.54970600	-1.42624300	-2.43000900
H	-3.07432000	-0.77128100	-1.70345800
C	2.75588900	-4.66939300	2.71880300
H	3.72179800	-4.77027200	3.19558500
C	-3.15647600	0.82973500	4.91465900
H	-4.17173400	0.51215200	5.11049700
C	-2.93929200	4.77544600	-1.42101800
H	-3.75011600	5.23847800	-0.87531900
C	0.94025900	2.05436400	4.30112200
H	1.30580500	1.63428900	3.36941500
C	-3.32928500	-2.15246300	3.98236200
H	-3.98464000	-1.93317500	4.82847000
H	-2.38284900	-2.51833800	4.38387300
H	-3.78752300	-2.95710500	3.40393000
C	-1.28233400	2.21707900	5.46765000
H	-0.83592700	2.97617200	6.09492000
C	0.82741600	-5.68465200	1.71821700
H	0.29175400	-6.57559300	1.41960400
C	-0.13862300	-2.25952200	-4.09817600
C	-4.54707100	-0.58687800	-3.26078200
H	-4.04365900	0.21271600	-3.80659200
H	-5.29623400	-0.13336700	-2.60814400
H	-5.07177800	-1.20627800	-3.99171400
C	-3.14629700	3.43180600	1.37546500
H	-2.09051800	3.50630200	1.64229200
H	-3.64924900	2.86685200	2.16163400
H	-3.55848400	4.44307900	1.37167400
C	-4.47103500	-0.46113700	2.48419700
H	-4.88836900	-1.27023800	1.88098900
H	-4.33863500	0.40467100	1.83686900
H	-5.20586600	-0.20498000	3.25034500
C	-2.82812000	-3.02828100	-4.23031100
H	-3.86492600	-3.33036200	-4.28910200
C	-2.30843400	5.48718500	-2.43273600
H	-2.62334000	6.49430900	-2.67178800
C	2.06134900	-5.81242000	2.34294200

H	2.47906100	-6.79260600	2.53173900
C	-2.18713900	-5.14033400	1.54122800
H	-1.98424500	-6.21309400	1.56382400
H	-3.16007700	-4.99833700	1.06545800
H	-2.25673500	-4.79061700	2.57142400
C	-2.59418100	1.82649200	5.70119700
H	-3.16850100	2.28526800	6.49547500
C	1.33297300	-1.85545300	-4.10295800
H	1.46569000	-1.11523400	-3.31560200
C	-0.56393900	-3.25228400	-4.98782000
H	0.15906500	-3.72418000	-5.63972600
C	-4.83715300	2.62841400	-0.34471700
H	-5.35936300	2.05155900	0.42087100
H	-4.97978400	2.12968400	-1.30434800
H	-5.31306300	3.60941400	-0.40201100
C	-1.89622500	-3.63799900	-5.06058400
H	-2.20561800	-4.40456700	-5.75880100
C	1.46877000	2.16266800	0.64797100
C	-0.99200400	-4.90213200	-0.68495700
H	-0.24511600	-4.35243400	-1.26042200
H	-1.94806500	-4.80457400	-1.20149400
H	-0.70867900	-5.95673600	-0.69825800
C	-4.30704900	-2.49654200	-1.61425900
H	-4.85738800	-3.18695500	-2.25667600
H	-5.02748300	-2.01409200	-0.95041700
H	-3.62024700	-3.07667600	-0.99855600
C	-0.05507900	2.75542100	-5.11813600
H	-0.35384600	3.67684900	-5.62244400
H	-0.88926700	2.05637100	-5.18685100
H	0.78907000	2.33185100	-5.66714200
C	3.34159400	-2.12611300	4.40335400
H	2.40582400	-2.12703800	4.96437800
H	3.89313800	-1.22069600	4.66522700
H	3.92950700	-2.98344500	4.73779900
C	4.41608600	-2.14817600	2.10146100
H	5.04730100	-3.00311800	2.35188300
H	4.97561200	-1.24011100	2.34045800
H	4.23282400	-2.16691800	1.02702000
C	2.70021400	1.82743800	1.27252100
H	2.87151400	0.80341700	1.59050200
C	1.57024600	3.96777600	-3.60545400
H	2.41179700	3.49862100	-4.12070100
H	1.86732900	4.17892000	-2.57846000
H	1.37271200	4.91906200	-4.10243600
C	1.79423000	1.46701700	5.44823000
H	2.84817900	1.71746600	5.30384000
H	1.70502000	0.38092500	5.49318300
H	1.48288800	1.86789100	6.41574200
C	3.69121300	2.77315700	1.55981700
H	4.60492600	2.46351900	2.05355200
C	1.72521200	-1.20430600	-5.44867800
H	1.61906100	-1.90844800	-6.27645000
H	2.76673000	-0.87285300	-5.42974800
H	1.09685300	-0.33918000	-5.66511500
C	1.12066100	3.58361700	4.22525000
H	0.51899500	4.01217000	3.42369700
H	2.16499600	3.82718900	4.02625900
H	0.83872400	4.07217500	5.16048800
C	3.49429800	4.11408000	1.23187000
H	4.24963400	4.85365300	1.46441900
C	1.32303000	3.53651100	0.32079500
H	0.41741700	3.87385600	-0.16763800
C	2.25459500	-3.05043200	-3.77876200
H	2.01418700	-3.47525500	-2.80388300
H	3.30069100	-2.73687900	-3.75785100
H	2.16480500	-3.84143300	-4.52577700
C	2.30129600	4.49135500	0.61017600
H	2.13058200	5.53055500	0.35554300
O	4.00975900	1.03242700	-1.86789000
C	6.05132400	2.40709800	-1.38687200
H	6.08360500	2.03848300	-0.36262500
H	6.40609500	3.44000000	-1.38768200
H	6.74130200	1.82031700	-1.99420300
C	4.60584800	0.01079100	-2.75341800
H	5.22804900	0.51867400	-3.49354100
H	3.77227400	-0.45431800	-3.27340000
C	4.62878500	2.36887900	-1.92911400
H	4.59447900	2.70942400	-2.96714300
H	3.97512500	3.00256800	-1.33670000
C	5.39701100	-1.02683800	-1.97112800
H	4.75473200	-1.52445100	-1.24607200
H	6.23783400	-0.57919800	-1.44296800
H	5.78521900	-1.78549600	-2.65482900
H	1.93712600	-1.07446400	-0.79039300

152

4·LiEt·O_2CH

Mo	7.25534000	7.30285900	5.69415600
Mo	5.40138700	8.33196200	5.92846100
O	6.29074200	5.31631800	5.83030300
O	4.33301400	6.39928900	6.12142800
N	5.62450400	8.37044500	8.15185600
N	7.09828400	7.22255000	3.53113900
N	7.57928900	7.10536600	7.85708100
N	5.04283900	8.31241800	3.73318400
O	8.71201500	11.76764300	5.85300700
C	8.09220400	6.59494500	2.67388900
C	6.66316500	7.67847700	8.64707900
H	6.76526900	7.58347800	9.72760400
C	4.84903400	9.13188600	9.12177900
C	5.44522700	10.21801800	9.82099100
C	5.98653300	7.72547700	2.97891800
H	5.84611000	7.65546300	1.90014000
C	9.18936800	7.35372500	2.19345000
C	8.55831700	6.22842900	8.47920400
C	7.98806600	5.21876200	2.34889200
C	5.53212100	10.61025300	5.97205400
C	3.49445200	8.79668500	9.37650500
C	9.92863600	6.59300100	8.49172000
C	6.79122600	4.35935600	2.74956300
H	6.14437300	4.96401500	3.37810100
C	10.86240900	5.71316600	9.04996200
H	11.90841200	5.98883100	9.05627100
C	5.20323100	10.94635800	2.29094000
H	5.96404800	10.48541600	2.91953800
C	10.18009300	6.70961500	1.44367800
H	11.02221700	7.28143400	1.07720300
C	6.70333100	4.51872000	9.11956200
H	6.09147700	5.22812000	8.56931400
C	6.90530000	10.64670200	9.64471900
H	7.32062300	10.09732200	8.80067200
C	3.87312500	8.85247500	3.04559100
C	8.15602400	4.99378500	9.05708400
C	5.02416000	5.31471400	6.04040400
H	4.51194300	4.36115900	6.15676200
C	3.91434300	10.13963200	2.44793500

C	2.67754700	8.09159700	2.98462000
C	9.31174100	8.86092500	2.40831700
H	8.48517600	9.16937100	3.04621800
C	9.00596700	4.62574000	1.59232200
H	8.93413200	3.57534100	1.34491900
C	10.10201200	5.35428300	1.15043000
H	10.88050100	4.87438900	0.57192800
C	2.80898800	7.59595100	8.73210000
H	3.44401600	7.22838800	7.93361300
C	7.74758600	10.30084700	10.89486200
H	8.78554000	10.61114900	10.75731900
H	7.73815300	9.23088900	11.10386200
H	7.35914700	10.81351700	11.77711200
C	9.13241000	4.15848800	9.61306000
H	8.82979300	3.21911300	10.05535400
C	2.76860300	9.55501100	10.30184800
H	1.73811300	9.29551200	10.50118300
C	7.04312700	12.15402200	9.33041800
H	6.42139500	12.44212300	8.48267400
H	8.08331400	12.39274800	9.09453400
H	6.75233700	12.77031800	10.18249100
C	5.69207500	10.88302700	0.82415700
H	5.84674200	9.85421100	0.49787800
H	6.63307100	11.42400500	0.70689300
H	4.95671100	11.33463800	0.15503300
C	6.18697900	4.48169300	10.57635100
H	6.76093100	3.77485300	11.17917800
H	5.14073400	4.16999300	10.60176700
H	6.26157100	5.46014300	11.05441300
C	10.43754200	7.93002600	7.95921900
H	9.58500900	8.46395900	7.54556900
C	4.67224600	10.93718300	10.74085600
H	5.12190500	11.76046400	11.27932400
C	3.34341100	10.61935000	10.98279200
H	2.76527800	11.18956600	11.69795400
C	10.47715200	4.50131300	9.60635400
H	11.21453500	3.83575000	10.03542900
C	1.53158600	8.67011100	2.42653900
H	0.61644200	8.09516300	2.38904100
C	10.62016500	9.24681800	3.12690600
H	10.68253300	8.76859300	4.10242900
H	10.66440200	10.33021300	3.26870000
H	11.49760000	8.95630600	2.54582200
C	5.98043300	3.92776500	1.50592000
H	5.66365500	4.79032100	0.91710000
H	5.08724300	3.37624900	1.80750900
H	6.56984400	3.27909900	0.85431400
C	11.46082100	7.74003000	6.82022200
H	12.35532000	7.21918500	7.16829000
H	11.77543200	8.70827400	6.42327600
H	11.03263600	7.16189600	6.00129900
C	6.51561700	3.14090800	8.44763900
H	6.86434600	3.16360100	7.41611500
H	5.45804700	2.86433200	8.44936000
H	7.05868400	2.35639800	8.97798000
C	2.61299700	6.62857400	3.41390400
H	3.53765500	6.39758600	3.93045400
C	9.20113000	9.61866200	1.06568300
H	10.04855100	9.39059400	0.41580400
H	9.19279100	10.69839300	1.23528900
H	8.29110400	9.35050000	0.53006500
C	7.20748800	3.12648200	3.57912400
H	7.84279300	2.44944600	3.00391600
H	6.32170700	2.56640500	3.88821700
H	7.74228600	3.42697200	4.47855300
C	2.74058700	10.66792600	1.89687100
H	2.76354100	11.65096300	1.44706600
C	11.04333000	8.78740600	9.09322900
H	10.32448000	8.94522800	9.89741300
H	11.35285600	9.76502700	8.71302000
H	11.92583100	8.31122200	9.52536600
C	5.05919000	12.42231600	2.72050000
H	4.39206300	12.97059700	2.05313900
H	6.03239000	12.91901200	2.67838200
H	4.66774600	12.51201400	3.73258000
C	1.54899200	9.95530200	1.90269900
H	0.65115000	10.38753900	1.48078100
C	2.64937700	6.44548500	9.75258100
H	3.61177600	6.16195700	10.17993900
H	2.21868500	5.56621200	9.26762100
H	1.99031400	6.73433800	10.57507400
C	1.44423400	7.95134500	8.10708000
H	0.71589000	8.26038500	8.85990300
H	1.03446400	7.08009100	7.59364900
H	1.53766400	8.75952700	7.37961300
C	1.46104800	6.33774400	4.39486300
H	1.53425500	6.96919500	5.27781400
H	1.50358300	5.29709500	4.72409700
H	0.48336900	6.49866300	3.93505500
C	4.04431000	11.01189300	6.12279200
H	3.90761900	12.09992400	6.16398600
H	3.61801700	10.61288500	7.04510700
H	3.43869700	10.65005100	5.28965900
Li	7.63613400	10.20603300	5.79791200
C	2.51272600	5.70804400	2.17526000
H	1.58115200	5.87896400	1.63092300
H	2.53594100	4.65890800	2.47958600
H	3.33859200	5.87912900	1.48348400
H	8.45801600	8.65377900	5.58475100
H	5.91886100	11.11623400	5.07434300
H	6.06192500	11.03728300	6.83654200
C	10.14524700	11.78862500	6.25501500
H	10.71606900	11.30006000	5.46761700
H	10.23808300	11.21975800	7.17616300
C	10.50325600	13.27041200	6.40341100
H	11.55197500	13.45922100	6.17954300
H	10.30492300	13.61425900	7.41954200
C	9.55044400	13.96358900	5.40715500
H	9.94193500	13.89112700	4.39135800
H	9.39574300	15.01610300	5.63941200
C	8.26250000	13.15103100	5.54478400
H	7.64323600	13.49695700	6.37210800
H	7.66414900	13.10780000	4.6390430

10. References

1. Cordero, B.; Gómez, V.; Platero-Prats, A. E.; Revés, M.; Echeverría, J.; Cremades, E.; Barragán, F.; Alvarez, S. Covalent radii revisited. *Dalton Trans.* **2008**, 2832-2838.

2. Pyykkö, P. Additive covalent radii for single-, double-, and triple-bonded molecules and tetrahedrally bonded crystals: a summary. *J. Phys. Chem.* **2015**, *119*, 2326-2337.

3. Pauling, L. in The Nature of Chemical Bond, 3rd edition, Cornell University Press, New York, USA, **1960**.

4. Cotton, F. A.; Murillo, L. A.; Walton, R. A. in Multiple Bonds Between Metal Atoms, Springer, New York, **2005**.

5. Eisenhart, R. J.; Clouston, L. J.; Lu, C. C. Configuring bonds between first-row transition metals. *Acc. Chem. Res.* **2015**, *48*, 2885-2894.

6. Ekkert, O.; White, A. J. P.; Crimmin, M. R. Trajectory of Approach of a Zinc–Hydrogen Bond to Transition Metals. *Angew. Chem. Int. Ed*. **2016**, *55*, 16031-16034.

7. Butler, M. J.; Crimmin, M. R. Magnesium, zinc, aluminium and gallium hydride complexes of the transition metals. Chem. Comm. **2017**, *53*, 1348-1365.

8. Suresh, C. H.; Koga, N. A consistent approach toward atomic radii. J. Phys. Chem. A. 2001, 105, 5940-5944.

9. Pörschke, K. –R.; Jonas, K.; Wilke, G.; Benn, R.; Mynott, R.; God-dard, R.; Krüger, C. Zur Lewis-Acidität von Nickel (0), I. Methyl-lithium-Komplexe von Nickel (0). *Chem. Ber*. **1985**, *118*, 275-297.

10. Chiu, M.; Hoyt, H.; Michael, F.; Bergman, R.; van Halbeek, H. Synthesis, structural characterization, and quantitative basicity studies of lithium zirconimidate complexes. *Angew. Chem. Int. Ed.* **2008**, *47*, 6073-6076.

11. Spencer, M. D.; Morse, P. M.; Wilson, S. R.; Girolami, G. S. Preparation, catalytic reactivity, and x-ray crystal structure of the first Group 4 alkyl/alkene complexes. The first structural models of the key Ziegler-Natta catalytic intermediate. *J. Am. Chem. Soc.* **1993**, *115*, 2057-2059.

12. Olmstead, M. M.; Power, P. P.; Shoner, S. C. Isolation and x-ray crystal structures of the homoleptic, sigma-bonded transition-metal aryl complexes [(LiEt2O)4VPh6] and [(LiEt2O)3CrPh6]. *Organometallics* **1988**, *7*, 1380-1385.

13. Nattmann, L; Lutz, S.; Ortsack, P.; Goddard, R.; Cornella, J. A Highly Reduced Ni–Li–Olefin Complex for Catalytic Kumada–Corriu Cross-Couplings, *J. Am. Chem. Soc.* **2018**, *140*, 13628-13633.

14. Sheldrick, G. M. SADABS, *Program for Empirical Absorption Correction of Area Detector Data*. Göttingen: University of Göttingen; **1996**.

15. Sheldrick, G. M. SHELXTL, version 6.14. *Program for solution and refinement of crystal structures*, Universität Göttingen, Germany, **2000**.

16. Perez-Jimenez, M.; Curado, N.; Maya, C.; Campos, J.; Ruiz. E.; Alvarez, S.; Carmona, E. *Chem. Eur. J.* **2021**, 10.1002/chem.202004948.

17. Perez-Jimenez, M.; Curado, N.; Maya, C.; Campos, J.; Jover, J.; Alvarez, S.; Carmona, E. *J. Am. Chem. Soc.* **2021**, 10.1021/jacs.1c01602.

18. McKeever, L. D.; Waack, R.; Doran, M. A.; Baker, E. B. *J. Am. Chem. Soc.* **1969**, *91*, 1057-1061.

19. Negishi, E.; Swanson, D. R.; Rousset, C. J. *J. Org. Chem.* **1990**, *55*, 5406-5409.

20. Frisch, M. J.; Trucks, G. W.; Schlegel, H. B.; Scuseria, G. E.; Robb, M. A.; Cheeseman, J. R.; Scalmani, G.; Barone, V.; Mennucci, B.; Petersson, G. A.; Nakatsuji, H.; Caricato, M.; Li, X.; Hratchian, H. P.; Izmaylov, A. F.; Bloino, J.; Zheng, G.; Sonnenberg, J. L.; Hada, M.; Ehara, M.; Toyota, K.; Fukuda, R.; Hasegawa, J.; Ishida, M.; Nakajima, T.; Honda, Y.; Kitao, O.; Nakai, H.; Vreven, T.; Montgomery, J. A. Jr.; Peralta, J. E.; Ogliaro, F.; Bearpark, M.; Heyd, J. J.; Brothers, E.; Kudin, K. N.; Staroverov, V. N.; Keith, T.; Kobayashi, R.; Normand, J.; Raghavachari, K.; Rendell, A.; Burant, J. C.; Iyengar, S. S.; Tomasi, J.; Cossi, M.; Rega, N.; Millam, J. M.; Klene, M.; Knox, J. E.; Cross, J. B.; Bakken, V.; Adamo, C.; Jaramillo, J.; Gomperts, R.; Stratmann, R. E.; Yazyev, O.; Austin, A. J.; Cammi, R.; Pomelli, C.; Ochterski, J. W.; Martin, R. L.; Morokuma, K.; Zakrzewski, V. G.; Voth, G. A.; Salvador, P.; Dannenberg, J. J.; Dapprich, S.; Daniels, A. D.; Farkas, O.; Foresman, J. B.; Ortiz, J. V.; Cioslowski, J.; Fox, D. J. Gaussian 09, Revision D.01, Gaussian, Inc., Wallingford CT, **2013**.

21. Lee, C.; Yang W.; Parr, R. G. *Phys. Rev. B*, **1988**, 785-789.

22. Miehlich, B.; Savin, A.; Stoll, H.; Preuss, H. *Chem. Phys. Lett.* **1989**, 200-206.

23. Becke, A. D. *J. Chem. Phys.* **1993**, 5648-5652.

24. Schaefer, A.; Horn, H.; Ahlrichs, R. *J. Chem. Phys.* **1992**, *97*, 2571-2577.

25. Schaefer, A.; Huber, C.; Ahlrichs, R. *J. Chem. Phys.* **1994**, *100*, 5829-5835.

26. Ahlrichs, R.; May, K. *Phys. Chem. Chem. Phys.* **2000**, *2*, 943-945.

27. *NBO 7.0.* Glendening, E. D.; Badenhoop, J. K.; Reed, A. E.; Carpenter, J. E.; Bohmann, J. A.; Morales, C. M.; Karafiloglou, P.; Landis, C. R.; Weinhold, F. Theoretical Chemistry Institute, University of Wisconsin, Madison, **2018**.
